# Construction of microRNA functional families by a mixture model of position weight matrices

**DOI:** 10.7717/peerj.199

**Published:** 2013-10-31

**Authors:** Je-Keun Rhee, Soo-Yong Shin, Byoung-Tak Zhang

**Affiliations:** 1 Interdisciplinary Program in Bioinformatics, Seoul National University, Seoul, Korea; 2 Department of Biomedical Informatics, Asan Medical Center, Seoul, Korea; 3 School of Computer Science and Engineering, Seoul National University, Seoul, Korea

**Keywords:** Mixture model, MicroRNA, EM algorithm, Machine learning, Position weight matrix, Sequence analysis

## Abstract

MicroRNAs (miRNAs) are small regulatory molecules that repress the translational processes of their target genes by binding to their 3′ untranslated regions (3′ UTRs). Because the target genes are predominantly determined by their sequence complementarity to the miRNA seed regions (nucleotides 2–7) which are evolutionarily conserved, it is inferred that the target relationships and functions of the miRNA family members are conserved across many species. Therefore, detecting the relevant miRNA families with confidence would help to clarify the conserved miRNA functions, and elucidate miRNA-mediated biological processes. We present a mixture model of position weight matrices for constructing miRNA functional families. This model systematically finds not only evolutionarily conserved miRNA family members but also functionally related miRNAs, as it simultaneously generates position weight matrices representing the conserved sequences. Using mammalian miRNA sequences, in our experiments, we identified potential miRNA groups characterized by similar sequence patterns that have common functions. We validated our results using score measures and by the analysis of the conserved targets. Our method would provide a way to comprehensively identify conserved miRNA functions.

## Introduction

MicroRNAs (miRNAs) are a kind of small noncoding RNA which mediate a wide variety of biological processes, including development, differentiation, and metabolism (Ambros, 2004; Bartel, 2004; Bushati & Cohen, 2007). The molecules regulate gene expression by repressing the translation of the target mRNAs or by directly cleaving them, with the RNA-induced silencing complex (RISC). In this regulatory process, nucleotide positions 2–7 of the miRNAs play an important role in the selection of target gene, and are known as seed regions (Bartel, 2009).

The conservation of miRNAs and their targets has been reported previously (Altuvia et al., 2005). For example, the relationship between let-7 miRNA and lin-41, one of its targets, is broadly conserved in many species, and let-7 and lin-4 miRNAs also bind to a conserved target gene, lin-28, in mammals and nematodes (Pasquinelli et al., 2000; Moss & Tang, 2003). Several studies have also shown that a number of miRNA-target relationships are conserved in plants (Floyd & Bowman, 2004; Axtell & Bartel, 2005). Because the target genes are identified based on the binding of the conserved seed sequences of the miRNAs, the target mRNAs and their functions are considered to be evolutionarily conserved among the seed-sharing miRNAs (Lewis, Burge & Bartel, 2005; Friedman et al., 2009).

One approach to defining the miRNA functions is to search for miRNAs that are differentially expressed under specific conditions. Various analyses have successfully identified the miRNAs associated with a particular biological state using miRNA expression profiles (Chen et al., 2006; Liu et al., 2012; Zhang et al., 2012; Tzur et al., 2008). Recently, a study based on an analysis of miRNA profiles with a deep sequencing approach showed that the let-7 family, well-conserved miRNA sequences, is highly expressed in human embryonic stem cells and shares downstream targets (Koh et al., 2010). However, many researchers have demonstrated that the expression patterns of conserved miRNAs are not strictly conserved (Ason et al., 2006; Bak et al., 2008). Moreover, changes in their expression levels might be not an origin necessarily causing the particular biological changes but a result from specific circumstances or environmental conditions.

Another way of investigating miRNA functions is to use a low-level animal model or other species. Using transgenic or knock-out experiments, several studies have identified the biological roles of miRNAs (Costinean et al., 2006; Park, Choi & McManus, 2010). Lindow et al. studied orthologues of the targets of conserved miRNAs to identify conserved regulatory interactions between miRNAs and their targets in different species (Lindow et al., 2007). Recently, an experiment was designed to study the regulatory functions of miRNAs for a pathway by identifying conserved miRNAs and their targets in humans and Drosophila (Hyun et al., 2009).

Although the functions of several miRNAs have been reported in previous studies, the functions of the majority of miRNAs remain poorly understood. To effectively advance the research into miRNA functions using conservation information, a careful construction of miRNA functional families is essential. Here, we describe a method for identifying conserved sets of miRNAs to clarify their conserved functions. In this paper, we define the miRNA functional family as a set of miRNAs which share common functions. These miRNA families are represented by their sequences and share their target relationships. Therefore, a study of the miRNA functional family should extend our understanding of the shared functions of miRNAs across species.

We develop a method of identifying miRNAs which perform similar function based on a mixture model of position weight matrices (PWMs) derived from miRNA sequences. The PWM is a commonly used representation model in biological sequence analyses, constructed by calculating the frequency of each specific base (A, T, G and C) at each nucleotide position in the motif sequence sets. The PWM model has been successfully applied to diverse problems in DNA and protein sequence analysis, and has demonstrated its usefulness in identifying functional sequence elements (Bailey & Elkan, 1995; Hannenhalli & Wang, 2005; Orenstein, Linhart & Shamir, 2012).

In this work, we systematically generated the PWMs from whole mammalian miRNA sequences using a mixture model. The mixture model is a type of probabilistic model for density estimation based on underlying data with mixed distributions. The mixture model has been known to capture the dominant patterns in samples by component distributions (Houseman et al., 2008; McNicholas & Murphy, 2010; Costa, Boccignone & Ferraro, 2012; Melnykov & Maitra, 2010). Therefore, it is appropriate to use a mixture model to identify the sequence patterns and to group similar miRNAs from whole miRNA sequences that contain different bases. Given a set of miRNAs, our approach finds the miRNA sequence profiles in subclasses and constructs the PWMs by estimating the mixing probabilities. Our experimental results show the characteristics of the consensus sequences, with accurate modeling of the base distributions at each position in the PWM. The results confirm that our method can help to identify miRNA functions by collecting similar sequences, and provides an overview of the evolutionarily and functionally related miRNA groups for future functional analyses.

## Materials & Methods

### MicroRNA sequences and position weight matrix

We collected the mature miRNA sequences from miRBase (release 14) (Griffiths-Jones et al., 2008; Kozomara & Griffiths-Jones, 2011) and extracted the mammalian miRNAs from these. We selected for the analysis of the 6-mers at nucleotide positions 2–7, the seed regions, in the mature sequences. Then, we constructed the PWMs, which are scoring matrices weighted according to a specific position in the given seed sequences, with a computational learning approach. The PWMs for our experiments were initialized by randomly and repeatedly extracting sequences of six nucleotides from human genome sequences.

### Mixture model of PWMs and the expectation–maximization (EM) algorithm

We developed a mixture model of position weight matrices to construct miRNA families, and estimated the model parameters with an EM algorithm to maximize the likelihood of the model.

Suppose that *X* = {*X*_1_, *X*_2_, …, *X_i_*, …, *X_N_*} is a dataset of *N* miRNA sequences, and each sequence length |*X_i_*| is *L*. When the model is set as having *k* PWMs, the matrices are denoted by *W* = {*W*_1_, *W*_2_, …, *W_N_*}, which is a set of 4 × *L* position weight matrices derived from miRNA seed sequences. The probability of sequence data *X* is then represented as:

\begin{eqnarray*} \displaystyle P(X\vert W)&=&\displaystyle \sum _{i=1}^{k}{\lambda }_{i}P(X\vert {\lambda }_{i},{W}_{i})\nonumber\\ \displaystyle &=&\displaystyle \sum _{i=1}^{k}{\lambda }_{i}\prod _{m=1}^{N}\prod _{v=1}^{L}{W}_{i}[u={X}_{m v},v], \end{eqnarray*}

where λ_*i*_ is a weight value for the *i*-th PWM, and *X_mv_* is the *v*-th base symbol in the *m*-th miRNA sequence. *W_i_*[*u*, *v*] is the value of index (*u*, *v*) in the *i*-th PWM, and *W_i_*[*u* = *X_mv_*, *v*] is the probabilistic value matched to the base symbol of *X_mv_* in the index (*u*, *v*) of the matrix *W_i_*. The sum of λs should be 1, i.e.,:

\begin{eqnarray*} \sum _{i=1}^{k}{\lambda }_{i}=1\quad ({\lambda }_{i}\geq 0). \end{eqnarray*}

To show assignment of the data samples to the model, we introduce a hidden variable *z_mi_* indicating the probability that an input sequence *X_m_* is represented by a PWM *W_i_*. Given these data, the expected log-likelihood to be maximized by the learning process in the PWM-mixture model is given by:

\begin{eqnarray*} \log L(W,\, \lambda \vert X)=\sum _{m=1}^{N}\sum _{i=1}^{k}E({z}_{m i})\log (P({X}_{m}\vert {W}_{i}){\lambda }_{i}), \end{eqnarray*}

*L*(⋅) is a likelihood function in the model, and *E*(⋅) is an expected value. The parameters of the model are estimated with the EM algorithm. The EM algorithm searches for the maximum value of the likelihood by iteratively repeating the E-step and M-step until convergence is achieved.

The E-step calculates the expected value of the hidden variable *z_mi_* as follows:

\begin{eqnarray*} \displaystyle E({z}_{m i})&=&\displaystyle P({z}_{m i}=1\vert {X}_{m},{W}_{i},{\lambda }_{i})\nonumber\\ \displaystyle &=&\displaystyle \frac{P({X}_{m}\vert {W}_{i}){\lambda }_{i}}{\sum _{j=1}^{k}P({X}_{m}\vert {W}_{j}){\lambda }_{j}}. \end{eqnarray*}

*E*(*z_mi_*) is the posterior probability that a miRNA seed sequence *X_m_* is fitted in the position weight matrix *W_i_*.

The M-step computes the parameters, *W* and λs, to maximize the log-likelihood by

\begin{eqnarray*} \hat {{\lambda }_{i}}=\frac{1}{N}\sum _{m=1}^{N}E({z}_{m i}), \end{eqnarray*}

\begin{eqnarray*} {W}_{i}[\hat {u},v]=\frac{\sum _{m=1}^{N}{W}_{i}[u={X}_{m v},v]E({z}_{m i})}{\sum _{b\in \{ A,U,G,C\} }\sum _{m=1}^{N}{W}_{i}[u=b,v]E({z}_{m i})}. \end{eqnarray*}

Here, $\hat {\cdot }$ is the estimate of the parameters, and *N* is the sample size.

The algorithm is then run iteratively until the marginal likelihood is maximized. When the learning is finished, a miRNA sequence *X_m_* is assigned to the *i*-th group among the *k* groups, according to the hidden variable *z_mi_* that has the maximum value.

### Score functions for validation

To interpret and validate our grouping results, we used two approaches to score the miRNA sequence sets in each cluster. The first scoring function, the match score, was originally developed to search transcription factor binding sites in DNA sequences (Kel et al., 2003). The function *Score_M_* is calculated as follows:

\begin{eqnarray*} {\mathit{Score}}_{M}({X}_{M})=\frac{\sum _{v=1}^{L}I(v){f}_{v,b}-\sum _{v=1}^{L}I(v)f_{v}^{\mathrm{min}}}{\sum _{v=1}^{L}I(v)f_{v}^{\mathrm{max}}-\sum _{v=1}^{L}I(v)f_{v}^{\mathrm{min}}}, \end{eqnarray*}

where *f*_*v*,*b*_ is the frequency of nucleotide *b* (*b*∈{*A*, *T*, *G*, *C*}) at position *v*, and ${f}_{v}^{\mathrm{min}}$ is the frequency of the nucleotide that is the lowest frequency at position *v*, and ${f}_{v}^{\mathrm{max}}$ is the highest at the position. *I*(*v*) represents the conservation of position *v* in a matrix, defined as:

\begin{eqnarray*} I(v)=\sum _{b\in \{ A,U,G,C\} }{f}_{v,b}\log (4{f}_{v,b}). \end{eqnarray*}

The information value *I*(⋅) makes that mismatches in highly conserved regions are suppressed, whereas mismatches in less conserved regions are relatively acceptable.

The other score function is the silhouette measure, first described by Rousseeuw (1987). The score, *Score_S_*, can be used to determine how tightly grouped all the datasets in the cluster are. The score function is defined as:

\begin{eqnarray*} {\mathit{Score}}_{S}({X}_{m})=\frac{\beta ({X}_{m})-\alpha ({X}_{m})}{\max \{ \alpha (X_{m}),\beta ({X}_{m})\} }, \end{eqnarray*}

where α(*X_m_*) is the average distance of *X_m_* to all the other sequences in the same cluster, and β(*X_m_*) is the minimum value for the average distance of *X_m_* to every other single cluster. The score value varies between −1 and 1. If *Score_S_*(*X_m_*) is close to 1, it means that the sequence *X_m_* is well-matched to its own cluster, and dissimilar to the other neighboring clusters. The dissimilarity between two sequences is calculated as the Hamming distance.

### Analysis of the functional relationships in each group

To evaluate the biological meaning of our results, it is necessary to analyze the functional relationships among the miRNAs in the same group. We predicted the target genes of the miRNAs within each group using microCosm Targets release version 5. We chose for further analyses the target genes that were bound by more than half the miRNAs within each group.

We investigated the biological process and molecular function categories of Gene Ontology (GO), and the entries for the KEGG pathways enriched with the target genes in each group, to verify the functional relationships among the human miRNAs assigned to the same group. These analyses were conducted using the DAVID Bioinformatics Resources (Huang, Sherman & Lempicki, 2009).

Next, we looked for the shared functions of the conserved miRNAs and their targets across species. We extracted information for homologues between human and mouse genes from the Ensembl database (Flicek et al., 2013), and then determined whether the miRNA members in the same group shared the conserved targets and their biological roles.

## Results

### miRNA functional family construction

We ran our algorithm independently multiple times with random starts on the same datasets, and with a varying number of clusters (5–100). For each number of clusters, we repeated the algorithm 10 times, because of the possible existence of many local maxima for the mixture model, and we selected the one that maximized the likelihood value. With the highest likelihood value, the chosen number of clusters was 81. All the grouping results are shown in Table S1.

We computed two score measures, *Score_S_* (silhouette score) and *Score_M_* (match score), to validate our grouping results (see Methods section). We calculated the average values for each group, and then compared them with the random and hierarchical clustering results. To confirm that our approach identified relevant miRNA families, we also compared the results with the family information in miRBase (Griffiths-Jones et al., 2008; Kozomara & Griffiths-Jones, 2011). In miRBase, the miRNA families have already been defined, and several of them are known to be sequentially and functionally conserved. We compared our family construction results with the miRBase family information, random groupings and hierarchical clustering results using the average *Score_S_* and *Score_M_* values. Table 1 shows that the *Score_S_* and *Score_M_* values for our results are high. The average *Score_M_* for our results is similar to that for the miRBase families, and the *Score_S_* is much better in our results. The match score, *Score_M_* simply evaluates how well the sequences are represented by the PWM in their own group, and does not explain how well the similar data are collected together by dividing the incompatible instances into several groups, because the score does not measure the differences with samples in other groups. Therefore, the scores for our results indicate that our approach can be used as a way to construct a sequence-based family, by assigning similar miRNA sequences to an identical group well, while assigning dissimilar sequences to different sets.

**Table 1:** Comparison of the average scores for all miRNA sequences.

	*Score_S_*	*Score_M_*
Random	−0.065	0.592
miRBase	0.092	0.919
Hierarchical clustering	0.126	0.892
Our approach	0.144	0.892

**Table 2:** Examples of the enrichments of miRBase family members in our groups.

AC number	miRNA family ID	Group ID	Enrichments
MIPF0000002	let-7	c10	86/86
MIPF0000006	mir-15	c63	68/68
MIPF0000007	mir-181	c44	52/52
MIPF0000011	mir-19	c13	37/37
MIPF0000013	mir-25	c62	42/42
MIPF0000014	mir-9	c27	16/16
MIPF0000022	mir-7	c35	19/19
MIPF0000024	mir-103	c65	34/35
MIPF0000025	mir-99	c12	38/38
MIPF0000026	mir-218	c48	18/18
MIPF0000027	mir-23	c67	30/31
MIPF0000028	mir-135	c81	25/27
MIPF0000031	mir-196	c36	27/27
MIPF0000034	mir-130	c80	41/44
MIPF0000042	mir-204	c25	27/27
MIPF0000042	mir-26	c20	23/23
MIPF0000046	mir-101	c74	20/20
MIPF0000048	mir-128	c27	16/16
MIPF0000050	mir-153	c14	14/14
MIPF0000051	mir-221	c45	25/25
MIPF0000054	mir-216	c17	22/22
MIPF0000055	mir-194	c39	13/13
MIPF0000058	mir-205	c33	16/16
MIPF0000059	mir-184	c43	13/13
MIPF0000062	mir-214	c51	17/17
MIPF0000063	mir-192	c15	23/23
MIPF0000066	mir-183	c66	15/15
MIPF0000074	mir-105	c28	17/20

Table 2 shows that the well-known miRNA family members defined in miRBase are grouped together in our experiment. For example, all the let-7 miRNA sequences, members of one of the most well-known families, are collected into the same group, cluster 10. The let-7 miRNA family members are known to be highly conserved across species in both their sequences and functions, and the members play roles in tumor suppression, and cell differentiation, proliferation and development (Roush & Slack, 2008). The mir-181 family is grouped into cluster 44 in our experiment. Its members are considered to be oncogenic miRNAs that down-regulate the Tcl1 overexpressed in mature B-cell lymphomas, and Hox protein, a repressor of differentiation processes in mammals (Pekarsky et al., 2006; Naguibneva et al., 2006). The members of the miRNA families that show corresponding results, including let-7, mir-15, mir-181, mir-196, and so on, share similar sequences, and the sequence conservation in our groupings can also be detected with WebLogo (Fig. S1) (Crooks et al., 2004). Our results confirm that our approach effectively groups previously known miRNA family sequences together.

**Figure 1:**
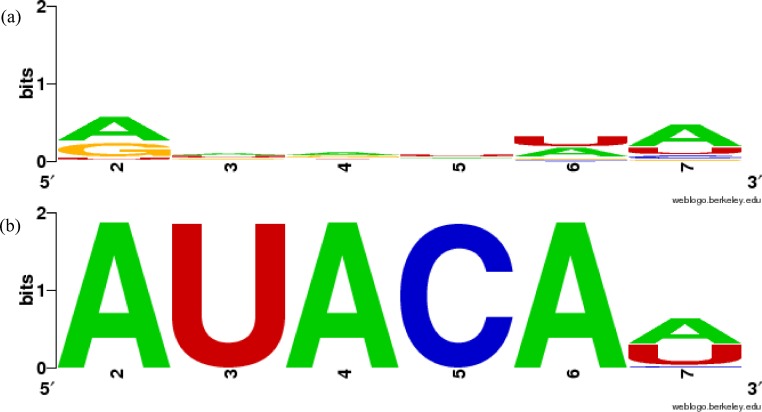
Sequence similarity of groups involving mir-381 in miRBase family and in our results using WebLogo. (A) MIPF0000018 in miRBase, (B) group 52 in our results. The *x*-axis shows the position numbers of the miRNA sequences.

Although many of our groupings are similar to previously constructed miRNA families as shown in Table 2, several results differ. For example, we cannot find sufficiently similar sequence patterns in the sequences of miRBase MIPF0000018 family (mir-154) (Fig. 1A, Table 3). In our experiment, the miRNAs in MIPF0000018 were allocated to several different groups, including clusters 3, 37, and 52, and the similarities between the sequences in our groups were shown more clearly. For further confirmation, we calculated the silhouette measure (*Score_S_*) and match score (*Score_M_*) for MIPF0000018 (Table 3). The *Score_S_* and *Score_M_* for the miRBase family were markedly lower than the results for our groups.

**Table 3:** Allocation of members within the miRBase family MIPF0000018 in our results.

Group ID	# miRNAs	*Score_S_*	*Score_M_*
MIPF0000018	142	−0.669	0.707
c3	82 (42^*^)	−0.087	0.886
c27	432 (12^*^)	−0.366	0.773
c37	58 (11^*^)	−0.146	0.835
c52	19 (10^*^)	0.767	0.990
c71	72 (12^*^)	0.069	0.894
c75	79 (11^*^)	−0.215	0.876

**Notes.**

* is the number of miRNAs in miRBase family MIPF0000018.

In more detail, the miR-381 sequences are included in the MIPF0000018 family with the mir-154 miRNAs. However, in our analysis, the mir-381 sequences are grouped with the mir-466-3p sequences in cluster 52. In this group, the average *Score_S_* is 0.767 and the average *Score_M_* is 0.990, which means that the sequences in the group are highly conserved. These scores show that the mir-381 sequences are sufficiently similar to those mir-466-3p, as expected. The sequence similarities are clearly shown using WebLogo in Fig. 1B. The first five bases of the seed sequences are identical, AUACA in the sequences. From this analysis, it might be supposed that mir-381 was inherited from the same ancestral miRNA gene as mir-466-3p, but not the same as that of mir-154. The inference is reasonable because the mature form of the mir-381 miRNAs is also a 3′-donor sequence in their secondary structure, like mir-466-3p. The property that the miRNA members within one miRBase family are split into several groups in our results is also found in other miRNAs including mir-188 and mir-506 (Table S1). Conversely, MIPF0000013 (mir-25) and MIPF0000069 (mir-32) in miRBase are merged into one group, cluster 62, in our results. Actually, the seed sequences of mir-32 are completely identical to those of mir-25, AUUGCA. Our grouping of cluster 62 is strongly supported by the observation that mir-32 and mir-25 have similar roles. For instance, these miRNAs can lead to cancer by inhibiting apoptosis, because mir-32 suppresses the expression of Bim protein, a pro-apoptotic factor, as mir-25 does in cancer cells (Petrocca et al., 2008; Ambs et al., 2008). Therefore, we established that our method might identify functionally related families.

### Functional analysis for the constructed family

To verify the biological functions of our miRNA groups more comprehensively, we conducted the Gene Ontology (GO) and KEGG pathway enrichment analysis of the human target genes of the miRNAs within each group. The functions of miRNAs are strongly related to the biological roles of their target genes, because the miRNAs recognize the target mRNAs and inhibit their expression. We used the target information produced in microCosm Target version 5. The enrichment results for the target genes in each group are assessed in Tables S2 and S3. We show the most significant part of the results of the GO enrichment analysis using biological process terms in Table 4, and for the KEGG pathways in Table 5. In most of the groups, the target genes are significantly involved in several GO categories. Similarly, we checked the biological relationships among the miRNAs in our groups by the KEGG pathway enrichment analysis, and found frequently and statistically enriched pathways based on the target genes of the members in each group. These results for the GO annotation and KEGG pathway enrichment analyses are consistent with the biological functions of the miRNAs previously reported in the literature. For example, the target genes of cluster 63 were related to the cell cycle in our enrichment analyses. In fact, it is already known that the mir-16 gene family in cluster 63 regulates cell-cycle progression (Linsley et al., 2007; Xia et al., 2009).

**Table 4:** GO Biological Process enrichment of the target genes of the miRNAs in each group.

Group ID	GO accession	GO term	*p*-value
c64	GO:0009987	Cellular process	4.75E−06
c52	GO:0016070	RNA metabolic process	5.84E−05
c64	GO:0006139	Nucleobase, nucleoside, nucleotide and nucleic acid metabolic process	6.09E−05
c52	GO:0016071	mRNA metabolic process	8.88E−05
c55	GO:0046489	Phosphoinositide biosynthetic process	9.26E−05
c56	GO:0006281	DNA repair	9.29E−05
c52	GO:0006396	RNA processing	9.86E−05
c52	GO:0006397	mRNA processing	1.11E−04
c64	GO:0034641	Cellular nitrogen compound metabolic process	1.36E−04
c52	GO:0000087	M phase of mitotic cell cycle	1.56E−04

**Table 5:** KEGG pathway enrichment of the target genes of the miRNAs in each group.

Group ID	Entry	Pathway name	*p*-value
c52	hsa04630	Jak-STAT signaling pathway	0.0022
c64	hsa00230	Purine metabolism	0.0026
c52	hsa04060	Cytokine-cytokine receptor interaction	0.0029
c55	hsa00563	Glycosylphosphatidylinositol (GPI)-anchor biosynthesis	0.0034
c6	hsa00340	Histidine metabolism	0.0051

**Figure 2:**
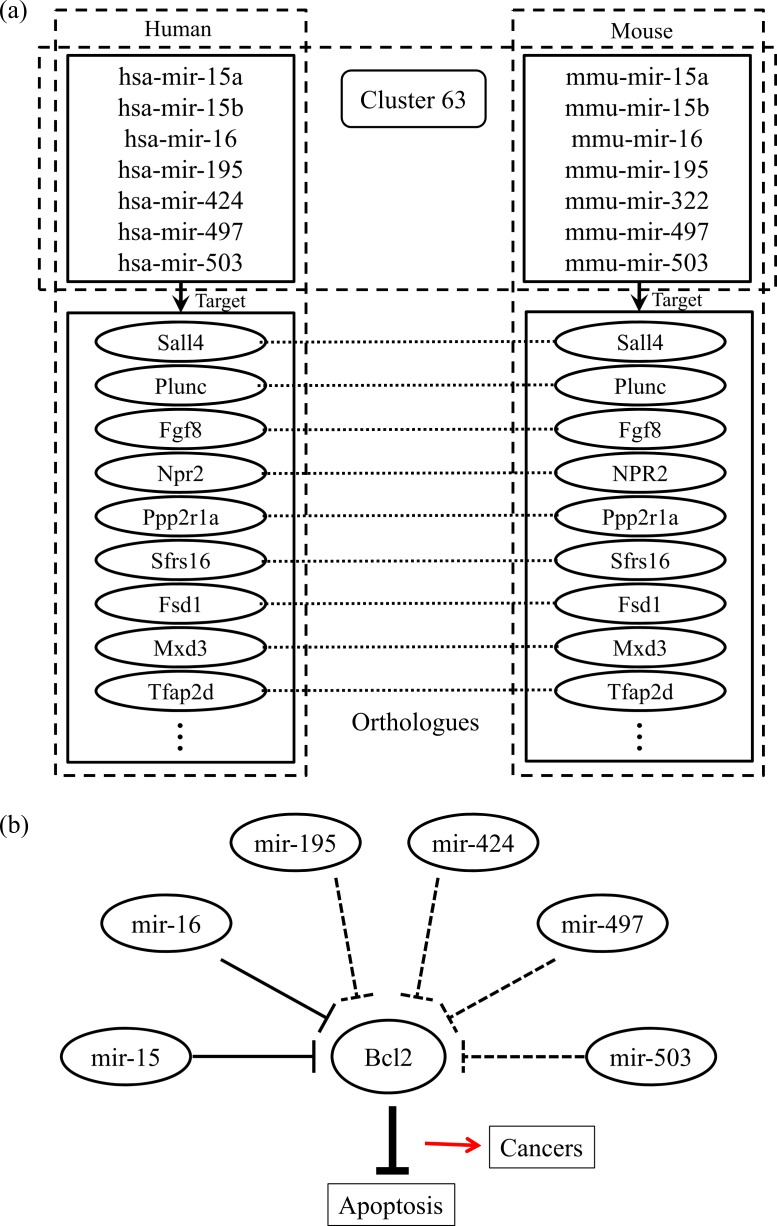
Conserved targets and functional relationships of miRNAs in cluster 63. (A) The miRNA family members in cluster 63 target orthologous genes across species, and this observation implies that functions of the family members are similar. (B) Highly expressed mir-15 and mir-16 induce cell death by targeting the Bcl2 gene, a repressor of apoptosis, but the misregulation of these miRNAs causes cancer. The other miRNAs in cluster 63 might be supposed to have similar regulatory functions.

Finally, we checked the conserved target information for the human and mouse miRNAs categorized into the same group. Figure 2A shows an example of the conserved target analyses in the two different species. The human and mouse miRNAs within cluster 63 share orthologous target genes. These analyses help to clarify the functions of the miRNAs in each group because the target relationships are conserved across most species. Using our miRNA grouping results, we can speculate about their functions based on our knowledge of other miRNAs, with already-known functions, in the same group. As an example, it has previously been shown that the expression of Bcl2 protein is negatively regulated by miR-15 and miR-16, which are assigned to the same group, cluster 63 (Cimmino et al., 2005). Because the Bcl2 protein inhibits cell apoptosis, its overexpression leads to leukemias or other cancers (Cory & Adams, 2002). In our experiment, there were many other miRNAs within the cluster 63, such as mir-322, mir-424, mir-497, and so on, grouped with the miR-15 family members, whose functions are not yet clearly known. From our results, we can infer that these miRNAs may induce cancers by a similar mechanism to that of miR-15 and miR-16 (Fig. 2B). In practice, microCosm predicts that mir-503 binds to the Bcl2 gene transcript. Therefore, our grouping analyses, together with the conservation information, provide clues to the biological effects of functionally unknown miRNAs. Furthermore, the example demonstrates that, as well as identifying the miRNA functions, our approach can help to discover miRNA-mRNA modules in the complex gene regulatory networks and to understand the combinatorial effects of miRNAs in cellular processes.

## Discussion

Understanding gene regulation is still challenging, and action of miRNAs, in particular, may cause the processes to be even more complex and harder to interpret. Much research had been directed towards understanding the gene regulation by miRNAs and identifying their functions, but there have been not many studies that comprehensively and systematically examine conserved information across various species although it is known that miRNA genes are evolutionarily conserved (Shi, Gao & Wang, 2012; Berezikov, 2011; Borenstein & Ruppin, 2006; Li et al., 2010). Furthermore, computational target prediction methods have mainly focused on one-to-one interactions, and the experimental identification and validation of miRNA functions remain time consuming and technically limited. In this study, we have undertaken a fundamental task of the miRNA research in identifying evolutionarily conserved miRNAs, to extend the functional studies of miRNAs.

The previous classification of miRNA family in miRBase was based on the Rfam database (Griffiths-Jones et al., 2008; Kozomara & Griffiths-Jones, 2011; Gardner et al., 2009; Burge et al., 2013). However, it might need to use another definition for functional analysis or target prediction. For example, Friedman et al. (2009) defined 87 miRNA families and the definition was used for target prediction. Also, there has been lots of other research to precisely detect miRNA families using evolutionary information (Guerra-Assunção & Enright, 2012; Gerlach et al., 2009). We constructed miRNA functional families from miRNA seed sequences based on a mixture model of position weight matrices. Our method starts with random PWMs extracted from human genome sequences. Through iterative learning to maximize the likelihood value using the EM algorithm, this method assigns the miRNA sequences to each group and builds PWMs that represent the corresponding conserved sequences in each group, by adjusting the parameters in the mixture model. We have presented results for all the mammalian miRNAs, demonstrating that our approach constructs biologically relevant miRNA families, using score measures and functional analyses of their target genes. We have also shown that these results can facilitate the identification of conserved and biologically related subsets or modules of miRNAs and mRNAs by analyzing the conserved target information in our groups.

Usually, a model selection criterion such as Bayesian information criterion (BIC), is adapted to reduce model complexity when using the maximum likelihood estimation (Schwarz, 1978; Jones, 2011). However, the scheme could not be applied in our experiment. BIC always penalizes multiple clusters since our approach has a relatively huge number of parameters unlike other general models. Moreover, it is difficult to interpret biologically.

Previous works have assumed that each miRNA sequence is contained in only one miRNA family. Unfortunately, it is not possible to clearly know what the true ancestor of each miRNA is and how the sequence has evolved. The imprecise assumptions may limit the study of branched miRNAs. However, our approach has the potential to overcome these restrictions because it assigns the miRNAs to families with a probabilistic value. Although we selected a group of miRNAs by choosing the one with the maximum probabilistic value among hidden variables in our experiment, it is feasible to accommodate overlapping occurrences of miRNAs by modifying the group assignment scheme and to flexibly assign a sequence to various number of clusters. Moreover, by diversifying the number of clusters, it may be likely to find more broadly conserved miRNA groups or more specialized families.

Many miRNAs remain to be identified, and it is not easy to identify the conserved sequence patterns of several miRNAs when the number of corresponding family members is limited. However, with the increasing availability of miRNA sequences, our method can substantially improve the grouping properties with greater precision. Furthermore, the PWMs generated by our method might also be used to search for novel miRNAs in the genome, as in the identification of transcription factor binding sites.

In conclusion, because a large fraction of protein-coding genes is regulated by miRNAs, the systematic and comprehensive search for conserved miRNAs may be a useful way to understand gene regulatory processes and to elucidate the biological functions of miRNAs. Our method should provide a basis for the functional annotation of miRNAs and fundamental insight into the widespread impact of miRNAs.

## Supplemental Information

10.7717/peerj.199/supp-1 Figure S1 Sequence similarities represented by Weblogo in (A) c10, (B) c63, (C) c44 and (D) c36

10.7717/peerj.199/supp-2 Table S1 microRNA lists in each group

10.7717/peerj.199/supp-3 Table S2 GO (Molecular Function) enrichment analyses for target genes in each group

10.7717/peerj.199/supp-4 Table S3 GO (Biological Process) enrichment analyses for target genes in each group
