# Supplementary figures and images for: Construction of microRNA functional families by a mixture model of position weight matrices
Source: PeerJ. 2013 Oct 31;1:e199. doi: 10.7717/peerj.199 (PMC3817585; doi:10.7717/peerj.199)

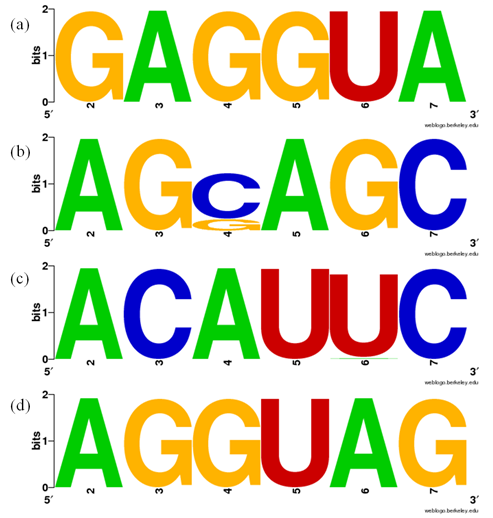

Supplement: Figure S1 [file peerj-01-199-s001.png]
