# Supplementary material for: Construction of microRNA functional families by a mixture model of position weight matrices
Source: PeerJ. 2013 Oct 31;1:e199. doi: 10.7717/peerj.199 (PMC3817585; doi:10.7717/peerj.199)
Supplement: Table S1 [file peerj-01-199-s002.pdf]

Supplemental Table S1. microRNA lists in each group

Group ID	miRNAs
1	mmu-miR-188-5p, mmu-miR-291a-5p, mmu-miR-34b-3p, hsa-miR-188-5p, rno-miR-347, hsa-miR-34c-3p, hsa-miR-362-5p, mmu-miR-362-5p, rno-miR-291a-5p, mml-miR-188, ptr-miR-188, ppy-miR-188, mne-miR-188, ppa-miR-188, hsa-miR-501-5p, rno-miR-501, mmu-miR-543, mmu-miR-291b-5p, rno-miR-664, mmu-miR-703, mmu-miR-501-5p, hsa-miR-875-5p, mmu-miR-875-5p, hsa-miR-543, rno-miR-188, mmu-miR-466l, hsa-miR-664, oan-miR-875, oan-miR-1400, mml-miR-34c-3p, mml-miR-362-5p, mml-miR-501, mml-miR-664, mml-miR-875-5p, cfa-miR-543, cfa-miR-382, ptr-miR-34c, ptr-miR-543, ptr-miR-664, ptr-miR-875, bta-miR-188, bta-miR-362-5p, bta-miR-543, bta-miR-875, cfa-miR-188, cfa-miR-362, cfa-miR-875, bta-miR-2312, mmu-miR-664, mml-miR-543, ptr-miR-664b, rno-miR-362, rno-miR-875, eca-miR-34b-3p, eca-miR-703, eca-miR-543, eca-miR-664, eca-miR-188-5p, eca-miR-362-5p
2	hsa-miR-34a, hsa-miR-223, mmu-miR-34c, mmu-miR-34b-5p, mmu-miR-34a, rno-miR-346, mmu-miR-346, mmu-miR-223, hsa-miR-34c-5p, hsa-miR-346, rno-miR-34c, rno-miR-34a, rno-miR-223, hsa-miR-449a, mmu-miR-449a, rno-miR-449a, cfa-miR-449, ggo-miR-34a, age-miR-34a, ppa-miR-34a, ppy-miR-34a, ptr-miR-34a, mml-miR-34a, sla-miR-34a, lla-miR-34a, mne-miR-34a, mml-miR-223, ptr-miR-223, ggo-miR-223, ppy-miR-223, sla-miR-223, ppa-miR-223, hsa-miR-604, hsa-miR-449b, mmu-miR-449c, bta-miR-34b, bta-miR-34c, mdo-miR-34a, mdo-miR-223, mdo-miR-449, bta-miR-34a, mmu-miR-449b, hsa-miR-885-3p, oan-miR-449c, oan-miR-449b, oan-miR-449a, oan-miR-223, oan-miR-34, mml-miR-34c-5p, mml-miR-346, mml-miR-449a, mml-miR-449b, mml-miR-885-3p, cfa-miR-34a, cfa-miR-34c, ssc-miR-34a, ptr-miR-346, ptr-miR-449a, ptr-miR-449b, ptr-miR-885, bta-miR-223, bta-miR-346, bta-miR-449a, bta-miR-449b, bta-miR-449c, cfa-miR-34b, cfa-miR-223, cfa-miR-346, hsa-miR-2277, bta-miR-2379, mdo-miR-885, ptr-miR-604, eca-miR-346, eca-miR-34, eca-miR-34b-5p, eca-miR-34c, eca-miR-885-3p, eca-miR-449a, eca-miR-223
3	hsa-miR-21, mmu-miR-21, rno-miR-323, mmu-miR-323-3p, mmu-miR-338-5p, hsa-miR-369-3p, hsa-miR-323-3p, hsa-miR-338-5p, rno-miR-21, mmu-miR-410, mmu-miR-464, ssc-miR-21, hsa-miR-410, hsa-miR-487a, mml-miR-21, ptr-miR-21, ggo-miR-21, ppy-miR-21, mne-miR-21, age-miR-21, ppa-miR-21, hsa-miR-487b, mmu-miR-487b, mmu-miR-369-3p, rno-miR-487b, rno-miR-369-3p, hsa-miR-576-5p, hsa-miR-590-5p, hsa-miR-656, bta-miR-21, bta-miR-369-3p, bta-miR-487a, bta-miR-487b, mdo-miR-21, mmu-miR-590-5p, hsa-miR-885-5p, cgr-miR-21, rno-miR-410, hsa-miR-548p, oan-miR-1422a, oan-miR-1422n, oan-miR-21, mdo-miR-1547, mml-miR-323-3p, mml-miR-338-5p, mml-miR-369-3p, mml-miR-410, mml-miR-487a, mml-miR-487b, mml-miR-576-5p, mml-miR-590-5p, mml-miR-624, mml-miR-656, mml-miR-885-5p, cfa-miR-323, cfa-miR-487b, cfa-miR-369, cfa-miR-410, cfa-miR-142, cfa-miR-21, ptr-miR-323, ptr-miR-369, ptr-miR-410, ptr-miR-487a, ptr-miR-487b, ptr-miR-548p, ptr-miR-656, bta-miR-410, bta-miR-656, bta-miR-885, mmu-miR-1941-3p, cfa-miR-200b, cfa-miR-200a, cfa-miR-487a, cfa-miR-885, bta-miR-2439, bta-miR-2284h, eca-miR-21, eca-miR-338-5p, eca-miR-590-5p, eca-miR-885-5p, eca-miR-323-3p, eca-miR-369-3p, eca-miR-410, eca-miR-487a, eca-miR-487b, eca-miR-656
4	mmu-miR-155, hsa-miR-155, hsa-miR-219-2-3p, hsa-miR-376c, rno-miR-10a-3p, rno-miR-219-2-3p, hsa-miR-483-5p, mml-miR-141, ggo-miR-141, ppy-miR-141, ppa-miR-141, hsa-miR-522, mmu-miR-483, mmu-miR-376c, rno-miR-376c, hsa-miR-553, hsa-miR-574-3p, hsa-miR-602, hsa-miR-624, hsa-miR-633, hsa-miR-639, mmu-miR-700, mmu-miR-713, mmu-miR-574-3p, hsa-miR-941, hsa-miR-548g, ptr-miR-510, oan-miR-1327, oan-miR-1422i, oan-miR-219, oan-miR-155, oan-miR-1422j, oan-miR-1399, oan-miR-1410, oan-miR-1421w, oan-miR-1422p, mml-miR-155, mml-miR-219-3p, mml-miR-376c, mml-miR-518e, mml-miR-522, mml-miR-553, mml-miR-633, cfa-miR-155, cfa-miR-574, mmu-miR-1893, hsa-miR-1912, ptr-miR-155, ptr-miR-219-2-3p, ptr-miR-376c, ptr-miR-522, ptr-miR-553, ptr-miR-624, bta-miR-155, bta-miR-219-3p, cfa-miR-141, bta-miR-2302, bta-miR-2474, ptr-miR-602, eca-miR-376c, eca-miR-155, eca-miR-1912
5	mmu-miR-200b, hsa-miR-200b, hsa-miR-200c, mmu-miR-200c, hsa-miR-365, mmu-miR-365, hsa-miR-374a, rno-miR-200c, rno-miR-200b, mmu-miR-409-3p, hsa-miR-429, mmu-miR-429, rno-miR-429, cfa-miR-429, cfa-miR-365, rno-miR-365, hsa-miR-409-3p, hsa-miR-500, rno-miR-374, hsa-miR-548a-3p, hsa-miR-802, mmu-miR-374, mmu-miR-687, mmu-miR-707, bta-miR-200c, bta-miR-200b, mdo-miR-365, mdo-miR-200c, mdo-miR-200b, bta-miR-365-3p, bta-miR-374a, hsa-miR-374b, hsa-miR-548e, hsa-miR-548f, hsa-miR-1244, hsa-miR-1245, hsa-miR-1278, oan-miR-1330, oan-miR-33a-3p, oan-miR-365, oan-miR-200b, oan-miR-1421j, oan-miR-33b-3p, oan-miR-429, oan-miR-1419f, mdo-miR-340, mml-miR-365, mml-miR-374a, mml-miR-374b, mml-miR-409-3p, mml-miR-429, mml-miR-500, mml-miR-548a, mml-miR-802, cfa-miR-200c, cfa-miR-374a, cfa-miR-374b, ptr-miR-1244, ptr-miR-1245, ptr-miR-1278, ptr-miR-365, ptr-miR-374a, ptr-miR-374b, ptr-miR-409, ptr-miR-500, ptr-miR-548a, ptr-miR-548f, ptr-miR-633, ptr-miR-802, bta-miR-374b, bta-miR-429, bta-miR-500, mmu-miR-1949, cfa-miR-802, hsa-miR-2054, bta-miR-2285a, bta-miR-2489, bta-miR-2372, bta-miR-2385-3p, bta-miR-2429, bta-miR-2478, mdo-miR-429, eca-miR-200b, eca-miR-429, eca-miR-200c, eca-miR-365, eca-miR-409-3p, eca-miR-802, eca-miR-374a, eca-miR-374b, eca-miR-500
6	mmu-miR-296-3p, hsa-miR-296-3p, rno-miR-296, mmu-miR-540-5p, hsa-miR-615-5p, mmu-miR-672, mmu-miR-615-5p, rno-miR-672, hsa-miR-1182, oan-miR-1343, oan-miR-1414, mml-miR-296-3p, mml-miR-615-5p, cfa-miR-329a, ptr-miR-1182, ptr-miR-296, bta-miR-296, bta-miR-329b, bta-miR-615, cfa-miR-377, cfa-miR-615, bta-miR-2343, bta-miR-2389, rno-miR-615, eca-miR-615-5p, eca-miR-296, eca-miR-672
7	hsa-miR-17, hsa-miR-20a, hsa-miR-93, hsa-miR-106a, mmu-miR-106a, mmu-miR-106b, mmu-miR-20a, mmu-miR-93, rno-miR-20a, mmu-miR-17, hsa-miR-106b, rno-miR-17-5p, rno-miR-93, rno-miR-106b, hsa-miR-20b, ssc-miR-106a, ssc-miR-20, ggo-miR-17-5p, ggo-miR-20, lca-miR-17-5p, lca-miR-20, age-miR-17-5p, age-miR-20, ppa-miR-17-5p, ppa-miR-20, ppy-miR-17-5p, ppy-miR-20, ptr-miR-17-5p, ptr-miR-20a, mml-miR-17-5p, mml-miR-20a, sla-miR-17-5p, sla-miR-20, lla-miR-17-5p, lla-miR-20, mne-miR-17-5p, mne-miR-20, ggo-miR-106b, age-miR-106b, ppa-miR-106b, ppy-miR-106b, ptr-miR-106b, mml-miR-106b, sla-miR-106b, lla-miR-106b, mne-miR-106b, ggo-miR-106a, age-miR-106a, ppa-miR-106a, mml-miR-106a, ppy-miR-106a, ptr-miR-106a, sla-miR-106a, mne-miR-106a, hsa-miR-519d, mmu-miR-20b, rno-miR-20b-5p, bta-miR-20a, bta-miR-106, bta-miR-20b, bta-miR-17-5p, bta-miR-93, mdo-miR-17-5p, mdo-miR-20, oan-miR-106, oan-miR-20b, oan-miR-20a, oan-miR-17, mml-miR-20b, mml-miR-518a-3p, mml-miR-519d, cfa-miR-20a, cfa-miR-106b, cfa-miR-93, ssc-miR-17, ptr-miR-20b, ptr-miR-519d, bta-miR-106b, cfa-miR-20b, mdo-miR-106, eca-miR-106b, eca-miR-93, eca-miR-17, eca-miR-20a, eca-miR-106a, eca-miR-20b
8	mmu-miR-142-3p, mmu-miR-190, hsa-miR-142-3p, hsa-miR-190, rno-miR-142-3p, rno-miR-190, mml-miR-190a, ptr-miR-190a, ggo-miR-190, ppa-miR-190, hsa-miR-519c-5p, hsa-miR-519b-5p, hsa-miR-526a, hsa-miR-520c-5p, hsa-miR-518d-5p, mmu-miR-542-5p, rno-miR-542-5p, mmu-miR-450a-3p, hsa-miR-651, hsa-miR-2113, mmu-miR-669a, mmu-miR-467b, mdo-miR-142, mmu-miR-190b, hsa-miR-190b, mmu-miR-877, hsa-miR-877, rno-miR-877, mmu-miR-467e, rno-miR-190b, mmu-miR-1188, mmu-miR-467h, hsa-miR-1259, oan-miR-190a, oan-miR-190b, oan-miR-1422m, oan-miR-135a, oan-miR-1406, mdo-miR-1541, mml-miR-142-3p, mml-miR-190b, mml-miR-523b, mml-miR-877, cfa-miR-135a-3p, mmu-miR-1897-5p,

	mmu-miR-1892, ptr-miR-1259, ptr-miR-142, ptr-miR-190b, ptr-miR-526a, bta-miR-190a, bta-miR-190b, bta-miR-877, mmu-miR-1929, mmu-miR-1944, mmu-miR-669l, mmu-miR-669n, mmu-miR-1971, mmu-miR-1981, cfa-miR-190a, cfa-miR-190b, bta-miR-2304, bta-miR-2317, bta-miR-2341, bta-miR-2354, bta-miR-2359, bta-miR-2398, bta-miR-320b, mdo-miR-190a, mdo-miR-190b, ptr-miR-651, eca-miR-1892, eca-miR-190, eca-miR-190b, eca-miR-142-3p, eca-miR-542-5p
9	rno-miR-345-3p, rno-miR-10b, hsa-miR-486-5p, mmu-miR-486, mmu-miR-881, mmu-miR-883b-5p, hsa-miR-892a, rno-miR-881, hsa-miR-1183, oan-miR-1395, mml-miR-486-5p, mml-miR-892, hsa-miR-1914, ptr-miR-1183, ptr-miR-486, ptr-miR-892a, bta-miR-486, cfa-miR-10b, bta-miR-2311, bta-miR-2362, bta-miR-2384, bta-miR-2476, eca-miR-486-5p
10	hsa-let-7a, hsa-let-7b, hsa-let-7c, hsa-let-7d, hsa-let-7e, hsa-let-7f, hsa-miR-98, mmu-let-7g, mmu-let-7i, mmu-miR-202-3p, mmu-let-7d, hsa-let-7g, hsa-let-7i, mmu-let-7a, mmu-let-7b, mmu-let-7c, mmu-let-7e, mmu-let-7f, mmu-miR-98, rno-let-7d, rno-let-7a, rno-let-7b, rno-let-7c, rno-let-7e, rno-let-7f, rno-let-7i, rno-miR-98, ssc-let-7c, ssc-let-7f, ssc-let-7i, mml-miR-98, ptr-miR-98, ggo-miR-98, ppy-miR-98, age-miR-98, ppa-miR-98, hsa-miR-202, bta-let-7f, bta-miR-98, bta-let-7d, bta-let-7g, bta-let-7a, bta-let-7i, mdo-let-7a, mdo-let-7g, mdo-let-7i, mdo-let-7f, mdo-let-7b, mdo-let-7d, bta-let-7b, bta-let-7c, bta-let-7e, oan-let-7b, oan-let-7e, oan-let-7g, oan-miR-98, oan-let-7f, oan-let-7d, mml-let-7a, mml-let-7b, mml-let-7c, mml-let-7d, mml-let-7e, mml-let-7f, mml-let-7g, mml-let-7i, cfa-let-7a, cfa-let-7e, cfa-let-7f, cfa-let-7g, cfa-let-7c, cfa-miR-98, cfa-let-7j, ptr-let-7a, ptr-let-7b, ptr-let-7c, ptr-let-7d, ptr-let-7e, ptr-let-7f, ptr-let-7g, ptr-let-7i, ptr-miR-202, mmu-miR-1961, cfa-let-7b, eca-let-7a, eca-let-7e, eca-let-7g, eca-let-7d, eca-let-7f, eca-let-7c, eca-miR-98
11	mmu-miR-151-5p, rno-miR-151, hsa-miR-151-5p, mml-miR-31, ptr-miR-31, ggo-miR-31, ppy-miR-31, mne-miR-31, ppa-miR-31, hsa-miR-611, hsa-miR-1224-5p, mmu-miR-1224, mmu-miR-665, hsa-miR-765, hsa-miR-665, mmu-miR-669g, ptr-miR-1224-5p, mml-miR-1224, hsa-miR-1289, hsa-miR-1255a, hsa-miR-1255b, oan-miR-1356, oan-miR-1397, oan-miR-1421y, oan-miR-1421ac, oan-miR-1421ah, oan-miR-1421aj, mml-miR-151-5p, mml-miR-611, mml-miR-765, cfa-miR-151, hsa-miR-1909, ptr-miR-1255b, ptr-miR-1289, ptr-miR-665, ptr-miR-765, cfa-miR-665, bta-miR-1224, mmu-miR-2145, bta-miR-2422, mml-miR-665, rno-miR-1224, rno-miR-665, eca-miR-151-5p, eca-miR-1289
12	hsa-miR-99a, hsa-miR-100, mmu-miR-99a, mmu-miR-99b, mmu-miR-100, hsa-miR-99b, rno-miR-99a, rno-miR-99b, rno-miR-100, mml-miR-99a, ptr-miR-99a, ggo-miR-99a, ppy-miR-99a, lla-miR-99a, mne-miR-99a, ppa-miR-99a, ggo-miR-100, age-miR-100, ppa-miR-100, ppy-miR-100, ptr-miR-100, mml-miR-100, sla-miR-100, lla-miR-100, bta-miR-99a, mdo-miR-100, bta-miR-99b, oan-miR-100, oan-miR-99, ssc-miR-99b, mml-miR-99b, cfa-miR-99b, cfa-miR-99a, mmu-miR-1905, ptr-miR-99b, bta-miR-100, eca-miR-100, eca-miR-99b, eca-miR-99a
13	hsa-miR-19a, hsa-miR-19b, mmu-miR-150, hsa-miR-198, mmu-miR-290-5p, mmu-miR-292-5p, mmu-miR-293, mmu-miR-300, hsa-miR-150, mmu-miR-19b, mmu-miR-19a, hsa-miR-371-5p, rno-miR-19b, rno-miR-19a, rno-miR-150, rno-miR-292-5p, rno-miR-300-3p, ssc-miR-19a, ggo-miR-198, age-miR-198, ppa-miR-198, ppy-miR-198, ptr-miR-198, mml-miR-198, sla-miR-198, lla-miR-198, mne-miR-198, ggo-miR-19a, ggo-miR-19b, lca-miR-19a, lca-miR-19b, age-miR-19a, age-miR-19b, ppa-miR-19a, ppa-miR-19b, ppy-miR-19a, ppy-miR-19b, ptr-miR-19a, ptr-miR-19b, mml-miR-19a, mml-miR-19b, sla-miR-19a, sla-miR-19b, lla-miR-19a, lla-miR-19b, mne-miR-19a, mne-miR-19b, hsa-miR-532-3p, mmu-miR-532-3p, hsa-miR-586, hsa-miR-550, hsa-miR-1283, bta-miR-126, bta-miR-150, mdo-miR-19a, mdo-miR-19b, bta-miR-19a, bta-miR-19b, hsa-miR-875-3p, mmu-miR-875-3p, rno-miR-532-3p, mmu-miR-669k, mmu-miR-669h-3p, hsa-miR-1227, oan-miR-1326, oan-miR-19b, oan-miR-460, oan-miR-1368, oan-miR-150, oan-miR-19a, oan-miR-1417, oan-miR-1421ag, mml-miR-150, mml-miR-371-5p, mml-miR-532-3p, mml-miR-550, mml-miR-586, mml-miR-875-3p, cfa-miR-371, cfa-miR-150, cfa-miR-19a, cfa-miR-19b, ptr-miR-1227, ptr-miR-1283, ptr-miR-150, ptr-miR-532, ptr-miR-550, ptr-miR-586, mmu-miR-1950, mmu-miR-1955, mmu-miR-1956, hsa-miR-2276, bta-miR-2299-5p, bta-miR-2303, mdo-miR-150, mdo-miR-460, ptr-miR-373, rno-miR-293, rno-miR-295, eca-miR-150, eca-miR-371-5p, eca-miR-19a, eca-miR-19b, eca-miR-532-3p, eca-miR-545
14	mmu-miR-153, mmu-miR-297a, hsa-miR-153, rno-miR-153, rno-miR-297, hsa-miR-448, mmu-miR-448, rno-miR-448, cfa-miR-448, ssc-miR-153, mml-miR-153, ppy-miR-153, mne-miR-153, ggo-miR-153, rno-miR-376b-5p, hsa-miR-568, hsa-miR-620, hsa-miR-632, hsa-miR-647, mmu-miR-675-3p, mmu-miR-297b-5p, mmu-miR-297c, mmu-miR-568, hsa-miR-297, mmu-miR-669h-5p, hsa-miR-1231, hsa-miR-1261, hsa-miR-1270, oan-miR-1341, oan-miR-153, mml-miR-297, mml-miR-448, mml-miR-568, mml-miR-632, ptr-miR-153, ptr-miR-297, ptr-miR-448, ptr-miR-568, ptr-miR-632, bta-miR-153, bta-miR-448, bta-miR-568, mmu-miR-1940, cfa-miR-153, cfa-miR-376c, cfa-miR-568, cfa-miR-632, bta-miR-376c, mmu-miR-2146, bta-miR-2318, bta-miR-2392, bta-miR-1940, bta-miR-2463, mdo-miR-153, rno-miR-568, rno-miR-632, eca-miR-153, eca-miR-632, eca-miR-568, eca-miR-448
15	mmu-miR-188-3p, hsa-miR-192, hsa-miR-215, hsa-miR-188-3p, mmu-miR-192, rno-miR-192, mmu-miR-215, mmu-miR-412, mml-miR-215, ptr-miR-215, ppy-miR-215, ggo-miR-215, mne-miR-215, hsa-miR-489, hsa-miR-492, hsa-miR-504, rno-miR-215, hsa-miR-591, hsa-miR-642, hsa-miR-660, mmu-miR-693-5p, mmu-miR-702, mmu-miR-711, bta-miR-215, bta-miR-192, bta-miR-425-5p, mdo-miR-10b, bta-miR-660, mmu-miR-874, mmu-miR-504, hsa-miR-874, hsa-miR-938, rno-miR-874, mml-miR-1241, hsa-miR-1291, hsa-miR-1249, hsa-miR-1260, oan-miR-215, oan-miR-192, oan-miR-1391, hsa-miR-1538, mdo-miR-1544, mml-miR-192, mml-miR-489, mml-miR-492, mml-miR-504, mml-miR-642, mml-miR-660, mml-miR-874, mml-miR-938, cfa-miR-1835, cfa-miR-192, cfa-miR-660, ptr-miR-1249, ptr-miR-1291, ptr-miR-192, ptr-miR-489, ptr-miR-492, ptr-miR-504, ptr-miR-591, ptr-miR-642, ptr-miR-660, ptr-miR-874, ptr-miR-938, bta-miR-504, bta-miR-874, mmu-miR-1964, hsa-miR-1979, mmu-miR-1983, cfa-miR-215, cfa-miR-489, cfa-miR-504, cfa-miR-874, bta-miR-489, bta-miR-1291, bta-miR-1835, bta-miR-1249, ssc-miR-215, bta-miR-2314, bta-miR-2363, hsa-miR-711, mdo-miR-215, mml-miR-711, ptr-miR-711, rno-miR-504, rno-miR-711, eca-miR-489, eca-miR-1291a, eca-miR-192, eca-miR-874, eca-miR-711, eca-miR-1291b, eca-miR-220b, eca-miR-412, eca-miR-215, eca-miR-188-3p, eca-miR-504, eca-miR-660
16	mmu-miR-201, hsa-miR-139-5p, rno-miR-335, mmu-miR-139-5p, hsa-miR-335, mmu-miR-335-5p, rno-miR-139-5p, ssc-miR-139, mml-miR-200c, ggo-miR-200c, ppy-miR-200c, mml-miR-30a-3p, ptr-miR-30a-3p, ggo-miR-30a-3p, ppy-miR-30a-3p, ppa-miR-30a-3p, lla-miR-139, ppa-miR-139, hsa-miR-512-5p, hsa-miR-510, mmu-miR-683, bta-miR-139, mmu-miR-743b-5p, mmu-miR-871, rno-miR-871, oan-miR-139, oan-miR-1388, oan-miR-1390, oan-miR-1419g, mml-miR-139-5p, mml-miR-335, mml-miR-512-5p, cfa-miR-335, cfa-miR-30e, mmu-miR-1896, ptr-miR-335, bta-miR-335, bta-miR-2290, bta-miR-2300a-5p, bta-miR-2453, bta-miR-2464-5p, mdo-miR-139, ptr-miR-892b, eca-miR-335, eca-miR-139-5p
17	hsa-miR-216a, mmu-miR-216a, hsa-miR-34b, rno-miR-216a, ssc-miR-216, ggo-miR-29a, age-miR-29a, ppa-miR-29a, ppy-miR-29a, ptr-miR-29a, mml-miR-29a, sla-miR-29a, lla-miR-29a, mne-miR-29a, lca-miR-216, ptr-miR-216a, ggo-miR-216, ppy-miR-216, ppa-miR-216, hsa-miR-518e, mmu-miR-216b, bta-miR-29a, mdo-miR-216, hsa-miR-216b, oan-miR-458, oan-miR-216, mml-miR-34b, mml-miR-216a, mml-miR-216b, mml-miR-518f, cfa-miR-216b, ptr-miR-216b, ptr-miR-34b, ptr-miR-518e, bta-miR-216a, bta-miR-216b, cfa-miR-216a, mdo-miR-875, eca-miR-216a, eca-miR-216b
18	hsa-miR-29a, hsa-miR-29b, mmu-miR-29b, hsa-miR-220a, mmu-miR-29a, mmu-miR-29c, hsa-miR-29c, rno-miR-29b, rno-miR-29a, rno-miR-29c, ssc-miR-29b, ssc-miR-29c, ppy-miR-29b, ptr-miR-29b, ggo-miR-29b, lla-miR-29b, age-miR-29b, ppa-

	miR-29b, sla-miR-29b, mne-miR-29b, ptr-miR-220a, ggo-miR-220, ppa-miR-220, mmu-miR-667, mmu-miR-689, bta-miR-29b, bta-miR-29c, mdo-miR-29b, mdo-miR-29a, hsa-miR-1228, oan-miR-29a, oan-miR-29b, mml-miR-29b, mml-miR-29c, cfa-miR-29b, cfa-miR-29a, cfa-miR-29c, ptr-miR-29c, bta-miR-29d, bta-miR-2350, bta-miR-2456, rno-miR-667, eca-miR-29a, eca-miR-29b, eca-miR-29c
19	mmu-miR-143, hsa-miR-219-1-3p, hsa-miR-143, rno-miR-342-5p, mmu-miR-342-5p, hsa-miR-382, mmu-miR-382, hsa-miR-342-5p, rno-miR-143, rno-miR-219-1-3p, ptr-miR-143, ggo-miR-143, ppy-miR-143, lla-miR-143, ppa-miR-143, rno-miR-382, hsa-miR-571, hsa-miR-608, mdo-miR-143, oan-miR-1331, oan-miR-143, mml-miR-143, mml-miR-342-5p, mml-miR-382, cfa-miR-143, mmu-miR-1902, ptr-miR-219-1-3p, ptr-miR-382, bta-miR-143, bta-miR-382, mmu-miR-2143, bta-miR-2438, ptr-miR-571, eca-miR-1902, eca-miR-143, eca-miR-342-5p, eca-miR-382
20	hsa-miR-26a, hsa-miR-26b, mmu-miR-26a, mmu-miR-26b, rno-miR-26a, rno-miR-26b, ssc-miR-26a, ptr-miR-26a, ggo-miR-26a, ppy-miR-26a, lla-miR-26a, mne-miR-26a, mml-miR-26a, ppa-miR-26a, hsa-miR-573, bta-miR-26a, bta-miR-26b, hsa-miR-1297, oan-miR-26, mml-miR-26b, mml-miR-573, cfa-miR-26a, cfa-miR-26b, ptr-miR-1297, ptr-miR-26b, mdo-miR-26, eca-miR-26a
21	hsa-miR-18a, mmu-miR-18a, rno-miR-18a, hsa-miR-18b, ssc-miR-18, ggo-miR-18, lca-miR-18, age-miR-18, ppa-miR-18, ppy-miR-18, ptr-miR-18a, mml-miR-18, sla-miR-18, lla-miR-18, mne-miR-18, bta-miR-18b, bta-miR-18a, mdo-miR-18, mmu-miR-18b, oan-miR-18, mml-miR-18b, ptr-miR-18b, cfa-miR-18b, cfa-miR-18a, eca-miR-18a, eca-miR-18b
22	hsa-miR-452, hsa-miR-552, oan-miR-1421k, oan-miR-1421r, oan-miR-1421t, oan-miR-1421v, oan-miR-1421aa, oan-miR-1421ad, oan-miR-1421ae, oan-miR-1421af, mml-miR-452, mml-miR-552, ptr-miR-452, ptr-miR-552, cfa-miR-452
23	bta-miR-2471
24	hsa-miR-127-5p, hsa-miR-634, hsa-miR-1301, ptr-miR-634, bta-miR-1301, bta-miR-2284i, eca-miR-1905b, eca-miR-1301
25	mmu-miR-125a-5p, mmu-miR-125b-5p, mmu-miR-202-5p, mmu-miR-204, hsa-miR-204, hsa-miR-211, hsa-miR-125b, hsa-miR-125a-5p, rno-miR-331, mmu-miR-331-3p, rno-miR-339-5p, mmu-miR-339-5p, rno-miR-351, mmu-miR-351, mmu-miR-211, hsa-miR-337-3p, hsa-miR-331-3p, hsa-miR-339-5p, rno-miR-125a-5p, rno-miR-125b-5p, rno-miR-204, rno-miR-211, mmu-miR-433, hsa-miR-433, rno-miR-433, ssc-miR-125b, ssc-miR-204, ggo-miR-125b, age-miR-125b, ppa-miR-125b, ppy-miR-125b, ptr-miR-125b, mml-miR-125b, sla-miR-125b, lla-miR-125b, mne-miR-125b, lca-miR-125b, ptr-miR-204, ggo-miR-204, ppy-miR-204, sla-miR-204, mne-miR-204, ppa-miR-204, mml-miR-211, ppy-miR-211, mne-miR-211, hsa-miR-502-5p, hsa-miR-572, hsa-miR-579, hsa-miR-623, hsa-miR-1468, hsa-miR-670, mmu-miR-670, mmu-miR-712, mmu-miR-715, bta-miR-125a, bta-miR-125b, mdo-miR-125b, mdo-miR-204, bta-miR-204, bta-miR-331, hsa-miR-937, mmu-miR-669j, mmu-miR-669i, hsa-miR-1200, hsa-miR-1274a, hsa-miR-1274b, ssy-miR-513c, oan-miR-204, oan-miR-125, oan-miR-1373, oan-miR-1386, oan-miR-1398, oan-miR-1420g, mml-miR-125a-5p, mml-miR-204, mml-miR-331-3p, mml-miR-337-3p, mml-miR-339-5p, mml-miR-433, mml-miR-502-5p, mml-miR-572, mml-miR-579, cfa-miR-204, cfa-miR-125a, cfa-miR-125b, cfa-miR-433, mmu-miR-1895, ptr-miR-1274b, ptr-miR-211, ptr-miR-331, ptr-miR-337, ptr-miR-433, ptr-miR-502, ptr-miR-572, ptr-miR-579, ptr-miR-937, bta-miR-202, bta-miR-211, bta-miR-339, bta-miR-433, bta-miR-670, mmu-miR-1937b, mmu-miR-1937c, cfa-miR-202, cfa-miR-211, cfa-miR-331, mmu-miR-2133, bta-miR-2326, bta-miR-2328, bta-miR-2367, bta-miR-339b, mml-miR-670, ptr-miR-623, ptr-miR-670, rno-miR-202, eca-miR-204a, eca-miR-211, eca-miR-125b-5p, eca-miR-125a-5p, eca-miR-670, eca-miR-204b, eca-miR-337-3p, eca-miR-433, eca-miR-331, eca-miR-1468, eca-miR-501-5p, eca-miR-502-5p
26	mmu-miR-122, mmu-miR-298, hsa-miR-122, rno-miR-122, rno-miR-298, ssc-miR-122, bta-miR-122, mdo-miR-122, oan-miR-122, oan-miR-1352, mml-miR-122a, cfa-miR-122, ptr-miR-122, bta-miR-2347, bta-miR-2383, bta-miR-2411, bta-miR-2416, bta-miR-2440, eca-miR-122
27	mmu-miR-125a-3p, mmu-miR-126-3p, mmu-miR-127, mmu-miR-128, mmu-miR-9, mmu-miR-149, mmu-miR-187, hsa-miR-139-3p, hsa-miR-187, hsa-miR-219-5p, hsa-miR-128, hsa-miR-9, hsa-miR-125a-3p, hsa-miR-126, hsa-miR-127-3p, hsa-miR-149, mmu-miR-323-5p, rno-miR-327, rno-miR-340-3p, mmu-miR-340-3p, rno-miR-341, mmu-miR-341, rno-miR-345-5p, mmu-miR-345-5p, mmu-miR-219, mmu-miR-125b-3p, hsa-miR-370, hsa-miR-323-5p, rno-miR-9, rno-miR-125a-3p, rno-miR-125b-3p, rno-miR-126, rno-miR-127, rno-miR-128, rno-miR-187, rno-miR-219-5p, mmu-miR-409-5p, mmu-miR-370, hsa-miR-423-5p, oar-miR-127, oar-miR-432, mmu-miR-452, hsa-miR-409-5p, ssc-miR-323, ssc-miR-140, ssc-miR-128, ssc-miR-9-1, ssc-miR-9-2, mml-miR-128a, ptr-miR-128, ppy-miR-128, sla-miR-128, age-miR-128, ppa-miR-128, ptr-miR-140, mne-miR-140, ptr-miR-9, ggo-miR-9, lla-miR-9, mne-miR-9, age-miR-9, mml-miR-127, ptr-miR-127, ppy-miR-127, sla-miR-127, lla-miR-127, mne-miR-127, age-miR-127, ggo-miR-187, ppy-miR-187, mne-miR-187, ppa-miR-187, mml-miR-219, ggo-miR-219, ppy-miR-219, hsa-miR-490-5p, hsa-miR-491-5p, hsa-miR-432, hsa-miR-517b, hsa-miR-516a-3p, hsa-miR-508-3p, hsa-miR-509-3p, rno-miR-370, rno-miR-409-5p, hsa-miR-569, hsa-miR-581, hsa-miR-584, hsa-miR-600, hsa-miR-615-3p, hsa-miR-621, hsa-miR-626, hsa-miR-630, hsa-miR-635, hsa-miR-637, hsa-miR-644, hsa-miR-645, hsa-miR-662, hsa-miR-663, hsa-miR-654-5p, hsa-miR-658, hsa-miR-542-5p, hsa-miR-671-3p, hsa-miR-764, hsa-miR-759, mmu-miR-675-5p, mmu-miR-744, mmu-miR-671-3p, mmu-miR-770-3p, mmu-miR-666-5p, mmu-miR-759, mmu-miR-673-3p, mmu-miR-423-5p, mmu-miR-684, mmu-miR-685, mmu-miR-688, mmu-miR-693-3p, mmu-miR-695, mmu-miR-696, mmu-miR-491, mmu-miR-705, bta-miR-128, mmu-miR-676, mmu-miR-615-3p, bta-miR-127, bta-miR-210, bta-miR-423-5p, bta-miR-23b-5p, mmu-miR-804, mdo-miR-9, mdo-miR-128, mdo-miR-187, mdo-miR-219, mdo-miR-375, hsa-miR-675, mmu-miR-883a-5p, mmu-miR-327, mmu-miR-453, hsa-miR-886-5p, hsa-miR-886-3p, hsa-miR-220c, hsa-miR-541, hsa-miR-876-3p, mmu-miR-466d-5p, hsa-miR-744, hsa-miR-933, rno-miR-466c, mmu-miR-582-3p, rno-miR-598-5p, rno-miR-671, hsa-miR-1181, mmu-miR-669d, mmu-miR-466f, mmu-miR-466k, mmu-miR-1199, hsa-miR-1234, mml-miR-1235, hsa-miR-1237, hsa-miR-663b, hsa-miR-1204, rno-miR-551b, hsa-miR-1293, hsa-miR-1294, hsa-miR-1248, hsa-miR-1250, hsa-miR-1258, hsa-miR-1266, hsa-miR-1267, hsa-miR-1279, hsa-miR-1282, hsa-miR-1308, hsa-miR-1306, age-miR-508, ssy-miR-508, ssy-miR-509a, ssy-miR-509b, mml-miR-508, mml-miR-509, ptr-miR-508, ptr-miR-509a, ptr-miR-509b, pbi-miR-508, hsa-miR-720, oan-miR-9, oan-miR-490, oan-miR-1335, oan-miR-1336, oan-miR-1340, oan-miR-1344, oan-miR-1345, oan-miR-1354, oan-miR-1421e, oan-miR-1421g, oan-miR-128, oan-miR-1374, oan-miR-1375, oan-miR-1419a, oan-miR-1421n, oan-miR-1421q, oan-miR-1421s, oan-miR-1421u, oan-miR-1379, oan-miR-1381, oan-miR-1382, oan-miR-126, oan-miR-1384, oan-miR-1394, oan-miR-1396, oan-miR-1402, oan-miR-1421ab, oan-miR-187, oan-miR-1415, oan-miR-1416, hsa-miR-1469, hsa-miR-1471, hsa-miR-1539, hsa-miR-103-as, mdo-miR-1540, mdo-miR-1545, mml-miR-9, mml-miR-125a-3p, mml-miR-126, mml-miR-128b, mml-miR-139-3p, mml-miR-149, mml-miR-187, mml-miR-219-5p, mml-miR-323-5p, mml-miR-370, mml-miR-409-5p, mml-miR-423-5p, mml-miR-432, mml-miR-490-5p, mml-miR-491-5p, mml-miR-516a-3p, mml-miR-517b, mml-miR-542-5p, mml-miR-567, mml-miR-569, mml-miR-581, mml-miR-584, mml-miR-604, mml-miR-615-3p, mml-miR-626, mml-miR-627, mml-miR-644, mml-miR-654-5p, mml-miR-662, mml-miR-663, mml-miR-671-3p, mml-miR-675, mml-miR-876-3p, mml-miR-886-5p, mml-miR-886-3p, mml-miR-933, cfa-miR-219, cfa-miR-1837, cfa-miR-128, cfa-miR-9, cfa-miR-193b, cfa-miR-127, cfa-miR-212, cfa-miR-423a, mmu-miR-1903, mmu-miR-1899, mmu-miR-1894-3p, mmu-miR-1901, hsa-miR-1908, hsa-miR-1915, ptr-miR-1181, ptr-miR-1204, ptr-

	miR-1234, ptr-miR-1237, ptr-miR-1248, ptr-miR-1250, ptr-miR-1258, ptr-miR-125a, ptr-miR-126, ptr-miR-1266, ptr-miR-1267, ptr-miR-1282, ptr-miR-1293, ptr-miR-1294, ptr-miR-1306, ptr-miR-139, ptr-miR-149, ptr-miR-219-5p, ptr-miR-370, ptr-miR-432, ptr-miR-516a, ptr-miR-569, ptr-miR-581, ptr-miR-584, ptr-miR-600, ptr-miR-615, ptr-miR-621, ptr-miR-626, ptr-miR-630, ptr-miR-635, ptr-miR-637, ptr-miR-645, ptr-miR-658, ptr-miR-663a, ptr-miR-663b, ptr-miR-671, ptr-miR-720, ptr-miR-744, ptr-miR-876, ptr-miR-886, ptr-miR-933, bta-miR-187, bta-miR-212, bta-miR-219-5p, bta-miR-323, bta-miR-340, bta-miR-370, bta-miR-375, bta-miR-409, bta-miR-432, bta-miR-452, bta-miR-491, bta-miR-541, bta-miR-551b, bta-miR-584, bta-miR-658, bta-miR-685, bta-miR-744, bta-miR-9, mmu-miR-1938, mmu-miR-1942, mmu-miR-1306, mmu-miR-1946a, mmu-miR-1946b, hsa-miR-1973, hsa-miR-1978, cfa-miR-187, cfa-miR-210, cfa-miR-302a, cfa-miR-302b, cfa-miR-302d, cfa-miR-367, cfa-miR-149, cfa-miR-370, cfa-miR-432, cfa-miR-671, cfa-miR-759, bta-miR-1282, bta-miR-1248, hsa-miR-2115, hsa-miR-548q, mmu-miR-2132, mmu-miR-2137, mmu-miR-2139, mmu-miR-2142, bta-miR-2288, bta-miR-2289, bta-miR-2295, bta-miR-2299-3p, bta-miR-2305, bta-miR-2307, bta-miR-2309, bta-miR-2321, bta-miR-2324, bta-miR-2329-3p, bta-miR-2329-5p, bta-miR-2331, bta-miR-2333, bta-miR-2335, bta-miR-1814a, bta-miR-2344, bta-miR-2346, bta-miR-2348, bta-miR-2356, bta-miR-1814b, bta-miR-2368, bta-miR-2378, bta-miR-2325c, bta-miR-2386, bta-miR-2388, bta-miR-2396, bta-miR-2405, bta-miR-2413, bta-miR-2430, bta-miR-2432, bta-miR-2437, bta-miR-2445, bta-miR-2447, bta-miR-2455, bta-miR-2460, bta-miR-1777b, bta-miR-2472, bta-miR-2479, mdo-miR-210, mdo-miR-363, mdo-miR-759, mmu-miR-432, mml-miR-1234, mml-miR-759, ptr-miR-759, rno-miR-675, rno-miR-666, rno-miR-759, rno-miR-685, eca-miR-1282, eca-miR-685, eca-miR-492, eca-miR-684, eca-miR-490-5p, eca-miR-671-3p, eca-miR-9a, eca-miR-149, eca-miR-615-3p, eca-miR-139-3p, eca-miR-187, eca-miR-301b-5p, eca-miR-1204, eca-miR-125a-3p, eca-miR-423-5p, eca-miR-675, eca-miR-340-3p, eca-miR-128, eca-miR-1248, eca-miR-219-5p, eca-miR-491-5p, eca-miR-876-3p, eca-miR-127, eca-miR-323-5p, eca-miR-370, eca-miR-409-5p, eca-miR-432, eca-miR-541, eca-miR-126-3p, eca-miR-508-3p
28	hsa-miR-105, ssc-miR-105-1, ssc-miR-105-2, ppy-miR-105, ggo-miR-105, ppa-miR-105, ptr-miR-105, mml-miR-105, sla-miR-105, lla-miR-105, mne-miR-105, mmu-miR-105, bta-miR-105b, bta-miR-105a, cfa-miR-105a, cfa-miR-105b, bta-miR-2475, eca-miR-105
29	mmu-miR-132, mmu-miR-191, hsa-miR-212, hsa-miR-224, hsa-miR-132, hsa-miR-191, mmu-miR-212, mmu-miR-224, rno-miR-132, rno-miR-191, rno-miR-212, ssc-miR-224, mml-miR-224, ptr-miR-224, ggo-miR-224, ppy-miR-224, mne-miR-224, ppa-miR-224, hsa-miR-495, rno-miR-224, hsa-miR-614, hsa-miR-641, mmu-miR-674, mmu-miR-495, bta-miR-132, bta-miR-191, mdo-miR-212, mdo-miR-132, mdo-miR-191, rno-miR-495, rno-miR-674-5p, mmu-miR-1192, hsa-miR-1324, oan-miR-1422e, oan-miR-1422f, oan-miR-1422g, oan-miR-191, mml-miR-132, mml-miR-191, mml-miR-212, mml-miR-495, cfa-miR-191, cfa-miR-495, cfa-miR-132, cfa-miR-224, ptr-miR-1324, ptr-miR-191, ptr-miR-495, ptr-miR-614, ptr-miR-641, bta-miR-224, bta-miR-495, mmu-miR-1936, bta-miR-2284t, bta-miR-2352, bta-miR-2435, bta-miR-1434, bta-miR-2461-5p, bta-miR-2483, eca-miR-132, eca-miR-212, eca-miR-191, eca-miR-495, eca-miR-224
30	oan-miR-1339
31	hsa-miR-31, mmu-miR-203, hsa-miR-203, mmu-miR-31, rno-miR-31, rno-miR-203, rno-miR-300-5p, hsa-miR-488, hsa-miR-498, hsa-miR-518a-5p, hsa-miR-527, hsa-miR-607, mmu-miR-488, mmu-miR-694, bta-miR-31, mdo-miR-203, rno-miR-488, oan-miR-31, mml-miR-203, mml-miR-488, mml-miR-498, cfa-miR-31, mmu-miR-1898, ptr-miR-203, ptr-miR-488, ptr-miR-498, ptr-miR-527, bta-miR-488, mmu-miR-1931, cfa-miR-203, cfa-miR-300, bta-miR-2296, bta-miR-2284q, bta-miR-2457, eca-miR-488, eca-miR-1898, eca-miR-31
32	hsa-miR-451, mmu-miR-451, rno-miR-451, hsa-miR-490-3p, hsa-miR-649, mmu-miR-490, mdo-miR-451, oan-miR-451, mml-miR-451, mml-miR-490-3p, mml-miR-649, ptr-miR-451, ptr-miR-490, ptr-miR-649, bta-miR-451, bta-miR-490, cfa-miR-451, cfa-miR-490, bta-miR-2285d, bta-miR-2285c, rno-miR-490, eca-miR-490-3p, eca-miR-451, eca-miR-582-3p
33	mmu-miR-205, hsa-miR-205, hsa-miR-217, mmu-miR-217, rno-miR-205, rno-miR-217, ssc-miR-217, ssc-miR-205, ggo-miR-205, age-miR-205, ppa-miR-205, ptr-miR-205, lla-miR-205, mne-miR-205, ggo-miR-217, ppa-miR-217, hsa-miR-508-5p, hsa-miR-509-5p, hsa-miR-598, bta-miR-205, mdo-miR-217, mmu-miR-880, mmu-miR-509-5p, hsa-miR-888, mmu-miR-598, hsa-miR-509-3-5p, rno-miR-880, rno-miR-598-3p, age-miR-510, ssy-miR-510, mml-miR-510, pbi-miR-510, oan-miR-205, oan-miR-217, mml-miR-205, mml-miR-598, mml-miR-888, ptr-miR-217, ptr-miR-598, bta-miR-217, mmu-miR-1930, cfa-miR-205, cfa-miR-217, mdo-miR-205a, eca-miR-598, eca-miR-205, eca-miR-217, eca-miR-508-5p, eca-miR-509-5p
34	hsa-miR-583, hsa-miR-1276, mml-miR-583, ptr-miR-1276, ptr-miR-583
35	mmu-miR-185, hsa-miR-7, hsa-miR-185, rno-miR-7a, mmu-miR-139-3p, mmu-miR-7a, mmu-miR-7b, rno-miR-7b, rno-miR-139-3p, rno-miR-185, mmu-miR-434-3p, ssc-miR-7, ggo-miR-7, ppy-miR-7, sla-miR-7, lla-miR-7, mne-miR-7, ppa-miR-7, ptr-miR-7, hsa-miR-580, hsa-miR-638, hsa-miR-650, hsa-miR-671-5p, mmu-miR-671-5p, mmu-miR-764-3p, bta-miR-7, mdo-miR-7, mmu-miR-882, hsa-miR-920, hsa-miR-939, rno-miR-434, hsa-miR-1302, hsa-miR-1273, hsa-miR-1292, oan-miR-1421i, oan-miR-7, oan-miR-1422o, oan-miR-1408, mml-miR-7, mml-miR-185, mml-miR-638, mml-miR-650a, mml-miR-650b, mml-miR-650c, mml-miR-650d, mml-miR-671-5p, mml-miR-920, mml-miR-939, cfa-miR-7, cfa-miR-139, cfa-miR-185, ssc-miR-185, ptr-miR-1273, ptr-miR-1292, ptr-miR-1302, ptr-miR-185, ptr-miR-580, ptr-miR-650, ptr-miR-920, ptr-miR-939, bta-miR-185, bta-miR-671, mmu-miR-1941-5p, mmu-miR-1948, mmu-miR-1953, mmu-miR-1963, bta-miR-2287, bta-miR-2300b-3p, bta-miR-1343, bta-miR-2370, bta-miR-2382, bta-miR-2387, eca-miR-7, eca-miR-1302, eca-miR-1302b, eca-miR-671-5p, eca-miR-1302d, eca-miR-764-3p
36	hsa-miR-196a, mmu-miR-196a, rno-miR-196a, hsa-miR-196b, mmu-miR-196b, rno-miR-196b, ssc-miR-196, mml-miR-196a, ggo-miR-196, ppy-miR-196, ptr-miR-196a, ppy-miR-196-2, lla-miR-196, age-miR-196, ppa-miR-196, mdo-miR-196b, rno-miR-196c, oan-miR-196a, oan-miR-1348, oan-miR-196b, mml-miR-196b, cfa-miR-196b, cfa-miR-196a, cfa-miR-1839, ptr-miR-196b, bta-miR-196a, bta-miR-196b, mmu-miR-1839-5p, bta-miR-1839, eca-miR-1839, eca-miR-196b, eca-miR-196a
37	mmu-miR-154, hsa-miR-154, mmu-miR-380-5p, rno-miR-154, ptr-miR-154, ggo-miR-154, ppy-miR-154, mne-miR-154, ppa-miR-154, hsa-miR-555, hsa-miR-577, hsa-miR-629, mmu-miR-876-5p, hsa-miR-876-5p, mmu-miR-872, rno-miR-872, rno-miR-380, hsa-miR-1290, hsa-miR-1265, hsa-miR-1321, oan-miR-1421a, oan-miR-1421c, oan-miR-1371, oan-miR-1409, oan-miR-1411, oan-miR-1421al, mml-miR-154, mml-miR-577, mml-miR-876-5p, cfa-miR-664, ptr-miR-1265, ptr-miR-1290, ptr-miR-555, ptr-miR-577, bta-miR-154, bta-miR-876, bta-miR-940, mmu-miR-1943, mmu-miR-1958, mmu-miR-1966, mmu-miR-1969, cfa-miR-876, cfa-miR-872, hsa-miR-2053, mmu-miR-2138, bta-miR-2294, bta-miR-2381, bta-miR-2393, bta-miR-2406, bta-miR-2426, bta-miR-2448, bta-miR-2484, bta-miR-664, rno-miR-876, eca-miR-1255b, eca-miR-872, eca-miR-876-5p, eca-miR-154
38	mmu-miR-290-3p, mmu-miR-291a-3p, mmu-miR-292-3p, mmu-miR-294, mmu-miR-295, mmu-miR-302a, hsa-miR-302a, hsa-miR-302b, hsa-miR-302c, hsa-miR-302d, hsa-miR-372, hsa-miR-373, rno-miR-291a-3p, mmu-miR-467a, ggo-miR-93, age-miR-93, ppa-miR-93, ppy-miR-93, ptr-miR-93, mml-miR-93, sla-miR-93, lla-miR-93, mne-miR-93, hsa-miR-520e, hsa-miR-519c-3p, hsa-miR-520a-3p, hsa-miR-519b-3p, hsa-miR-520b, hsa-miR-520c-3p, hsa-miR-520d-3p, hsa-miR-519a, mmu-miR-

	291b-3p, mmu-miR-302b, mmu-miR-302d, mdo-miR-93, mdo-miR-302b, mdo-miR-302c, mdo-miR-302a, mdo-miR-302d, mmu-miR-467c, mmu-miR-467d, hsa-miR-302e, oan-miR-1420a, oan-miR-1420b, oan-miR-1420c, oan-miR-1420e, oan-miR-1347, oan-miR-1420f, oan-miR-1378, oan-miR-302, mml-miR-302a, mml-miR-302b, mml-miR-302c, mml-miR-302d, mml-miR-372, mml-miR-373, mml-miR-519a, mml-miR-519c, mml-miR-520a, mml-miR-520b, mml-miR-520c, mml-miR-520d-3p, mml-miR-520e, cfa-miR-106a, ptr-miR-302a, ptr-miR-302b, ptr-miR-302c, ptr-miR-302d, ptr-miR-302e, ptr-miR-372, ptr-miR-519a, ptr-miR-519b, ptr-miR-519c, ptr-miR-520a, ptr-miR-520b, ptr-miR-520d, ptr-miR-520e, bta-miR-302d, bta-miR-302b, bta-miR-302c, rno-miR-105, eca-miR-302a, eca-miR-302b, eca-miR-302c, eca-miR-302d
39	mmu-miR-194, hsa-miR-194, rno-miR-342-3p, mmu-miR-342-3p, hsa-miR-342-3p, rno-miR-194, mml-miR-194, ptr-miR-194, ppy-miR-194, ggo-miR-194, mne-miR-194, age-miR-194, mmu-miR-542-3p, rno-miR-542-3p, hsa-miR-597, hsa-miR-542-3p, hsa-miR-668, mmu-miR-668, bta-miR-342, oan-miR-194, mml-miR-342-3p, mml-miR-542-3p, mml-miR-597, mml-miR-600, mml-miR-668, cfa-miR-194, cfa-miR-342, cfa-miR-542, ptr-miR-342, ptr-miR-597, ptr-miR-668, bta-miR-194, mmu-miR-1970, cfa-miR-302c, bta-miR-2409, rno-miR-668, eca-miR-194, eca-miR-342-3p, eca-miR-542-3p
40	hsa-miR-30a, mmu-miR-30a, mmu-miR-30b, mmu-miR-142-5p, hsa-miR-30c, hsa-miR-30d, mmu-miR-30e, hsa-miR-30b, hsa-miR-142-5p, mmu-miR-30c, mmu-miR-30d, hsa-miR-30e, rno-miR-30c, rno-miR-30e, rno-miR-30b-5p, rno-miR-30d, rno-miR-30a, rno-miR-142-5p, mmu-miR-384-5p, ssc-miR-30c, mml-miR-30b, ptr-miR-30b, ggo-miR-30b, lla-miR-30b, mne-miR-30b, age-miR-30b, ppa-miR-30b, mml-miR-30a-5p, ptr-miR-30a-5p, ggo-miR-30a-5p, ppy-miR-30a-5p, ppa-miR-30a-5p, ptr-miR-30c, lla-miR-30c, mne-miR-30c, ptr-miR-30d, ggo-miR-30d, mne-miR-30d, ppa-miR-30d, hsa-miR-505, hsa-miR-549, mmu-miR-505, bta-miR-30d, bta-miR-30b-5p, bta-miR-142, bta-miR-30e-5p, bta-miR-30a-5p, bta-miR-30c, mdo-miR-30a, rno-miR-384-5p, oan-miR-30c, oan-miR-30a, oan-miR-1350, oan-miR-142, oan-miR-30f, oan-miR-30d, oan-miR-30b, oan-miR-30e, mml-miR-30c, mml-miR-30d, mml-miR-30e, mml-miR-142-5p, mml-miR-505, cfa-miR-30a, cfa-miR-30c, cfa-miR-30d, cfa-miR-30b, ssc-miR-30b, ptr-miR-30e, ptr-miR-505, ptr-miR-549, bta-miR-30f, bta-miR-505, ssc-miR-30a, eca-miR-30c, eca-miR-30e, eca-miR-30b, eca-miR-30d, eca-miR-142-5p, eca-miR-505
41	mmu-miR-141, hsa-miR-141, hsa-miR-129-3p, mmu-miR-200a, mmu-miR-129-3p, rno-miR-349, hsa-miR-200a, rno-miR-141, rno-miR-200a, mmu-miR-468, hsa-miR-532-5p, mmu-miR-532-5p, hsa-miR-618, hsa-miR-1264, bta-miR-200a, bta-miR-532, mdo-miR-141, mdo-miR-200a, rno-miR-532-5p, mmu-miR-466i, hsa-miR-1269, oan-miR-200a, mml-miR-129-3p, mml-miR-200a, mml-miR-532-5p, mml-miR-616, mml-miR-618, mml-miR-942, cfa-miR-1836, cfa-miR-532, ptr-miR-1264, ptr-miR-141, ptr-miR-200a, ptr-miR-618, bta-miR-129-3p, bta-miR-141, bta-miR-2306, bta-miR-2319a, bta-miR-2357, eca-miR-200a, eca-miR-129a-3p, eca-miR-141, eca-miR-129b-3p, eca-miR-1264, eca-miR-532-5p
42	hsa-miR-33a, mmu-miR-145, hsa-miR-145, mmu-miR-33, rno-miR-33, rno-miR-145, ssc-miR-145, mml-miR-145, ptr-miR-145, ggo-miR-145, ppy-miR-145, mne-miR-145, mml-miR-33a, ptr-miR-33a, ggo-miR-33, ppy-miR-33, mne-miR-33, ppa-miR-33, hsa-miR-520a-5p, hsa-miR-525-5p, hsa-miR-33b, mmu-miR-682, bta-miR-145, mdo-miR-145, mmu-miR-511, hsa-miR-1179, oan-miR-33-5p, oan-miR-145, mml-miR-33b, mml-miR-525, cfa-miR-33, ptr-miR-1179, ptr-miR-33b, bta-miR-24, bta-miR-33a, bta-miR-33b, cfa-miR-33b, cfa-miR-145, bta-miR-1179, bta-miR-2404, bta-miR-2464-3p, mdo-miR-33, mdo-miR-739, rno-miR-511, eca-miR-1179, eca-miR-33b, eca-miR-145, eca-miR-33a
43	mmu-miR-1, mmu-miR-184, mmu-miR-206, hsa-miR-1, hsa-miR-184, hsa-miR-206, rno-miR-184, rno-miR-206, ssc-miR-184, ptr-miR-184, ppy-miR-184, mne-miR-184, ppy-miR-206, mne-miR-206, ppa-miR-1, rno-miR-1, hsa-miR-585, hsa-miR-613, hsa-miR-762, mmu-miR-762, bta-miR-27a-5p, mdo-miR-1, mdo-miR-184, mdo-miR-206, hsa-miR-887, hsa-miR-943, hsa-miR-1268, hsa-miR-1288, oan-miR-1a, oan-miR-1421d, oan-miR-1421f, oan-miR-206, oan-miR-184, oan-miR-1421m, oan-miR-1b, mml-miR-1, mml-miR-184, mml-miR-206, mml-miR-887, cfa-miR-206, cfa-miR-1, cfa-miR-1844, ptr-miR-1, ptr-miR-1288, ptr-miR-206, ptr-miR-613, ptr-miR-887, ptr-miR-943, bta-miR-1, bta-miR-184, bta-miR-206, mmu-miR-1959, cfa-miR-184, ssc-miR-1a, bta-miR-2330, bta-miR-2380, bta-miR-2418, bta-miR-2477, mdo-miR-151, mml-miR-762, ptr-miR-762, bta-miR-2917, eca-miR-184, eca-miR-1, eca-miR-206
44	mmu-miR-181a, hsa-miR-181a, hsa-miR-181b, hsa-miR-181c, mmu-miR-181b, mmu-miR-181c, rno-miR-181c, rno-miR-181a, rno-miR-181b, ssc-miR-181b, ssc-miR-181c, ggo-miR-181a, ppa-miR-181a, ptr-miR-181a, mml-miR-181a, sla-miR-181a, mne-miR-181a, mml-miR-181c, ptr-miR-181c, ggo-miR-181c, ppa-miR-181c, mml-miR-181b, ptr-miR-181b, ppy-miR-181a, ppy-miR-181b, ggo-miR-181b, lla-miR-181a, lla-miR-181b, mne-miR-181b, ppa-miR-181b, hsa-miR-181d, bta-miR-181a, bta-miR-181b, bta-miR-181c, mdo-miR-181c, mdo-miR-181a, mdo-miR-181b, mmu-miR-181d, rno-miR-181d, mmu-miR-1-2-as, oan-miR-181a, oan-miR-181b, oan-miR-181c, mml-miR-181d, cfa-miR-181c, cfa-miR-181d, cfa-miR-181a, cfa-miR-181b, ptr-miR-181d, bta-miR-181d, ssc-miR-181a, eca-miR-181a, eca-miR-181b
45	hsa-miR-24, hsa-miR-28-3p, mmu-miR-24, hsa-miR-221, hsa-miR-222, mmu-miR-221, mmu-miR-222, hsa-miR-299-5p, hsa-miR-379, mmu-miR-379, rno-miR-24, rno-miR-221, rno-miR-222, rno-miR-299, mmu-miR-411, rno-miR-421, hsa-miR-453, mmu-miR-463, mmu-miR-469, ssc-miR-24, mml-miR-24, ppy-miR-24, mne-miR-24, ppa-miR-24, mml-miR-221, ggo-miR-221, ppy-miR-221, ppa-miR-221, age-miR-222, ggo-miR-24, ptr-miR-24, rno-miR-379, hsa-miR-563, hsa-miR-564, hsa-miR-661, hsa-miR-411, mmu-miR-760, bta-miR-221, bta-miR-222, bta-miR-380-5p, bta-miR-423-3p, bta-miR-24-3p, mdo-miR-222a, mdo-miR-221, mdo-miR-24, mmu-miR-509-3p, hsa-miR-760, rno-miR-411, rno-miR-463, rno-miR-760-5p, rno-miR-760-3p, hsa-miR-1201, hsa-miR-1254, age-miR-509a, age-miR-509b, pbi-miR-509, oan-miR-24, oan-miR-222b, oan-miR-222a, oan-miR-221, oan-miR-1392, mml-miR-222, mml-miR-299-5p, mml-miR-379, mml-miR-411, mml-miR-453, mml-miR-563, mml-miR-661, cfa-miR-24, cfa-miR-28, cfa-miR-1842, cfa-miR-379, cfa-miR-221, ssc-miR-221, ptr-miR-1201, ptr-miR-1254, ptr-miR-221, ptr-miR-379, ptr-miR-411, ptr-miR-453, ptr-miR-564, ptr-miR-760, bta-miR-299, bta-miR-379, bta-miR-453, bta-miR-760, mmu-miR-1927, mmu-miR-1928, mmu-miR-1935, hsa-miR-1974, cfa-miR-222, cfa-miR-514, cfa-miR-299, bta-miR-2320, bta-miR-2332, bta-miR-2338, bta-miR-2349, bta-miR-2366, bta-miR-2325b, bta-miR-2420, bta-miR-2428, bta-miR-2436-5p, bta-miR-2284e, mml-miR-760, ptr-miR-299, ptr-miR-661, eca-miR-1905a, eca-miR-24, eca-miR-1842, eca-miR-28-3p, eca-miR-379, eca-miR-411, eca-miR-221, eca-miR-222, eca-miR-421
46	mmu-miR-124, hsa-miR-124, rno-miR-124, ssc-miR-124a, hsa-miR-525-3p, hsa-miR-524-3p, hsa-miR-506, bta-miR-124a, ssy-miR-506, mml-miR-506, ptr-miR-506, pbi-miR-506, oan-miR-124, cfa-miR-124, ptr-miR-524, ptr-miR-525, bta-miR-124b, eca-miR-124
47	mmu-miR-126-5p, mmu-miR-137, mmu-miR-129-5p, hsa-miR-129-5p, mmu-miR-299, hsa-miR-137, rno-miR-325-3p, mmu-miR-325, rno-miR-129, hsa-miR-299-3p, hsa-miR-375, mmu-miR-375, rno-miR-137, hsa-miR-384, mmu-miR-384-3p, hsa-miR-423-3p, oar-miR-431, mmu-miR-431, rno-miR-450a, cfa-miR-450a, hsa-miR-431, rno-miR-431, mmu-miR-465a-5p, mmu-miR-465a-3p, mmu-miR-466a-5p, mmu-miR-470, hsa-miR-491-3p, hsa-miR-511, hsa-miR-526b, hsa-miR-517a, hsa-miR-517c, hsa-miR-455-5p, hsa-miR-544, mmu-miR-546, rno-miR-543, rno-miR-409-3p, hsa-miR-567, hsa-miR-576-3p, hsa-miR-578, hsa-miR-582-5p, hsa-miR-582-3p, hsa-miR-590-3p, hsa-miR-595, hsa-miR-596, hsa-miR-599, hsa-miR-616, hsa-miR-617, hsa-miR-619, hsa-miR-628-5p, hsa-miR-636, hsa-miR-653, hsa-miR-758, mmu-miR-592, mmu-miR-758, mmu-miR-

	764-5p, mmu-miR-678, mmu-miR-423-3p, mmu-miR-679, mmu-miR-686, mmu-miR-719, mmu-miR-691, mmu-miR-692, mmu-miR-669b, mmu-miR-669c, mmu-miR-720, mmu-miR-698, bta-miR-22-5p, bta-miR-455, mmu-miR-805, mdo-miR-129, mdo-miR-367, mmu-miR-883a-3p, mmu-miR-883b-3p, mmu-miR-465b-5p, mmu-miR-465b-3p, mmu-miR-465c-5p, mmu-miR-465c-3p, mmu-miR-466b-5p, mmu-miR-466c-5p, mmu-miR-466e-5p, mmu-miR-466f-5p, mmu-miR-466g, mmu-miR-590-3p, hsa-miR-890, mmu-miR-878-5p, mmu-miR-544, mmu-miR-653, hsa-miR-934, hsa-miR-944, rno-miR-466b, rno-miR-883, rno-miR-375, rno-miR-384-3p, rno-miR-423, rno-miR-455, rno-miR-758, hsa-miR-1180, mmu-miR-1187, mmu-miR-1191, mmu-miR-669e, mmu-miR-1198, hsa-miR-1238, hsa-miR-1206, hsa-miR-1304, hsa-miR-1305, hsa-miR-1272, hsa-miR-302f, hsa-miR-1322, oan-miR-129, oan-miR-137a, oan-miR-137b, oan-miR-1420d, oan-miR-1422l, oan-miR-1357, oan-miR-590, oan-miR-130a, oan-miR-1380, oan-miR-1387, oan-miR-1401, oan-miR-1419c, oan-miR-1419e, oan-miR-1418, mml-miR-129-5p, mml-miR-137, mml-miR-299-3p, mml-miR-375, mml-miR-384, mml-miR-423-3p, mml-miR-431, mml-miR-455-5p, mml-miR-491-3p, mml-miR-511, mml-miR-517a, mml-miR-519b, mml-miR-523c, mml-miR-544, mml-miR-576-3p, mml-miR-578, mml-miR-582-5p, mml-miR-582-3p, mml-miR-590-3p, mml-miR-599, mml-miR-619, mml-miR-625, mml-miR-628-5p, mml-miR-636, mml-miR-653, mml-miR-758, mml-miR-890, mml-miR-934, mml-miR-944, cfa-miR-491, cfa-miR-455, cfa-miR-129, cfa-miR-590, cfa-miR-137, cfa-miR-409, cfa-miR-126, cfa-miR-384, hsa-miR-1826, mmu-miR-1904, ptr-miR-1206, ptr-miR-1272, ptr-miR-129, ptr-miR-1322, ptr-miR-137, ptr-miR-302f, ptr-miR-375, ptr-miR-423, ptr-miR-431, ptr-miR-491, ptr-miR-511, ptr-miR-517a, ptr-miR-526b, ptr-miR-544, ptr-miR-561, ptr-miR-567, ptr-miR-576, ptr-miR-590, ptr-miR-595, ptr-miR-599, ptr-miR-616, ptr-miR-617, ptr-miR-619, ptr-miR-653, ptr-miR-758, ptr-miR-890, ptr-miR-934, ptr-miR-944, bta-miR-129, bta-miR-129-5p, bta-miR-137, bta-miR-367, bta-miR-431, bta-miR-544b, bta-miR-544a, bta-miR-582, bta-miR-592, bta-miR-599, bta-miR-628, bta-miR-653, bta-miR-758, mmu-miR-1932, mmu-miR-1933-5p, mmu-miR-669o, mmu-miR-1965, hsa-miR-1977, cfa-miR-375, cfa-miR-544, cfa-miR-578, cfa-miR-592, cfa-miR-599, cfa-miR-628, cfa-miR-631, cfa-miR-653, cfa-miR-758, cfa-miR-764, bta-miR-764, hsa-miR-2052, hsa-miR-2116, hsa-miR-2117, mmu-miR-2134, bta-miR-2293, bta-miR-2297, bta-miR-2301, bta-miR-2310, bta-miR-1603, bta-miR-2319b, bta-miR-2325a, bta-miR-2337, bta-miR-2340, bta-miR-2345, bta-miR-2353, bta-miR-2361, bta-miR-2365, bta-miR-2390, bta-miR-2395, bta-miR-2399, bta-miR-2401, bta-miR-2408, bta-miR-2414, bta-miR-1814c, bta-miR-2419, bta-miR-2421, bta-miR-2424, bta-miR-2434, bta-miR-2444, bta-miR-2452, bta-miR-2469, bta-miR-2473, bta-miR-2480, bta-miR-2481, bta-miR-2482, bta-miR-2485, bta-miR-2488, mdo-miR-126, mdo-miR-455, mdo-miR-599, mml-miR-129, mml-miR-526b, mml-miR-571, mml-miR-595, mml-miR-764, ptr-miR-596, ptr-miR-764, rno-miR-544, rno-miR-592, rno-miR-628, rno-miR-653, rno-miR-465, rno-miR-764, rno-miR-678, eca-miR-628a, eca-miR-129a-5p, eca-miR-653, eca-miR-137, eca-miR-1180, eca-miR-423-3p, eca-miR-129b-5p, eca-miR-590-3p, eca-miR-582-5p, eca-miR-491-3p, eca-miR-544b, eca-miR-299, eca-miR-431, eca-miR-544, eca-miR-758, eca-miR-126-5p, eca-miR-384, eca-miR-764-5p
48	hsa-miR-147, hsa-miR-210, hsa-miR-218, mmu-miR-210, mmu-miR-218, rno-miR-210, rno-miR-218, ptr-miR-147a, ppy-miR-147, sla-miR-147, mne-miR-147, ppa-miR-147, ggo-miR-218, age-miR-218, ppa-miR-218, lca-miR-218, ppy-miR-218, ptr-miR-218, sla-miR-218, lla-miR-218, mne-miR-218, mml-miR-218, hsa-miR-512-3p, hsa-miR-520f, hsa-miR-592, hsa-miR-648, mmu-miR-302c, bta-miR-218, mdo-miR-218, mmu-miR-147, hsa-miR-147b, rno-miR-147, mmu-miR-1186, hsa-miR-1225-5p, mml-miR-1225-5p, mml-miR-1230, hsa-miR-1262, hsa-miR-1275, oan-miR-218, oan-miR-1346, oan-miR-147, oan-miR-1404, mml-miR-147a, mml-miR-147b, mml-miR-210, mml-miR-512-3p, mml-miR-520f, mml-miR-592, mml-miR-612, mml-miR-648, cfa-miR-218, ssc-miR-210, ptr-miR-1262, ptr-miR-1275, ptr-miR-147b, ptr-miR-210, ptr-miR-512, ptr-miR-520f, ptr-miR-592, bta-miR-147, bta-miR-302a, cfa-miR-147, hsa-miR-2110, mmu-miR-2136, bta-miR-2308, bta-miR-2342, bta-miR-2407, bta-miR-2423, bta-miR-2454, bta-miR-2470, mdo-miR-147b, mmu-miR-599, eca-miR-147b, eca-miR-218, eca-miR-592
49	hsa-miR-96, mmu-miR-182, hsa-miR-182, mmu-miR-96, rno-miR-96, hsa-miR-450a, mmu-miR-450a-5p, mml-miR-182, ppy-miR-182, mml-miR-96, ptr-miR-96, ggo-miR-96, sla-miR-96, mne-miR-96, ppa-miR-96, hsa-miR-507, hsa-miR-557, hsa-miR-1271, mmu-miR-450b-5p, bta-miR-450, mdo-miR-182, mdo-miR-96, hsa-miR-450b-5p, rno-miR-182, age-miR-507, ssy-miR-507, mml-miR-507, ptr-miR-507, pbi-miR-507, oan-miR-96, oan-miR-1355, mml-miR-450a, mml-miR-450b-5p, cfa-miR-1271, cfa-miR-450b, ptr-miR-1271, ptr-miR-182, ptr-miR-450a, ptr-miR-557, bta-miR-182, bta-miR-96, cfa-miR-182, cfa-miR-96, bta-miR-1271, ssc-miR-450, oan-miR-182, eca-miR-182, eca-miR-96, eca-miR-1271, eca-miR-450a, eca-miR-450b-5p, eca-miR-451a, eca-miR-507
50	mmu-miR-10b, hsa-miR-10a, hsa-miR-10b, rno-miR-344-3p, mmu-miR-344, mmu-miR-10a, hsa-miR-361-3p, rno-miR-10a-5p, ggo-miR-10a, ppy-miR-10a, sla-miR-10a, age-miR-10a, ppa-miR-10a, ggo-miR-10b, mne-miR-10b, ppa-miR-10b, hsa-miR-146b-3p, hsa-miR-523, hsa-miR-558, hsa-miR-610, hsa-miR-631, mmu-miR-763, mmu-miR-701, mmu-miR-704, bta-miR-10a, bta-miR-10b, mdo-miR-10a, mmu-miR-1190, mmu-miR-1196, hsa-miR-1207-3p, oan-miR-1361, oan-miR-1362, oan-miR-10a, oan-miR-1385, oan-miR-10b, oan-miR-1413, oan-miR-1419b, mml-miR-10a, mml-miR-10b, mml-miR-146b-3p, mml-miR-361-3p, mml-miR-558, mml-miR-631, cfa-miR-1840, cfa-miR-10, ptr-miR-10a, ptr-miR-10b, ptr-miR-1207, ptr-miR-361, ptr-miR-523, ptr-miR-558, ptr-miR-610, bta-miR-631, bta-miR-763, mmu-miR-1937a, mmu-miR-1839-3p, bta-miR-2323, bta-miR-2402, bta-miR-2465, ptr-miR-631, eca-miR-146b-3p, eca-miR-763, eca-miR-10a, eca-miR-10b, eca-miR-361-3p
51	hsa-miR-214, mmu-miR-214, rno-miR-17-3p, rno-miR-214, ssc-miR-214, ggo-miR-214, age-miR-214, ppa-miR-214, ppy-miR-214, ptr-miR-214, mml-miR-214, sla-miR-214, mne-miR-214, ggo-miR-17-3p, lca-miR-17-3p, age-miR-17-3p, ppa-miR-17-3p, ppy-miR-17-3p, ptr-miR-17-3p, mml-miR-17-3p, sla-miR-17-3p, lla-miR-17-3p, mne-miR-17-3p, hsa-miR-545, rno-miR-20b-3p, hsa-miR-761, mmu-miR-761, bta-miR-17-3p, bta-miR-214, mdo-miR-214, mdo-miR-17-3p, hsa-miR-922, hsa-miR-1184, hsa-miR-1205, oan-miR-214, mml-miR-545, mml-miR-922, cfa-miR-17, ptr-miR-1184, ptr-miR-1205, ptr-miR-545, ptr-miR-922, bta-miR-761, mmu-miR-1954, cfa-miR-214, cfa-miR-761, bta-miR-2313, bta-miR-2334, mdo-miR-761, mml-miR-761, ptr-miR-761, rno-miR-761, eca-miR-761, eca-miR-214
52	hsa-miR-381, mmu-miR-381, mmu-miR-466a-3p, rno-miR-381, mmu-miR-297b-3p, mmu-miR-466b-3p, mmu-miR-466b-3-3p, mmu-miR-466c-3p, mmu-miR-466e-3p, mmu-miR-466f-3p, hsa-miR-300, mmu-miR-466d-3p, mmu-miR-467g, mml-miR-381, ptr-miR-300, ptr-miR-381, bta-miR-381, cfa-miR-381, eca-miR-381
53	mmu-miR-680, hsa-miR-1207-5p, hsa-miR-1256, ptr-miR-1256, bta-miR-1256, eca-miR-1261
54	hsa-miR-609, hsa-miR-625, mmu-miR-466h, mmu-miR-466j, mml-miR-1227, oan-miR-1369, oan-miR-1421p, oan-miR-1412, mml-miR-609, ptr-miR-609, bta-miR-2327, bta-miR-2374, bta-miR-2385-5p, bta-miR-1584, bta-miR-2412, bta-miR-2433, bta-miR-2443, bta-miR-1777a
55	mmu-miR-199a-5p, hsa-miR-199a-5p, hsa-miR-199b-5p, mmu-miR-376a, rno-miR-199a-5p, ggo-miR-199a, ppa-miR-199a, ppy-miR-199a, ptr-miR-199a-5p, mml-miR-199a, sla-miR-199a, lla-miR-199a, mne-miR-199a, rno-miR-376a, mmu-miR-677, bta-miR-199a-5p, bta-miR-199b, mdo-miR-199b, hsa-miR-1203, hsa-miR-1303, oan-miR-1370, mml-miR-199a-5p, mml-miR-607, cfa-miR-140, hsa-miR-1825, ptr-miR-1203, ptr-miR-1303, ptr-miR-1825, ptr-miR-520c, cfa-miR-488, bta-miR-2375, bta-

	miR-677, bta-miR-2459, eca-miR-199a-5p, eca-miR-199b-5p
56	mmu-miR-146a, hsa-miR-146a, rno-miR-146a, hsa-miR-146b-5p, hsa-miR-539, mmu-miR-539, rno-miR-539, hsa-miR-589, hsa-miR-769-5p, mmu-miR-146b, mmu-miR-706, mmu-miR-741, rno-miR-146b, oan-miR-146b, oan-miR-146a, mml-miR-146a, mml-miR-146b-5p, mml-miR-539, mml-miR-589, cfa-miR-146b, cfa-miR-146a, cfa-miR-1841, ptr-miR-146a, ptr-miR-146b, ptr-miR-539, bta-miR-146b, bta-miR-146a, bta-miR-539, bta-miR-769, cfa-miR-539, cfa-miR-589, ssc-miR-146b, mdo-miR-146a, mdo-miR-146b, ptr-miR-589, eca-miR-146b-5p, eca-miR-769-5p, eca-miR-146a, eca-miR-539
57	mmu-miR-152, hsa-miR-148a, hsa-miR-152, mmu-miR-148a, rno-miR-148b-3p, mmu-miR-148b, rno-miR-339-3p, mmu-miR-339-3p, rno-miR-352, hsa-miR-148b, hsa-miR-339-3p, rno-miR-152, ssc-miR-148a, hsa-miR-455-3p, hsa-miR-556-3p, hsa-miR-1296, mmu-miR-802, mmu-miR-455, bta-miR-148a, bta-miR-148b, mdo-miR-152, mmu-miR-873, hsa-miR-873, hsa-miR-921, hsa-miR-935, rno-miR-873, mmu-miR-1195, hsa-miR-1225-3p, hsa-miR-1233, oan-miR-1334, oan-miR-1337, oan-miR-1353, oan-miR-148, oan-miR-802, oan-miR-1419d, mdo-miR-1543, mdo-miR-1546, mml-miR-148a, mml-miR-148b, mml-miR-152, mml-miR-339-3p, mml-miR-455-3p, mml-miR-549, mml-miR-556-3p, cfa-miR-148a, cfa-miR-148b, cfa-miR-411, cfa-miR-152, hsa-miR-1910, hsa-miR-1911, ptr-miR-1225, ptr-miR-1233, ptr-miR-1296, ptr-miR-148a, ptr-miR-148b, ptr-miR-152, ptr-miR-339, ptr-miR-455, ptr-miR-556, ptr-miR-873, ptr-miR-935, bta-miR-152, bta-miR-411, bta-miR-873, bta-miR-935, mmu-miR-1951, mmu-miR-1960, hsa-miR-1972, cfa-miR-545, bta-miR-1296, mmu-miR-2135, bta-miR-2351, bta-miR-2415, bta-miR-2425, bta-miR-2466-3p, bta-miR-2468, mdo-miR-148, mml-miR-1233, rno-miR-802, rno-miR-935, eca-miR-1296, eca-miR-148a, eca-miR-148b-3p, eca-miR-873
58	hsa-miR-22, mmu-miR-22, rno-miR-22, age-miR-22, ppa-miR-22, lca-miR-22, mml-miR-22, ppy-miR-22, ptr-miR-22, sla-miR-22, lla-miR-22, mne-miR-22, bta-miR-22-3p, mdo-miR-22, oan-miR-22, cfa-miR-22, eca-miR-22
59	mmu-miR-136, hsa-miR-136, rno-miR-324-3p, mmu-miR-324-3p, mmu-miR-326, rno-miR-326, rno-miR-330, mmu-miR-330, rno-miR-336, rno-miR-343, hsa-miR-330-5p, hsa-miR-326, rno-miR-136, oar-miR-136, ssc-miR-326, ssc-miR-136, hsa-miR-483-3p, ptr-miR-136, ggo-miR-136, ppy-miR-136, ppa-miR-136, mml-miR-220a, mne-miR-220, rno-miR-483, hsa-miR-587, hsa-miR-606, hsa-miR-766, mmu-miR-718, mmu-miR-220, mmu-miR-343, hsa-miR-220b, hsa-miR-1226, ptr-miR-1226, mml-miR-1226, hsa-miR-1236, mml-miR-1240, hsa-miR-1208, hsa-miR-1251, oan-miR-1393, mml-miR-136, mml-miR-220b, mml-miR-220c, mml-miR-220d, mml-miR-330-5p, mml-miR-587, cfa-miR-1838, cfa-miR-1306, cfa-miR-136, ptr-miR-1208, ptr-miR-1236, ptr-miR-1251, ptr-miR-220b, ptr-miR-326, ptr-miR-517b, ptr-miR-587, ptr-miR-766, bta-miR-136, bta-miR-326, bta-miR-483, bta-miR-502b, mmu-miR-1952, hsa-miR-1975, hsa-miR-1976, mmu-miR-1982.1, mmu-miR-1982.2, cfa-miR-220b, cfa-miR-220a, cfa-miR-330, cfa-miR-326, cfa-miR-483, cfa-miR-718, bta-miR-220d, bta-miR-220e, bta-miR-220c, bta-miR-220b, bta-miR-1251, bta-miR-1306, mmu-miR-2182, bta-miR-2316, bta-miR-2339, bta-miR-2364, bta-miR-2373, bta-miR-2376, hsa-miR-718, ptr-miR-606, ptr-miR-718, rno-miR-220, rno-miR-201, eca-miR-326, eca-miR-330, eca-miR-324-3p, eca-miR-136,
60	rno-miR-325-5p, rno-miR-338, mmu-miR-338-3p, hsa-miR-338-3p, hsa-miR-325, ssc-miR-325, hsa-miR-554, hsa-miR-628-3p, hsa-miR-643, mmu-miR-710, hsa-miR-770-5p, mdo-miR-338, hsa-miR-1247, mml-miR-325, mml-miR-338-3p, mml-miR-554, mml-miR-628-3p, mml-miR-643, mml-miR-770-5p, cfa-miR-338, ptr-miR-1247, ptr-miR-338, ptr-miR-554, ptr-miR-628, ptr-miR-643, ptr-miR-770, bta-miR-338, bta-miR-665, mmu-miR-1934, cfa-miR-325, bta-miR-2291, bta-miR-2436-3p, eca-miR-338-3p
61	rno-miR-148b-5p, bta-miR-2284f, bta-miR-2284o, eca-miR-148b-5p
62	hsa-miR-25, hsa-miR-32, hsa-miR-92a, mmu-miR-92a, mmu-miR-25, mmu-miR-32, hsa-miR-363, mmu-miR-363, hsa-miR-367, rno-miR-25, rno-miR-32, rno-miR-92a, ssc-miR-32, mml-miR-32, ptr-miR-32, ggo-miR-32, ppy-miR-32, sla-miR-32, mne-miR-32, ppa-miR-32, ggo-miR-92, lca-miR-92, age-miR-92, ppa-miR-92, ppy-miR-92, ptr-miR-92, mml-miR-92a, sla-miR-92, lla-miR-92, ggo-miR-25, ppa-miR-25, ppy-miR-25, ptr-miR-25, mml-miR-25, lla-miR-25, mne-miR-25, mne-miR-92, mmu-miR-367, rno-miR-363, hsa-miR-92b, bta-miR-92, bta-miR-25, mdo-miR-32, mdo-miR-92, mdo-miR-25, mmu-miR-92b, rno-miR-92b, oan-miR-92a, oan-miR-363, oan-miR-92b, oan-miR-32, mml-miR-92b, mml-miR-363, mml-miR-367, cfa-miR-32, cfa-miR-92a, cfa-miR-25, cfa-miR-92b, cfa-miR-363, ptr-miR-367, bta-miR-32, bta-miR-92a, bta-miR-92b, oan-miR-92c, eca-miR-367, eca-miR-92b, eca-miR-25, eca-miR-92a, eca-miR-32, eca-miR-363
63	hsa-miR-15a, hsa-miR-16, hsa-miR-28-5p, mmu-miR-15b, mmu-miR-195, hsa-miR-15b, hsa-miR-195, mmu-miR-15a, mmu-miR-16, rno-miR-322, mmu-miR-322, mmu-miR-28, rno-miR-15b, rno-miR-16, rno-miR-28, rno-miR-195, hsa-miR-424, ssc-miR-15b, ssc-miR-28, ggo-miR-15b, age-miR-15b, ppa-miR-15b, ppy-miR-15b, ptr-miR-15b, mml-miR-15b, lla-miR-15b, mne-miR-15b, ggo-miR-195, ppa-miR-195, age-miR-28, mml-miR-28, ptr-miR-28, ggo-miR-28, ppy-miR-28, mne-miR-28, sla-miR-28, lla-miR-28, ppa-miR-28, age-miR-15a, age-miR-16, ggo-miR-15a, ggo-miR-16, mne-miR-15a, mne-miR-16, sla-miR-15a, sla-miR-16, ppa-miR-15a, ppa-miR-16, lca-miR-15a, lca-miR-16, mml-miR-15a, mml-miR-16, ppy-miR-15a, ppy-miR-16, ptr-miR-15a, ptr-miR-16, lla-miR-15a, lla-miR-16, hsa-miR-497, hsa-miR-503, mmu-miR-503, rno-miR-503, hsa-miR-646, rno-miR-497, mmu-miR-497, mmu-miR-708, bta-miR-16b, bta-miR-15b, mdo-miR-15a, mdo-miR-16, bta-miR-15a, bta-miR-195, bta-miR-497, hsa-miR-708, rno-miR-708, oan-miR-15c, oan-miR-16c, oan-miR-15b, oan-miR-16b, oan-miR-15a, oan-miR-16a, oan-miR-1407, mml-miR-195, mml-miR-424, mml-miR-497, mml-miR-503, cfa-miR-708, cfa-miR-15a, cfa-miR-16, cfa-miR-15b, cfa-miR-497, cfa-miR-195, cfa-miR-503, ssc-miR-15a, ssc-miR-16, mmu-miR-1907, ptr-miR-195, ptr-miR-424, ptr-miR-503, ptr-miR-646, ptr-miR-708, bta-miR-16a, bta-miR-28, bta-miR-708, ssc-miR-503, mdo-miR-28, mdo-miR-195, mdo-miR-497, mml-miR-708, ptr-miR-497, eca-miR-15b, eca-miR-16, eca-miR-708, eca-miR-195, eca-miR-497, eca-miR-15a, eca-miR-28-5p, eca-miR-322, eca-miR-424, eca-miR-503
64	hsa-miR-320a, mmu-miR-320, rno-miR-320, hsa-miR-561, hsa-miR-320b, hsa-miR-320c, bta-miR-320, mml-miR-320, cfa-miR-320, hsa-miR-320d, ptr-miR-320a, ptr-miR-320b, ptr-miR-320c, ptr-miR-320d, bta-miR-2284l, bta-miR-2284g, bta-miR-2284u, bta-miR-2284a, bta-miR-2371, bta-miR-2284b
65	hsa-miR-95, hsa-miR-103, hsa-miR-107, hsa-miR-197, mmu-miR-103, mmu-miR-107, rno-miR-103, rno-miR-107, ssc-miR-95, ssc-miR-103, ssc-miR-107, ptr-miR-95, ggo-miR-95, ppy-miR-95, sla-miR-95, lla-miR-95, ppa-miR-95, age-miR-103, ggo-miR-103, ppa-miR-103, ppy-miR-103, ptr-miR-103, mml-miR-103, lla-miR-103, mne-miR-103, mml-miR-107, ptr-miR-107, ggo-miR-107, ppy-miR-107, lla-miR-107, mne-miR-107, ppa-miR-107, ptr-miR-197, ppy-miR-197, mne-miR-197, age-miR-197, ppa-miR-197, hsa-miR-421, rno-miR-505, hsa-miR-767-5p, mmu-miR-770-5p, bta-miR-103, bta-miR-107, bta-miR-545, mdo-miR-103, mdo-miR-107, mmu-miR-197, mmu-miR-421, hsa-miR-298, hsa-miR-891a, rno-miR-770, oan-miR-103, oan-miR-107, oan-miR-1405, mml-miR-95, mml-miR-197, mml-miR-298, mml-miR-421, mml-miR-548d-5p, mml-miR-767-5p, cfa-miR-107, cfa-miR-103, cfa-miR-197, cfa-miR-421, mmu-miR-1906, ptr-miR-298, ptr-miR-421, ptr-miR-891a, bta-miR-197, bta-miR-421, bta-miR-767, bta-miR-95, cfa-miR-95, bta-miR-2315, mmu-miR-767, rno-miR-294, eca-miR-107b, eca-miR-95, eca-miR-197, eca-miR-107a, eca-miR-103, eca-miR-770-5p, eca-miR-767-5p
66	mmu-miR-183, hsa-miR-183, mmu-miR-335-3p, rno-miR-183, mmu-miR-425, hsa-miR-425, ssc-miR-183, mml-miR-183, ptr-

	miR-183, ggo-miR-183, sla-miR-183, mne-miR-183, ppa-miR-183, hsa-miR-514, mmu-miR-489, rno-miR-489, hsa-miR-654-3p, mmu-miR-450b-3p, mdo-miR-137, mdo-miR-183, mmu-miR-654-3p, rno-miR-425, mmu-miR-1193, age-miR-514, ssy-miR-514, mml-miR-514, ptr-miR-514, pbi-miR-514, oan-miR-183, oan-miR-1332, oan-miR-425, oan-miR-1421x, mml-miR-425, mml-miR-654-3p, cfa-miR-183, cfa-miR-425, ptr-miR-425, ptr-miR-654, bta-miR-183, bta-miR-654, bta-miR-1193, bta-miR-2450c, eca-miR-183, eca-miR-493a, eca-miR-1193, eca-miR-514
67	hsa-miR-23a, hsa-miR-27a, mmu-miR-23b, mmu-miR-27b, hsa-miR-23b, hsa-miR-27b, mmu-miR-23a, mmu-miR-27a, rno-miR-329, mmu-miR-329, rno-miR-350, mmu-miR-350, hsa-miR-362-3p, mmu-miR-362-3p, hsa-miR-376a, hsa-miR-377, mmu-miR-377, rno-miR-23a, rno-miR-23b, rno-miR-27b, rno-miR-27a, rno-miR-290, mmu-miR-376b, hsa-miR-329, ssc-miR-23a, ssc-miR-27a, hsa-miR-376b, hsa-miR-485-3p, ptr-miR-23b, ppy-miR-23b, ppa-miR-23b, ggo-miR-23a, ggo-miR-27a, age-miR-23a, age-miR-27a, ppa-miR-23a, ppa-miR-27a, lca-miR-23a, lca-miR-27a, ppy-miR-23a, ppy-miR-27a, ptr-miR-23a, ptr-miR-27a, mml-miR-23a, mml-miR-27a, sla-miR-23a, sla-miR-27a, mne-miR-23a, mne-miR-27a, hsa-miR-513a-5p, rno-miR-377, rno-miR-376b-3p, hsa-miR-603, mmu-miR-673-5p, mmu-miR-717, bta-miR-27a-3p, bta-miR-27b, bta-miR-23a, bta-miR-23b-3p, mdo-miR-23a, mdo-miR-27a, mdo-miR-23b, mdo-miR-27b, rno-miR-673, hsa-miR-1229, age-miR-513b, age-miR-513c, age-miR-513a, age-miR-513d, ssy-miR-513b, ssy-miR-513a, mml-miR-513b, mml-miR-513a, ptr-miR-513a, ptr-miR-513b, pbi-miR-513a, pbi-miR-513b, pbi-miR-513c, hsa-miR-513b, hsa-miR-513c, oan-miR-27b, oan-miR-23b, oan-miR-23a, oan-miR-27a, mml-miR-23b, mml-miR-27b, mml-miR-329, mml-miR-362-3p, mml-miR-376a, mml-miR-376b, mml-miR-377, mml-miR-485-3p, cfa-miR-23b, cfa-miR-27b, cfa-miR-23a, cfa-miR-27a, cfa-miR-350, cfa-miR-376a, ptr-miR-27b, ptr-miR-329, ptr-miR-362, ptr-miR-376a, ptr-miR-376b, ptr-miR-377, ptr-miR-485, bta-miR-329a, bta-miR-362-3p, bta-miR-377, cfa-miR-376b, cfa-miR-329b, bta-miR-376b, bta-miR-376d, bta-miR-376a, mml-miR-603, rno-miR-513, eca-miR-23a, eca-miR-27a, eca-miR-23b, eca-miR-27b, eca-miR-329, eca-miR-376a, eca-miR-376b, eca-miR-377, eca-miR-485-3p, eca-miR-350, eca-miR-362-3p
68	hsa-miR-361-5p, mmu-miR-361, hsa-miR-383, mmu-miR-383, rno-miR-34b, rno-miR-383, rno-miR-361, mmu-miR-540-3p, rno-miR-540, hsa-miR-575, hsa-miR-449c, mmu-miR-714, bta-miR-30b-3p, bta-miR-361, mdo-miR-383, mmu-miR-654-5p, hsa-miR-940, mmu-miR-1194, hsa-miR-1202, hsa-miR-1253, age-miR-513e, oan-miR-1328, oan-miR-1421h, oan-miR-1360, oan-miR-1421o, oan-miR-1376, oan-miR-1377, hsa-miR-1537, mml-miR-361-5p, mml-miR-383, mml-miR-639, mml-miR-940, cfa-miR-383, cfa-miR-361, ptr-miR-1202, ptr-miR-1253, ptr-miR-383, ptr-miR-575, ptr-miR-940, bta-miR-383, bta-miR-562, bta-miR-2286, bta-miR-2292, bta-miR-2322, bta-miR-2336, bta-miR-2360, bta-miR-2427, bta-miR-2442, bta-miR-2449, bta-miR-2450a, bta-miR-2486, bta-miR-2487, eca-miR-1302c, eca-miR-1597, eca-miR-383, eca-miR-361-5p
69	mmu-miR-138, mmu-miR-140, mmu-miR-193, hsa-miR-138, hsa-miR-140-5p, hsa-miR-193a-3p, rno-miR-140, hsa-miR-369-5p, rno-miR-138, rno-miR-193, hsa-miR-493, hsa-miR-193b, hsa-miR-516b, hsa-miR-516a-5p, mmu-miR-369-5p, rno-miR-493, rno-miR-369-5p, hsa-miR-657, bta-miR-193a-3p, bta-miR-138, bta-miR-425-3p, mdo-miR-138, mdo-miR-193, mdo-miR-425, mmu-miR-876-3p, mmu-miR-193b, mmu-miR-493, hsa-miR-892b, hsa-miR-1286, hsa-miR-1287, hsa-miR-1299, hsa-miR-1243, hsa-miR-1252, oan-miR-1325, oan-miR-193, oan-miR-1338, oan-miR-1342, oan-miR-1365, oan-miR-1367, oan-miR-1421l, oan-miR-138, oan-miR-1421z, oan-miR-873, oan-miR-1421ai, oan-miR-1421am, mml-miR-138, mml-miR-140-5p, mml-miR-193a-3p, mml-miR-193b, mml-miR-369-5p, mml-miR-493, mml-miR-516a-5p, mml-miR-657, cfa-miR-138b, cfa-miR-138a, cfa-miR-493, ptr-miR-1286, ptr-miR-1299, ptr-miR-138, ptr-miR-193a, ptr-miR-193b, ptr-miR-516b, ptr-miR-657, bta-miR-193b, bta-miR-493, mmu-miR-1947, mmu-miR-1957, bta-miR-1287, mmu-miR-2141, bta-miR-2403, bta-miR-2417, bta-miR-2451, mdo-miR-140, mml-miR-516, mml-miR-892b, eca-miR-1244, eca-miR-138, eca-miR-140-5p, eca-miR-193a-3p, eca-miR-193b, eca-miR-369-5p, eca-miR-493b
70	mmu-miR-199a-3p, hsa-miR-199a-3p, hsa-miR-199b-3p, mmu-miR-199b, hsa-miR-380, mmu-miR-380-3p, rno-miR-199a-3p, mmu-miR-471, hsa-miR-562, bta-miR-199a-3p, bta-miR-380-3p, hsa-miR-936, rno-miR-471, hsa-miR-1277, oan-miR-199, mml-miR-199a-3p, mml-miR-380, mml-miR-562, mml-miR-936, cfa-miR-199, cfa-miR-1843, cfa-miR-380, ptr-miR-199a-3p, ptr-miR-199b, ptr-miR-380, ptr-miR-562, ptr-miR-936, eca-miR-199a-3p, eca-miR-380, eca-miR-199b-3p
71	mmu-miR-134, mmu-miR-151-3p, hsa-miR-134, hsa-miR-193a-5p, rno-miR-344-5p, hsa-miR-151-3p, hsa-miR-345, rno-miR-134, hsa-miR-484, hsa-miR-485-5p, ggo-miR-134, ppy-miR-134, mne-miR-134, ppa-miR-134, hsa-miR-496, mmu-miR-484, mmu-miR-485, rno-miR-485, hsa-miR-574-5p, hsa-miR-593, hsa-miR-622, hsa-miR-1185, mmu-miR-496, bta-miR-151, bta-miR-484, bta-miR-193a-5p, bta-miR-345-5p, bta-miR-365-5p, mmu-miR-879, mmu-miR-574-5p, hsa-miR-924, rno-miR-879, rno-miR-484, hsa-miR-1257, mml-miR-134, mml-miR-151-3p, mml-miR-193a-5p, mml-miR-345, mml-miR-484, mml-miR-485-5p, mml-miR-496, mml-miR-593, mml-miR-924, cfa-miR-485, cfa-miR-193a, ptr-miR-1185, ptr-miR-134, ptr-miR-151, ptr-miR-345, ptr-miR-484, ptr-miR-496, ptr-miR-593, ptr-miR-622, ptr-miR-924, bta-miR-134, bta-miR-485, bta-miR-496, bta-miR-759, mmu-miR-1962, mmu-miR-1967, cfa-miR-134, cfa-miR-496, bta-miR-1185, bta-miR-2461-3p, mml-miR-622, rno-miR-496, eca-miR-193a-5p, eca-miR-1185, eca-miR-134, eca-miR-345-5p, eca-miR-485-5p, eca-miR-496
72	mmu-miR-207, mmu-miR-337-5p, mmu-miR-345-3p, hsa-miR-378, mmu-miR-378, hsa-miR-337-5p, hsa-miR-324-3p, hsa-miR-422a, mmu-miR-434-5p, hsa-miR-412, hsa-miR-501-3p, hsa-miR-502-3p, rno-miR-207, rno-miR-412, hsa-miR-556-5p, hsa-miR-548b-3p, hsa-miR-612, rno-miR-378, hsa-miR-767-3p, mmu-miR-500, mmu-miR-501-3p, bta-miR-345-3p, mmu-miR-878-3p, hsa-miR-942, rno-miR-878, rno-miR-500, mmu-miR-669f, hsa-miR-1285, hsa-miR-1284, hsa-miR-1307, oan-miR-1349, oan-miR-1389, mml-miR-324-3p, mml-miR-337-5p, mml-miR-378, mml-miR-412, mml-miR-422a, mml-miR-502-3p, mml-miR-523a, mml-miR-556-5p, mml-miR-767-3p, cfa-miR-1307, cfa-miR-378, cfa-miR-345, cfa-miR-502, mmu-miR-1897-3p, hsa-miR-1913, ptr-miR-1284, ptr-miR-1285, ptr-miR-1307, ptr-miR-324, ptr-miR-378, ptr-miR-412, ptr-miR-422a, ptr-miR-501, ptr-miR-548b, ptr-miR-612, ptr-miR-942, bta-miR-378, bta-miR-412, bta-miR-502a, mmu-miR-1933-3p, mmu-miR-1945, mmu-miR-1274a, cfa-miR-207, bta-miR-1284, bta-miR-220a, bta-miR-1307, bta-miR-2377, bta-miR-2400, bta-miR-2441, eca-miR-378, eca-miR-337-5p, eca-miR-345-3p, eca-miR-502-3p, eca-miR-767-3p
73	mmu-miR-296-5p, rno-miR-328, mmu-miR-328, hsa-miR-296-5p, hsa-miR-328, ggo-miR-133a, age-miR-133a, ppa-miR-133a, sla-miR-133a, lla-miR-133a, mne-miR-133a, hsa-miR-521, hsa-miR-588, hsa-miR-640, bta-miR-369-5p, bta-miR-363, mdo-miR-133a, mml-miR-1232, hsa-miR-1263, oan-miR-133a, oan-miR-1383, oan-miR-1421ak, mml-miR-296-5p, mml-miR-521, mml-miR-640, cfa-miR-328, ptr-miR-1263, ptr-miR-328, ptr-miR-521, ptr-miR-588, ptr-miR-640, bta-miR-328, cfa-miR-133c, cfa-miR-133a, ssc-miR-133a, bta-miR-2355, bta-miR-2284r, rno-miR-547, eca-miR-328
74	hsa-miR-101, mmu-miR-101a, mmu-miR-144, hsa-miR-144, rno-miR-101b, mmu-miR-101b, rno-miR-101a, rno-miR-144, hsa-miR-486-3p, ptr-miR-144, ppy-miR-144, mne-miR-144, ppa-miR-144, ggo-miR-101, sla-miR-101, age-miR-101, ppa-miR-101, ppy-miR-101, ptr-miR-101, mml-miR-101, lla-miR-101, mne-miR-101, hsa-miR-559, hsa-miR-548b-5p, hsa-miR-548a-5p, hsa-miR-548c-5p, hsa-miR-548d-5p, mmu-miR-709, bta-miR-101, mdo-miR-31, mdo-miR-101, mdo-miR-144, hsa-miR-548j, hsa-miR-548k, hsa-miR-548l, hsa-miR-548h, hsa-miR-548i, oan-miR-144, oan-miR-101, mml-miR-144, mml-miR-486-3p, cfa-miR-101, cfa-miR-144, hsa-miR-1827, ptr-miR-1827, ptr-miR-548h, ptr-miR-548i, ptr-miR-548j, ptr-miR-548k, ptr-miR-548l,

	ptr-miR-559, bta-miR-144, ssc-miR-101a, bta-miR-199c, eca-miR-101, eca-miR-144, eca-miR-486-3p
75	rno-miR-340-5p, mmu-miR-340-5p, hsa-miR-340, hsa-miR-330-3p, ggo-miR-124a, age-miR-124a, ppa-miR-124a, ppy-miR-124a, ptr-miR-124a, mml-miR-124a, lla-miR-124a, hsa-miR-494, hsa-miR-524-5p, hsa-miR-520d-5p, hsa-miR-520g, hsa-miR-520h, mmu-miR-494, rno-miR-494, hsa-miR-570, hsa-miR-548c-3p, hsa-miR-548d-3p, hsa-miR-655, hsa-miR-1323, mdo-miR-124a, hsa-miR-889, hsa-miR-548n, hsa-miR-548o, oan-miR-1333, oan-miR-1422b, oan-miR-1422c, oan-miR-1422k-3p, oan-miR-1422d, mml-miR-330-3p, mml-miR-340, mml-miR-494, mml-miR-518a-5p, mml-miR-520d-5p, mml-miR-520g, mml-miR-520h, mml-miR-548b, mml-miR-548c, mml-miR-548d-3p, mml-miR-548e, mml-miR-548f, mml-miR-570, mml-miR-889, cfa-miR-424, ptr-miR-1323, ptr-miR-330, ptr-miR-340, ptr-miR-494, ptr-miR-520g, ptr-miR-520h, ptr-miR-548c, ptr-miR-548n, ptr-miR-889, bta-miR-330, bta-miR-494, bta-miR-655, cfa-miR-340, cfa-miR-494, bta-miR-2284j, bta-miR-2284d, bta-miR-2284n, bta-miR-2284p, bta-miR-2358, bta-miR-2284k, bta-miR-2284c, bta-miR-2284v, bta-miR-2391, bta-miR-2284m, bta-miR-2446, mml-miR-524, ptr-miR-570, eca-miR-769b, eca-miR-340-5p, eca-miR-494, eca-miR-655, eca-miR-889
76	rno-miR-337, mmu-miR-337-3p, hsa-miR-518f, hsa-miR-518b, hsa-miR-518c, hsa-miR-518a-3p, hsa-miR-518d-3p, hsa-miR-513a-3p, hsa-miR-605, mmu-miR-742, mmu-miR-582-5p, rno-miR-674-3p, rno-miR-742, hsa-miR-548m, oan-miR-1329, oan-miR-1422h, oan-miR-1358, mml-miR-518b, mml-miR-518c, mml-miR-518d, mml-miR-605, ptr-miR-518a, ptr-miR-518b, ptr-miR-518c, ptr-miR-518d, ptr-miR-518f, ptr-miR-582, ptr-miR-605, mmu-miR-1968, cfa-miR-582, bta-miR-2298, bta-miR-2285b, bta-miR-1721, rno-miR-582
77	mmu-miR-186, hsa-miR-208a, hsa-miR-186, mmu-miR-208a, rno-miR-186, rno-miR-208, ssc-miR-186, ptr-miR-186, ggo-miR-186, ppa-miR-186, hsa-miR-499-5p, rno-miR-499, mmu-miR-499, bta-miR-499, bta-miR-186, mmu-miR-743a, mdo-miR-186, mdo-miR-208, mmu-miR-743b-3p, mmu-miR-208b, hsa-miR-208b, rno-miR-743b, rno-miR-743a, oan-miR-208, oan-miR-186, oan-miR-499, mml-miR-186, mml-miR-208a, mml-miR-208b, mml-miR-499-5p, mml-miR-651, cfa-miR-499, cfa-miR-186, ptr-miR-208a, ptr-miR-208b, bta-miR-208a, bta-miR-208b, cfa-miR-208a, cfa-miR-208b, mdo-miR-499, eca-miR-208a, eca-miR-208b, eca-miR-186, eca-miR-499-5p
78	hsa-miR-140-3p, rno-miR-324-5p, mmu-miR-324-5p, hsa-miR-371-3p, hsa-miR-324-5p, rno-miR-292-3p, ssc-miR-301, ppy-miR-133a, mml-miR-133a, hsa-miR-515-5p, hsa-miR-515-3p, hsa-miR-519e, hsa-miR-499-3p, hsa-miR-551a, hsa-miR-566, hsa-miR-551b, hsa-miR-601, hsa-miR-627, hsa-miR-1224-3p, hsa-miR-1298, mmu-miR-551b, mmu-miR-666-3p, mmu-miR-681, mmu-miR-697, bta-miR-140, hsa-miR-891b, hsa-miR-1178, mmu-miR-467f, ptr-miR-1224-3p, mml-miR-1225-3p, mml-miR-1239, hsa-miR-1281, hsa-miR-1280, oan-miR-140, oan-miR-1422k-5p, oan-miR-1351, oan-miR-1421b, oan-miR-1359, oan-miR-1364, oan-miR-1366, oan-miR-1372, oan-miR-551, oan-miR-383, oan-miR-1403, oan-miR-1422q, hsa-miR-1470, mdo-miR-1548, mdo-miR-1549, mml-miR-140-3p, mml-miR-217, mml-miR-324-5p, mml-miR-371-3p, mml-miR-499-3p, mml-miR-551a, mml-miR-551b, mml-miR-557, mml-miR-580, mml-miR-601, mml-miR-891, mml-miR-937, cfa-miR-500, cfa-miR-676, mmu-miR-1900, mmu-miR-1894-5p, ptr-miR-1178, ptr-miR-1280, ptr-miR-1281, ptr-miR-1298, ptr-miR-371, ptr-miR-499, ptr-miR-515, ptr-miR-519e, ptr-miR-551a, ptr-miR-551b, ptr-miR-566, ptr-miR-601, ptr-miR-627, ptr-miR-891b, bta-miR-324, bta-miR-292, bta-miR-551a, mmu-miR-1939, mmu-miR-669m, cfa-miR-324, cfa-miR-505, cfa-miR-551a, cfa-miR-551b, bta-miR-1225-3p, bta-miR-29e, bta-miR-1281, bta-miR-1298, hsa-miR-2114, mmu-miR-2140, mmu-miR-2144, mmu-miR-2183, hsa-miR-2278, bta-miR-2284s, bta-miR-2394, bta-miR-2397, bta-miR-2410, bta-miR-1843, bta-miR-2431, bta-miR-2466-5p, mdo-miR-551a, mdo-miR-551b, eca-miR-551a, eca-miR-140-3p, eca-miR-1905c, eca-miR-371-3p, eca-miR-324-5p, eca-miR-1461, eca-miR-551b, eca-miR-499-3p, eca-miR-1298
79	mmu-miR-1197, hsa-miR-1197, ptr-miR-1197, bta-miR-1197, eca-miR-1197
80	mmu-miR-130a, mmu-miR-301a, mmu-miR-130b, hsa-miR-130a, rno-miR-301a, hsa-miR-301a, hsa-miR-130b, rno-miR-130a, rno-miR-130b, mml-miR-130a, ggo-miR-130a, mne-miR-130a, ppa-miR-130a, hsa-miR-454, mmu-miR-301b, mmu-miR-721, mdo-miR-130a, hsa-miR-301b, rno-miR-301b, oan-miR-130c, oan-miR-301, oan-miR-454, oan-miR-130b, mml-miR-130b, mml-miR-301a, mml-miR-301b, mml-miR-454, cfa-miR-130a, cfa-miR-130b, ssc-miR-130a, ptr-miR-130a, ptr-miR-130b, ptr-miR-301a, ptr-miR-301b, ptr-miR-454, bta-miR-130a, bta-miR-130b, bta-miR-301a, bta-miR-301b, bta-miR-454, cfa-miR-301a, cfa-miR-301b, cfa-miR-454, mdo-miR-301, eca-miR-130b, eca-miR-301b-3p, eca-miR-301a, eca-miR-454, eca-miR-130a
81	mmu-miR-133a, mmu-miR-135a, hsa-miR-133a, hsa-miR-135a, mmu-miR-331-5p, rno-miR-135b, mmu-miR-135b, hsa-miR-135b, hsa-miR-331-5p, mmu-miR-133b, hsa-miR-133b, rno-miR-30b-3p, rno-miR-133a, rno-miR-135a, ssc-miR-135, ptr-miR-133a, lla-miR-135, age-miR-135, ppa-miR-135, mml-miR-135a, ptr-miR-135a, ggo-miR-135, ppy-miR-135, rno-miR-133b, mmu-miR-541, mmu-miR-547, rno-miR-541, hsa-miR-652, hsa-miR-659, hsa-miR-769-3p, mmu-miR-690, mmu-miR-652, mdo-miR-135a, mdo-miR-135b, hsa-miR-450b-3p, rno-miR-652, hsa-miR-1295, hsa-miR-1246, age-miR-506, oan-miR-135b, oan-miR-133b, oan-miR-1363, oan-miR-133c, mdo-miR-1542, mml-miR-133c, mml-miR-133b, mml-miR-135b, mml-miR-331-5p, mml-miR-450b-3p, mml-miR-652, cfa-miR-135a-5p, cfa-miR-652, ptr-miR-1246, ptr-miR-1295, ptr-miR-133b, ptr-miR-135b, ptr-miR-450b, ptr-miR-652, bta-miR-133a, bta-miR-133b, bta-miR-135a, bta-miR-135b, cfa-miR-133b, cfa-miR-135b, bta-miR-2369, bta-miR-449d, bta-miR-2450b, bta-miR-2458, bta-miR-2462, bta-miR-2467, mml-miR-541, eca-miR-135b, eca-miR-133a, eca-miR-1302e, eca-miR-769-3p, eca-miR-135a, eca-miR-133b, eca-miR-450b-3p, eca-miR-652
