# Supplementary material for: Construction of microRNA functional families by a mixture model of position weight matrices
Source: PeerJ. 2013 Oct 31;1:e199. doi: 10.7717/peerj.199 (PMC3817585; doi:10.7717/peerj.199)
Supplement: Table S2 [file peerj-01-199-s003.pdf]

Supplemental Table S2. GO (Molecular Function) enrichment analyses for target genes in each group

group ID	GO Term (Molecular Function)	p-value
c1	GO:0005515~protein binding	0.019583005
c2	GO:0019901~protein kinase binding	0.092968358
c2	GO:0022892~substrate-specific transporter activity	0.013635889
c2	GO:0005515~protein binding	0.017444065
c2	GO:0005215~transporter activity	0.030193237
c2	GO:0005488~binding	0.031758549
c2	GO:0022891~substrate-specific transmembrane transporter activity	0.047020437
c2	GO:0008199~ferric iron binding	0.072803406
c2	GO:0022857~transmembrane transporter activity	0.07908019
c2	GO:0031420~alkali metal ion binding	0.093441631
c2	GO:0000155~two-component sensor activity	0.093601602
c2	GO:0004673~protein histidine kinase activity	0.093601602
c2	GO:0015075~ion transmembrane transporter activity	0.096097271
c11	GO:0004672~protein kinase activity	0.007901069
c11	GO:0005102~receptor binding	0.008857705
c11	GO:0016772~transferase activity, transferring phosphorus-containing groups	0.015388921
c11	GO:0004674~protein serine/threonine kinase activity	0.016356141
c11	GO:0016301~kinase activity	0.016935202
c11	GO:0016773~phosphotransferase activity, alcohol group as acceptor	0.020744203
c11	GO:0005515~protein binding	0.032572584
c11	GO:0000166~nucleotide binding	0.059802919
c11	GO:0005125~cytokine activity	0.065778287
c11	GO:0016740~transferase activity	0.067733775
c11	GO:0005509~calcium ion binding	0.069475014
c11	GO:0003713~transcription coactivator activity	0.0817897
c11	GO:0003712~transcription cofactor activity	0.094265717
c12	GO:0016787~hydrolase activity	1.03E-04
c12	GO:0003824~catalytic activity	5.82E-04
c12	GO:0004518~nuclease activity	0.003167878
c12	GO:0004519~endonuclease activity	0.010980107
c12	GO:0016788~hydrolase activity, acting on ester bonds	0.012740305
c12	GO:0016810~hydrolase activity, acting on carbon-nitrogen (but not peptide) bonds	0.023685152
c12	GO:0016811~hydrolase activity, acting on carbon-nitrogen (but not peptide) bonds, in linear amides	0.030813517
c12	GO:0016407~acetyltransferase activity	0.038089976
c12	GO:0003723~RNA binding	0.047071899
c12	GO:0008080~N-acetyltransferase activity	0.057960941
c12	GO:0004348~glucosylceramidase activity	0.059705252
c12	GO:0042625~ATPase activity, coupled to transmembrane movement of ions	0.073709579
c12	GO:0015405~P-P-bond-hydrolysis-driven transmembrane transporter activity	0.077689438
c12	GO:0015399~primary active transmembrane transporter activity	0.077689438
c12	GO:0019904~protein domain specific binding	0.078973711
c12	GO:0016893~endonuclease activity, active with either ribo- or deoxyribonucleic acids and producing 5'-phosphomonoesters	0.082935812

c12	GO:0030528~transcription regulator activity	0.087772525
c12	GO:0004995~tachykinin receptor activity	0.088210324
c13	GO:0000166~nucleotide binding	0.022249799
c13	GO:0017111~nucleoside-triphosphatase activity	0.038806473
c13	GO:0016462~pyrophosphatase activity	0.042827466
c13	GO:0016818~hydrolase activity, acting on acid anhydrides, in phosphorus-containing anhydrides	0.043255351
c13	GO:0016817~hydrolase activity, acting on acid anhydrides	0.04382933
c13	GO:0003824~catalytic activity	0.059606976
c14	GO:0005544~calcium-dependent phospholipid binding	0.069722617
c16	GO:0004518~nuclease activity	0.023710526
c16	GO:0016891~endoribonuclease activity, producing 5'-phosphomonoesters	0.040243356
c16	GO:0016893~endonuclease activity, active with either ribo- or deoxyribonucleic acids and producing 5'-phosphomonoesters	0.050421158
c16	GO:0004521~endoribonuclease activity	0.069049526
c16	GO:0005515~protein binding	0.094906466
c16	GO:0004540~ribonuclease activity	0.095648023
c17	GO:0004386~helicase activity	0.02215517
c18	GO:0008307~structural constituent of muscle	0.006407843
c18	GO:0030528~transcription regulator activity	0.006681037
c18	GO:0003700~transcription factor activity	0.007199568
c18	GO:0008134~transcription factor binding	0.010009477
c18	GO:0003777~microtubule motor activity	0.012235438
c18	GO:0008168~methyltransferase activity	0.020382095
c18	GO:0016741~transferase activity, transferring one-carbon groups	0.023161211
c18	GO:0003824~catalytic activity	0.024471288
c18	GO:0016706~oxidoreductase activity, acting on paired donors, with incorporation or reduction of molecular oxygen, 2-oxoglutarate as one donor, and incorporation of one atom each of oxygen into both donors	0.024899749
c18	GO:0005516~calmodulin binding	0.026329433
c18	GO:0030371~translation repressor activity	0.029825078
c18	GO:0004177~aminopeptidase activity	0.031297087
c18	GO:0008237~metallopeptidase activity	0.03446524
c18	GO:0005515~protein binding	0.037257822
c18	GO:0003712~transcription cofactor activity	0.038050601
c18	GO:0051213~dioxygenase activity	0.049683427
c18	GO:0016702~oxidoreductase activity, acting on single donors with incorporation of molecular oxygen, incorporation of two atoms of oxygen	0.049683427
c18	GO:0005488~binding	0.04973948
c18	GO:0016740~transferase activity	0.05051883
c18	GO:0016701~oxidoreductase activity, acting on single donors with incorporation of molecular oxygen	0.052796065
c18	GO:0004745~retinol dehydrogenase activity	0.059006933
c18	GO:0004222~metalloendopeptidase activity	0.059100678
c18	GO:0003774~motor activity	0.061665664
c18	GO:0048407~platelet-derived growth factor binding	0.070239269
c18	GO:0019239~deaminase activity	0.077904003
c18	GO:0033612~receptor serine/threonine kinase binding	0.079071893
c18	GO:0004084~branched-chain-amino-acid transaminase activity	0.079071893

c18	GO:0030350~iron-responsive element binding	0.079071893
c18	GO:0001872~zymosan binding	0.079071893
c18	GO:0003682~chromatin binding	0.08225264
c18	GO:0070011~peptidase activity, acting on L-amino acid peptides	0.085179686
c18	GO:0008233~peptidase activity	0.089449942
c20	GO:0016709~oxidoreductase activity, acting on paired donors, with incorporation or reduction of molecular oxygen, NADH or NADPH as one donor, and incorporation of one atom of oxygen	0.001316178
c20	GO:0042626~ATPase activity, coupled to transmembrane movement of substances	0.001604839
c20	GO:0043492~ATPase activity, coupled to movement of substances	0.00171927
c20	GO:0016820~hydrolase activity, acting on acid anhydrides, catalyzing transmembrane movement of substances	0.001840217
c20	GO:0015399~primary active transmembrane transporter activity	0.003681465
c20	GO:0015405~P-P-bond-hydrolysis-driven transmembrane transporter activity	0.003681465
c20	GO:0004842~ubiquitin-protein ligase activity	0.004772958
c20	GO:0005488~binding	0.007327243
c20	GO:0016881~acid-amino acid ligase activity	0.007710907
c20	GO:0004518~nuclease activity	0.008111506
c20	GO:0042623~ATPase activity, coupled	0.008139137
c20	GO:0016879~ligase activity, forming carbon-nitrogen bonds	0.010049033
c20	GO:0019787~small conjugating protein ligase activity	0.011510546
c20	GO:0016887~ATPase activity	0.012241963
c20	GO:0003723~RNA binding	0.016484956
c20	GO:0004536~deoxyribonuclease activity	0.018162807
c20	GO:0019899~enzyme binding	0.025908109
c20	GO:0016874~ligase activity	0.026657696
c20	GO:0008134~transcription factor binding	0.036228584
c20	GO:0003824~catalytic activity	0.038973632
c20	GO:0043130~ubiquitin binding	0.045978098
c20	GO:0032182~small conjugating protein binding	0.050388155
c20	GO:0008060~ARF GTPase activator activity	0.054995739
c20	GO:0004145~diamine N-acetyltransferase activity	0.059064768
c20	GO:0016251~general RNA polymerase II transcription factor activity	0.062332635
c20	GO:0008017~microtubule binding	0.064705492
c20	GO:0043566~structure-specific DNA binding	0.07054392
c20	GO:0042625~ATPase activity, coupled to transmembrane movement of ions	0.071055602
c20	GO:0004497~monooxygenase activity	0.071291266
c20	GO:0004527~exonuclease activity	0.07401208
c20	GO:0005515~protein binding	0.075107661
c20	GO:0003712~transcription cofactor activity	0.081325002
c20	GO:0050661~NADP or NADPH binding	0.092418449
c21	GO:0003824~catalytic activity	0.010478807
c21	GO:0032559~adenyl ribonucleotide binding	0.011688273
c21	GO:0005524~ATP binding	0.013657182
c21	GO:0030554~adenyl nucleotide binding	0.013789515
c21	GO:0001882~nucleoside binding	0.014157796
c21	GO:0016887~ATPase activity	0.016733843

c21	GO:0001883~purine nucleoside binding	0.018305126
c21	GO:0032553~ribonucleotide binding	0.025611366
c21	GO:0032555~purine ribonucleotide binding	0.025611366
c21	GO:0042623~ATPase activity, coupled	0.02955997
c21	GO:0017076~purine nucleotide binding	0.029596033
c21	GO:0000166~nucleotide binding	0.045600443
c21	GO:0016787~hydrolase activity	0.046592621
c21	GO:0042132~fructose 1,6-bisphosphate 1-phosphatase activity	0.05226223
c21	GO:0008396~oxysterol 7-alpha-hydroxylase activity	0.05226223
c21	GO:0016790~thiolester hydrolase activity	0.053721436
c21	GO:0004806~triacylglycerol lipase activity	0.065734318
c21	GO:0016811~hydrolase activity, acting on carbon-nitrogen (but not peptide) bonds, in linear amides	0.067295225
c21	GO:0048037~cofactor binding	0.067932307
c21	GO:0030693~caspase activity	0.077362477
c21	GO:0016810~hydrolase activity, acting on carbon-nitrogen (but not peptide) bonds	0.085045737
c21	GO:0019200~carbohydrate kinase activity	0.097197448
c22	GO:0018685~alkane 1-monooxygenase activity	0.013689002
c22	GO:0016713~oxidoreductase activity, acting on paired donors, with incorporation or reduction of molecular oxygen, reduced iron-sulfur protein as one donor, and incorporation of one atom of oxygen	0.033875265
c22	GO:0016788~hydrolase activity, acting on ester bonds	0.049800132
c22	GO:0031419~cobalamin binding	0.053652279
c22	GO:0046906~tetrapyrrole binding	0.059167819
c22	GO:0019842~vitamin binding	0.060274347
c24	GO:0022838~substrate specific channel activity	0.003513182
c24	GO:0015267~channel activity	0.00424421
c24	GO:0022803~passive transmembrane transporter activity	0.00430053
c24	GO:0022836~gated channel activity	0.004323625
c24	GO:0046873~metal ion transmembrane transporter activity	0.005673394
c24	GO:0005261~cation channel activity	0.011701786
c24	GO:0005216~ion channel activity	0.012130819
c24	GO:0022857~transmembrane transporter activity	0.013538045
c24	GO:0005244~voltage-gated ion channel activity	0.015932882
c24	GO:0022832~voltage-gated channel activity	0.015932882
c24	GO:0022891~substrate-specific transmembrane transporter activity	0.020595473
c24	GO:0005215~transporter activity	0.029308342
c24	GO:0022843~voltage-gated cation channel activity	0.03644702
c24	GO:0022892~substrate-specific transporter activity	0.048522589
c24	GO:0008324~cation transmembrane transporter activity	0.05756252
c24	GO:0015075~ion transmembrane transporter activity	0.067175145
c24	GO:0005099~Ras GTPase activator activity	0.074766743
c24	GO:0030594~neurotransmitter receptor activity	0.080513805
c24	GO:0005249~voltage-gated potassium channel activity	0.087883861
c24	GO:0042165~neurotransmitter binding	0.09239976
c24	GO:0005096~GTPase activator activity	0.095704184
c25	GO:0005102~receptor binding	0.017569476
c25	GO:0031072~heat shock protein binding	0.047820636

c25	GO:0005515~protein binding	0.087288818
c26	GO:0005515~protein binding	0.012364378
c26	GO:0005391~sodium:potassium-exchanging ATPase activity	0.047884997
c26	GO:0046870~cadmium ion binding	0.047884997
c26	GO:0016884~carbon-nitrogen ligase activity, with glutamine as amido-N-donor	0.047884997
c26	GO:0005200~structural constituent of cytoskeleton	0.049700356
c26	GO:0003743~translation initiation factor activity	0.067610256
c26	GO:0008013~beta-catenin binding	0.091562837
c26	GO:0005125~cytokine activity	0.093610081
c26	GO:0008092~cytoskeletal protein binding	0.098095335
c26	GO:0008527~taste receptor activity	0.099393626
c28	GO:0005515~protein binding	0.018913066
c28	GO:0030247~polysaccharide binding	0.054923176
c28	GO:0001871~pattern binding	0.054923176
c28	GO:0005488~binding	0.057799012
c28	GO:0004385~guanylate kinase activity	0.058483331
c28	GO:0034061~DNA polymerase activity	0.065427863
c28	GO:0005539~glycosaminoglycan binding	0.069780801
c28	GO:0008022~protein C-terminus binding	0.07226973
c28	GO:0008092~cytoskeletal protein binding	0.073478505
c28	GO:0015276~ligand-gated ion channel activity	0.092125215
c28	GO:0022834~ligand-gated channel activity	0.092125215
c28	GO:0004177~aminopeptidase activity	0.094286216
c28	GO:0004693~cyclin-dependent protein kinase activity	0.094286216
c29	GO:0005488~binding	0.006720767
c29	GO:0005515~protein binding	0.025617176
c29	GO:0016788~hydrolase activity, acting on ester bonds	0.068245999
c29	GO:0003723~RNA binding	0.075802247
c29	GO:0016787~hydrolase activity	0.097944299
c31	GO:0004871~signal transducer activity	0.069237455
c31	GO:0060089~molecular transducer activity	0.069237455
c32	GO:0019899~enzyme binding	0.013213602
c32	GO:0005515~protein binding	0.016597346
c32	GO:0003697~single-stranded DNA binding	0.022742372
c32	GO:0016740~transferase activity	0.044826454
c32	GO:0043021~ribonucleoprotein binding	0.053882331
c32	GO:0016853~isomerase activity	0.059686053
c32	GO:0016410~N-acyltransferase activity	0.063891909
c32	GO:0004468~lysine N-acetyltransferase activity	0.065637349
c32	GO:0004402~histone acetyltransferase activity	0.065637349
c32	GO:0008415~acyltransferase activity	0.07178946
c32	GO:0004672~protein kinase activity	0.072300113
c32	GO:0016747~transferase activity, transferring acyl groups other than amino-acyl groups	0.074316327
c32	GO:0046983~protein dimerization activity	0.077490971
c32	GO:0016746~transferase activity, transferring acyl groups	0.083765329

c32	GO:0008318~protein prenyltransferase activity	0.08491315
c32	GO:0030145~manganese ion binding	0.090857992
c32	GO:0004176~ATP-dependent peptidase activity	0.095010243
c33	GO:0016772~transferase activity, transferring phosphorus-containing groups	0.05336614
c33	GO:0003824~catalytic activity	0.065830287
c33	GO:0030554~adenyl nucleotide binding	0.073188969
c33	GO:0001883~purine nucleoside binding	0.078502564
c33	GO:0001882~nucleoside binding	0.081016035
c33	GO:0017076~purine nucleotide binding	0.086400658
c33	GO:0000166~nucleotide binding	0.094408287
c33	GO:0016874~ligase activity	0.098435722
c34	GO:0050660~FAD binding	0.005923547
c34	GO:0050662~coenzyme binding	0.008917472
c34	GO:0005488~binding	0.01277183
c34	GO:0003723~RNA binding	0.018070729
c34	GO:0048037~cofactor binding	0.019216016
c34	GO:0016878~acid-thiol ligase activity	0.02125081
c34	GO:0016405~CoA-ligase activity	0.022555158
c34	GO:0051539~4 iron, 4 sulfur cluster binding	0.038395477
c34	GO:0004385~guanylate kinase activity	0.041740435
c34	GO:0016877~ligase activity, forming carbon-sulfur bonds	0.046733215
c34	GO:0005267~potassium channel activity	0.048445691
c34	GO:0019843~rRNA binding	0.055883361
c34	GO:0005507~copper ion binding	0.057092778
c34	GO:0051082~unfolded protein binding	0.059404626
c34	GO:0051540~metal cluster binding	0.063550224
c34	GO:0051536~iron-sulfur cluster binding	0.063550224
c34	GO:0005515~protein binding	0.070649704
c34	GO:0009055~electron carrier activity	0.072183023
c34	GO:0016776~phosphotransferase activity, phosphate group as acceptor	0.076492551
c34	GO:0048487~beta-tubulin binding	0.082543042
c34	GO:0016874~ligase activity	0.083246506
c34	GO:0016835~carbon-oxygen lyase activity	0.083918289
c34	GO:0016829~lyase activity	0.087131244
c34	GO:0004775~succinate-CoA ligase (ADP-forming) activity	0.087837683
c34	GO:0030955~potassium ion binding	0.092816652
c34	GO:0019842~vitamin binding	0.09873088
c35	GO:0008168~methyltransferase activity	0.02941565
c35	GO:0016741~transferase activity, transferring one-carbon groups	0.030378816
c35	GO:0003824~catalytic activity	0.082573157
c36	GO:0001871~pattern binding	0.009359768
c36	GO:0030247~polysaccharide binding	0.009359768
c36	GO:0005539~glycosaminoglycan binding	0.014174537
c36	GO:0005338~nucleotide-sugar transmembrane transporter activity	0.018463086
c36	GO:0008201~heparin binding	0.022046386

c36	GO:0051861~glycolipid binding	0.023316572
c36	GO:0022891~substrate-specific transmembrane transporter activity	0.027768868
c36	GO:0022857~transmembrane transporter activity	0.034794239
c36	GO:0030674~protein binding, bridging	0.042653316
c36	GO:0015450~P-P-bond-hydrolysis-driven protein transmembrane transporter activity	0.047043973
c36	GO:0022884~macromolecule transmembrane transporter activity	0.047043973
c36	GO:0022892~substrate-specific transporter activity	0.049591861
c36	GO:0005459~UDP-galactose transmembrane transporter activity	0.053547603
c36	GO:0003723~RNA binding	0.054148952
c36	GO:0048037~cofactor binding	0.076688519
c36	GO:0005215~transporter activity	0.0811895
c36	GO:0001664~G-protein-coupled receptor binding	0.081669777
c36	GO:0008320~protein transmembrane transporter activity	0.084494269
c36	GO:0016491~oxidoreductase activity	0.087025408
c37	GO:0008270~zinc ion binding	0.007923151
c37	GO:0046914~transition metal ion binding	0.00940663
c37	GO:0005488~binding	0.025250596
c37	GO:0004861~cyclin-dependent protein kinase inhibitor activity	0.036382036
c37	GO:0004175~endopeptidase activity	0.049665375
c37	GO:0004222~metalloendopeptidase activity	0.056611739
c37	GO:0008233~peptidase activity	0.060370584
c37	GO:0016538~cyclin-dependent protein kinase regulator activity	0.061072698
c37	GO:0030291~protein serine/threonine kinase inhibitor activity	0.061072698
c37	GO:0005515~protein binding	0.074700138
c37	GO:0004843~ubiquitin-specific protease activity	0.081740766
c37	GO:0019783~small conjugating protein-specific protease activity	0.085141726
c38	GO:0005488~binding	1.76E-04
c38	GO:0008238~exopeptidase activity	0.012825501
c38	GO:0008757~S-adenosylmethionine-dependent methyltransferase activity	0.018114
c38	GO:0016614~oxidoreductase activity, acting on CH-OH group of donors	0.018865816
c38	GO:0008168~methyltransferase activity	0.035154561
c38	GO:0016741~transferase activity, transferring one-carbon groups	0.038075342
c38	GO:0016616~oxidoreductase activity, acting on the CH-OH group of donors, NAD or NADP as acceptor	0.040509333
c38	GO:0003824~catalytic activity	0.043156534
c38	GO:0005515~protein binding	0.043526425
c38	GO:0008237~metallopeptidase activity	0.048971325
c38	GO:0005262~calcium channel activity	0.054949479
c38	GO:0003707~steroid hormone receptor activity	0.058721779
c38	GO:0005509~calcium ion binding	0.073193848
c38	GO:0016491~oxidoreductase activity	0.076540117
c38	GO:0048037~cofactor binding	0.083380533
c38	GO:0033764~steroid dehydrogenase activity, acting on the CH-OH group of donors, NAD or NADP as acceptor	0.085186515
c38	GO:0004879~ligand-dependent nuclear receptor activity	0.087577508
c38	GO:0003924~GTPase activity	0.089178174

c38	GO:0046872~metal ion binding	0.095894213
c38	GO:0008233~peptidase activity	0.09832774
c39	GO:0017076~purine nucleotide binding	0.049801078
c39	GO:0005217~intracellular ligand-gated ion channel activity	0.064559176
c39	GO:0032553~ribonucleotide binding	0.073702387
c39	GO:0032555~purine ribonucleotide binding	0.073702387
c39	GO:0000166~nucleotide binding	0.074909157
c5	GO:0003682~chromatin binding	0.008800114
c5	GO:0046983~protein dimerization activity	0.018098044
c5	GO:0016564~transcription repressor activity	0.060234984
c5	GO:0003714~transcription corepressor activity	0.063718368
c5	GO:0003712~transcription cofactor activity	0.083531225
c40	GO:0005515~protein binding	7.41E-04
c40	GO:0008022~protein C-terminus binding	0.010952924
c40	GO:0016787~hydrolase activity	0.01272757
c40	GO:0000287~magnesium ion binding	0.017735146
c40	GO:0005488~binding	0.020549119
c40	GO:0008134~transcription factor binding	0.027841734
c40	GO:0004386~helicase activity	0.030980334
c40	GO:0000166~nucleotide binding	0.037850314
c40	GO:0017111~nucleoside-triphosphatase activity	0.040416668
c40	GO:0016887~ATPase activity	0.055627121
c40	GO:0016462~pyrophosphatase activity	0.057286957
c40	GO:0016818~hydrolase activity, acting on acid anhydrides, in phosphorus-containing anhydrides	0.060459055
c40	GO:0016817~hydrolase activity, acting on acid anhydrides	0.062806364
c40	GO:0004329~formate-tetrahydrofolate ligase activity	0.062828559
c40	GO:0004477~methenyltetrahydrofolate cyclohydrolase activity	0.062828559
c40	GO:0004486~methylenetetrahydrofolate dehydrogenase activity	0.062828559
c40	GO:0031491~nucleosome binding	0.062828559
c40	GO:0016853~isomerase activity	0.066204786
c40	GO:0042393~histone binding	0.067585891
c40	GO:0004725~protein tyrosine phosphatase activity	0.072449618
c40	GO:0004222~metalloendopeptidase activity	0.072449618
c40	GO:0016564~transcription repressor activity	0.077587071
c40	GO:0032555~purine ribonucleotide binding	0.081426457
c40	GO:0032553~ribonucleotide binding	0.081426457
c41	GO:0016455~RNA polymerase II transcription mediator activity	0.01560945
c41	GO:0008134~transcription factor binding	0.020167013
c41	GO:0030374~ligand-dependent nuclear receptor transcription coactivator activity	0.023510064
c41	GO:0005515~protein binding	0.025312758
c41	GO:0003676~nucleic acid binding	0.029611643
c41	GO:0003712~transcription cofactor activity	0.033114563
c41	GO:0016251~general RNA polymerase II transcription factor activity	0.043081876
c41	GO:0003677~DNA binding	0.049557058
c41	GO:0003713~transcription coactivator activity	0.053330464

c41	GO:0001619~lysosphingolipid and lysophosphatidic acid receptor activity	0.082559196
c42	GO:0004221~ubiquitin thiolesterase activity	0.00652348
c42	GO:0016790~thiolester hydrolase activity	0.018601001
c42	GO:0042623~ATPase activity, coupled	0.018731931
c42	GO:0016866~intramolecular transferase activity	0.027116539
c42	GO:0042625~ATPase activity, coupled to transmembrane movement of ions	0.037066793
c42	GO:0004614~phosphoglucomutase activity	0.039041234
c42	GO:0016887~ATPase activity	0.048655233
c42	GO:0008234~cysteine-type peptidase activity	0.051589078
c42	GO:0019901~protein kinase binding	0.058415498
c42	GO:0008233~peptidase activity	0.059347494
c42	GO:0016830~carbon-carbon lyase activity	0.064872129
c42	GO:0022804~active transmembrane transporter activity	0.069537242
c42	GO:0070035~purine NTP-dependent helicase activity	0.073481765
c42	GO:0008026~ATP-dependent helicase activity	0.073481765
c42	GO:0000287~magnesium ion binding	0.080600382
c42	GO:0015077~monovalent inorganic cation transmembrane transporter activity	0.084378707
c42	GO:0042626~ATPase activity, coupled to transmembrane movement of substances	0.09395476
c42	GO:0070011~peptidase activity, acting on L-amino acid peptides	0.095502971
c42	GO:0043492~ATPase activity, coupled to movement of substances	0.095921797
c42	GO:0016820~hydrolase activity, acting on acid anhydrides, catalyzing transmembrane movement of substances	0.09790567
c43	GO:0005488~binding	0.015243956
c43	GO:0003723~RNA binding	0.022826126
c43	GO:0005515~protein binding	0.029965408
c43	GO:0043560~insulin receptor substrate binding	0.042745056
c43	GO:0000166~nucleotide binding	0.052916714
c43	GO:0048037~cofactor binding	0.075949325
c44	GO:0000175~3'-5'-exoribonuclease activity	0.037860824
c44	GO:0016896~exoribonuclease activity, producing 5'-phosphomonoesters	0.043985407
c44	GO:0004532~exoribonuclease activity	0.043985407
c44	GO:0004659~prenyltransferase activity	0.050447591
c44	GO:0016705~oxidoreductase activity, acting on paired donors, with incorporation or reduction of molecular oxygen	0.064213622
c44	GO:0004709~MAP kinase kinase kinase activity	0.087092227
c44	GO:0016796~exonuclease activity, active with either ribo- or deoxyribonucleic acids and producing 5'-phosphomonoesters	0.095153758
c46	GO:0031406~carboxylic acid binding	0.005093758
c46	GO:0004222~metalloendopeptidase activity	0.017830429
c46	GO:0000062~acyl-CoA binding	0.02205256
c46	GO:0008307~structural constituent of muscle	0.022913409
c46	GO:0048037~cofactor binding	0.029341763
c46	GO:0008453~alanine-glyoxylate transaminase activity	0.042957154
c46	GO:0033293~monocarboxylic acid binding	0.04366599
c46	GO:0003824~catalytic activity	0.04441369
c46	GO:0050662~coenzyme binding	0.049197993
c46	GO:0005516~calmodulin binding	0.053578923

c46	GO:0003774~motor activity	0.056285701
c46	GO:0005515~protein binding	0.065965551
c46	GO:0003857~3-hydroxyacyl-CoA dehydrogenase activity	0.084077018
c46	GO:0004175~endopeptidase activity	0.096883307
c6	GO:0005099~Ras GTPase activator activity	0.004473923
c6	GO:0005096~GTPase activator activity	0.015827919
c6	GO:0005515~protein binding	0.01621328
c6	GO:0004970~ionotropic glutamate receptor activity	0.01877535
c6	GO:0005234~extracellular-glutamate-gated ion channel activity	0.02082315
c6	GO:0005083~small GTPase regulator activity	0.04449716
c6	GO:0008047~enzyme activator activity	0.04589932
c6	GO:0005100~Rho GTPase activator activity	0.048690895
c6	GO:0008066~glutamate receptor activity	0.051654104
c6	GO:0005230~extracellular ligand-gated ion channel activity	0.053563002
c6	GO:0005000~vasopressin receptor activity	0.057729804
c6	GO:0005201~extracellular matrix structural constituent	0.081748558
c6	GO:0004568~chitinase activity	0.09076403
c48	GO:0005515~protein binding	0.003092884
c48	GO:0005509~calcium ion binding	0.080211714
c49	GO:0008060~ARF GTPase activator activity	0.004938313
c49	GO:0043515~kinetochore binding	0.01445063
c49	GO:0005096~GTPase activator activity	0.045706941
c49	GO:0030674~protein binding, bridging	0.045777434
c49	GO:0016616~oxidoreductase activity, acting on the CH-OH group of donors, NAD or NADP as acceptor	0.05482921
c49	GO:0030695~GTPase regulator activity	0.058748415
c49	GO:0060589~nucleoside-triphosphatase regulator activity	0.062689512
c49	GO:0016614~oxidoreductase activity, acting on CH-OH group of donors	0.066443643
c49	GO:0005083~small GTPase regulator activity	0.07738703
c51	GO:0016705~oxidoreductase activity, acting on paired donors, with incorporation or reduction of molecular oxygen	0.009837172
c51	GO:0016491~oxidoreductase activity	0.019444181
c51	GO:0019899~enzyme binding	0.022512031
c51	GO:0016462~pyrophosphatase activity	0.025777864
c51	GO:0004721~phosphoprotein phosphatase activity	0.026655491
c51	GO:0016818~hydrolase activity, acting on acid anhydrides, in phosphorus-containing anhydrides	0.02752992
c51	GO:0005488~binding	0.027878976
c51	GO:0016817~hydrolase activity, acting on acid anhydrides	0.028568716
c51	GO:0005525~GTP binding	0.031518877
c51	GO:0017111~nucleoside-triphosphatase activity	0.032259339
c51	GO:0018676~(S)-limonene 7-monooxygenase activity	0.034824324
c51	GO:0018675~(S)-limonene 6-monooxygenase activity	0.034824324
c51	GO:0033767~4-hydroxyacetophenone monooxygenase activity	0.034824324
c51	GO:0019001~guanyl nucleotide binding	0.037830836
c51	GO:0032561~guanyl ribonucleotide binding	0.037830836
c51	GO:0005506~iron ion binding	0.045940767

c51	GO:0030544~Hsp70 protein binding	0.068440315
c51	GO:0016787~hydrolase activity	0.075960198
c51	GO:0003924~GTPase activity	0.079171826
c51	GO:0016874~ligase activity	0.083070347
c51	GO:0016247~channel regulator activity	0.085112664
c51	GO:0016709~oxidoreductase activity, acting on paired donors, with incorporation or reduction of molecular oxygen, NADH or NADPH as one donor, and incorporation of one atom of oxygen	0.086219839
c51	GO:0004497~monooxygenase activity	0.09141011
c51	GO:0043028~caspase regulator activity	0.091571064
c52	GO:0003723~RNA binding	9.51E-05
c52	GO:0005515~protein binding	4.79E-04
c52	GO:0031369~translation initiation factor binding	0.015315321
c52	GO:0032559~adenyl ribonucleotide binding	0.023609356
c52	GO:0003676~nucleic acid binding	0.025882756
c52	GO:0005524~ATP binding	0.028913198
c52	GO:0008134~transcription factor binding	0.032026778
c52	GO:0004672~protein kinase activity	0.041883156
c52	GO:0030554~adenyl nucleotide binding	0.049987336
c52	GO:0032553~ribonucleotide binding	0.053644389
c52	GO:0032555~purine ribonucleotide binding	0.053644389
c52	GO:0001883~purine nucleoside binding	0.06055349
c52	GO:0070035~purine NTP-dependent helicase activity	0.062347423
c52	GO:0008026~ATP-dependent helicase activity	0.062347423
c52	GO:0005488~binding	0.06247366
c52	GO:0001882~nucleoside binding	0.066140275
c52	GO:0000166~nucleotide binding	0.066210364
c52	GO:0016773~phosphotransferase activity, alcohol group as acceptor	0.068382088
c52	GO:0019787~small conjugating protein ligase activity	0.070545018
c52	GO:0016790~thiolester hydrolase activity	0.071433867
c52	GO:0005102~receptor binding	0.073246045
c52	GO:0004221~ubiquitin thiolesterase activity	0.080303631
c52	GO:0004386~helicase activity	0.084998786
c52	GO:0016564~transcription repressor activity	0.085031094
c52	GO:0005097~Rab GTPase activator activity	0.086021597
c52	GO:0017076~purine nucleotide binding	0.096876901
c52	GO:0003712~transcription cofactor activity	0.098885605
c54	GO:0004175~endopeptidase activity	0.012081541
c54	GO:0008233~peptidase activity	0.013568929
c54	GO:0003824~catalytic activity	0.017064989
c54	GO:0004252~serine-type endopeptidase activity	0.018998518
c54	GO:0030674~protein binding, bridging	0.024300635
c54	GO:0070011~peptidase activity, acting on L-amino acid peptides	0.028747345
c54	GO:0008236~serine-type peptidase activity	0.030281601
c54	GO:0017171~serine hydrolase activity	0.03136736
c54	GO:0005515~protein binding	0.040072562
c54	GO:0019904~protein domain specific binding	0.068183848

c55	GO:0016706~oxidoreductase activity, acting on paired donors, with incorporation or reduction of molecular oxygen, 2-oxoglutarate as one donor, and incorporation of one atom each of oxygen into both donors	0.006425278
c55	GO:0005545~phosphatidylinositol binding	0.008430235
c55	GO:0015036~disulfide oxidoreductase activity	0.011803775
c55	GO:0019901~protein kinase binding	0.018390424
c55	GO:0035091~phosphoinositide binding	0.01861821
c55	GO:0042277~peptide binding	0.022112699
c55	GO:0003824~catalytic activity	0.028774258
c55	GO:0003993~acid phosphatase activity	0.034065395
c55	GO:0005044~scavenger receptor activity	0.035107597
c55	GO:0031406~carboxylic acid binding	0.041710815
c55	GO:0008066~glutamate receptor activity	0.050317379
c55	GO:0015035~protein disulfide oxidoreductase activity	0.053452967
c55	GO:0019900~kinase binding	0.054203663
c55	GO:0008238~exopeptidase activity	0.057095037
c55	GO:0016853~isomerase activity	0.069056134
c55	GO:0016597~amino acid binding	0.078617625
c55	GO:0003680~AT DNA binding	0.07886382
c55	GO:0042808~neuronal Cdc2-like kinase binding	0.07886382
c55	GO:0016773~phosphotransferase activity, alcohol group as acceptor	0.08195311
c55	GO:0016667~oxidoreductase activity, acting on sulfur group of donors	0.087594483
c55	GO:0004672~protein kinase activity	0.097550896
c56	GO:0005310~dicarboxylic acid transmembrane transporter activity	0.006838091
c56	GO:0003713~transcription coactivator activity	0.026892202
c56	GO:0046943~carboxylic acid transmembrane transporter activity	0.035865206
c56	GO:0005342~organic acid transmembrane transporter activity	0.036854549
c56	GO:0005488~binding	0.038029306
c56	GO:0016563~transcription activator activity	0.04295232
c56	GO:0005515~protein binding	0.059404789
c56	GO:0003712~transcription cofactor activity	0.066660123
c56	GO:0017153~sodium:dicarboxylate symporter activity	0.075884405
c56	GO:0015171~amino acid transmembrane transporter activity	0.07845604
c57	GO:0016787~hydrolase activity	0.010753944
c57	GO:0003824~catalytic activity	0.022925602
c57	GO:0016757~transferase activity, transferring glycosyl groups	0.038040641
c57	GO:0030246~carbohydrate binding	0.083169269
c57	GO:0008236~serine-type peptidase activity	0.094353866
c57	GO:0017171~serine hydrolase activity	0.096147781
c7	GO:0003824~catalytic activity	0.001836754
c7	GO:0016740~transferase activity	0.002867292
c7	GO:0005524~ATP binding	0.008135634
c7	GO:0030554~adenyl nucleotide binding	0.008429216
c7	GO:0001882~nucleoside binding	0.009872228
c7	GO:0032559~adenyl ribonucleotide binding	0.01078559
c7	GO:0001883~purine nucleoside binding	0.011927001

c7	GO:0008417~fucosyltransferase activity	0.012797311
c7	GO:0016769~transferase activity, transferring nitrogenous groups	0.022178572
c7	GO:0046920~alpha(1,3)-fucosyltransferase activity	0.031774579
c7	GO:0001786~phosphatidylserine binding	0.039887246
c7	GO:0070279~vitamin B6 binding	0.045359235
c7	GO:0030170~pyridoxal phosphate binding	0.045359235
c7	GO:0008168~methyltransferase activity	0.045550418
c7	GO:0016620~oxidoreductase activity, acting on the aldehyde or oxo group of donors, NAD or NADP as acceptor	0.048992871
c7	GO:0016741~transferase activity, transferring one-carbon groups	0.05052399
c7	GO:0016887~ATPase activity	0.061285639
c7	GO:0016491~oxidoreductase activity	0.062609679
c7	GO:0004674~protein serine/threonine kinase activity	0.064684982
c7	GO:0005516~calmodulin binding	0.068547064
c7	GO:0042623~ATPase activity, coupled	0.07037599
c7	GO:0019166~trans-2-enoyl-CoA reductase (NADPH) activity	0.071196678
c7	GO:0004672~protein kinase activity	0.077531349
c7	GO:0008757~S-adenosylmethionine-dependent methyltransferase activity	0.084230099
c7	GO:0008060~ARF GTPase activator activity	0.086371992
c7	GO:0016645~oxidoreductase activity, acting on the CH-NH group of donors	0.086371992
c7	GO:0016409~palmitoyltransferase activity	0.089562713
c7	GO:0004177~aminopeptidase activity	0.093504594
c58	GO:0005488~binding	0.001441876
c58	GO:0016740~transferase activity	0.001623311
c58	GO:0005515~protein binding	0.013700635
c58	GO:0008565~protein transporter activity	0.016274359
c58	GO:0016772~transferase activity, transferring phosphorus-containing groups	0.026667478
c58	GO:0015301~anion:anion antiporter activity	0.037221734
c58	GO:0051378~serotonin binding	0.042447876
c58	GO:0046906~tetrapyrrole binding	0.053743546
c58	GO:0022804~active transmembrane transporter activity	0.055897159
c58	GO:0008227~amine receptor activity	0.066560292
c58	GO:0016773~phosphotransferase activity, alcohol group as acceptor	0.070820452
c58	GO:0008061~chitin binding	0.073613613
c58	GO:0018685~alkane 1-monooxygenase activity	0.073613613
c58	GO:0008135~translation factor activity, nucleic acid binding	0.075188192
c58	GO:0004993~serotonin receptor activity	0.083371363
c58	GO:0016791~phosphatase activity	0.086344658
c58	GO:0004722~protein serine/threonine phosphatase activity	0.087339056
c58	GO:0016301~kinase activity	0.091701781
c58	GO:0005215~transporter activity	0.092389088
c58	GO:0046982~protein heterodimerization activity	0.092596262
c59	GO:0005198~structural molecule activity	0.042950049
c59	GO:0004175~endopeptidase activity	0.079302693
c60	GO:0051879~Hsp90 protein binding	0.015089598
c60	GO:0042802~identical protein binding	0.071059214

c60	GO:0005488~binding	0.074286134
c60	GO:0016597~amino acid binding	0.087328041
c62	GO:0015085~calcium ion transmembrane transporter activity	0.020165105
c62	GO:0003725~double-stranded RNA binding	0.023095228
c62	GO:0016874~ligase activity	0.031081083
c62	GO:0030528~transcription regulator activity	0.034517973
c62	GO:0005217~intracellular ligand-gated ion channel activity	0.034919663
c62	GO:0017163~basal transcription repressor activity	0.036899342
c62	GO:0003702~RNA polymerase II transcription factor activity	0.039215844
c62	GO:0046332~SMAD binding	0.053952748
c62	GO:0042802~identical protein binding	0.054406184
c62	GO:0005220~inositol 1,4,5-trisphosphate-sensitive calcium-release channel activity	0.054837021
c62	GO:0003682~chromatin binding	0.061787068
c62	GO:0005515~protein binding	0.06323019
c62	GO:0030551~cyclic nucleotide binding	0.067653914
c62	GO:0022838~substrate specific channel activity	0.068724916
c62	GO:0008095~inositol-1,4,5-trisphosphate receptor activity	0.072441774
c62	GO:0004694~eukaryotic translation initiation factor 2alpha kinase activity	0.072441774
c62	GO:0022803~passive transmembrane transporter activity	0.085685668
c62	GO:0015267~channel activity	0.08636853
c62	GO:0022834~ligand-gated channel activity	0.088801158
c62	GO:0015276~ligand-gated ion channel activity	0.088801158
c62	GO:0008467~[heparan sulfate]-glucosamine 3-sulfotransferase 1 activity	0.089719759
c62	GO:0010843~promoter binding	0.089866941
c62	GO:0042803~protein homodimerization activity	0.094279348
c62	GO:0003700~transcription factor activity	0.099116089
c63	GO:0000166~nucleotide binding	0.001741639
c63	GO:0003723~RNA binding	0.002528013
c63	GO:0005515~protein binding	0.00488241
c63	GO:0016740~transferase activity	0.018699735
c63	GO:0017076~purine nucleotide binding	0.019178425
c63	GO:0019899~enzyme binding	0.020423405
c63	GO:0003824~catalytic activity	0.022642791
c63	GO:0032553~ribonucleotide binding	0.023891052
c63	GO:0032555~purine ribonucleotide binding	0.023891052
c63	GO:0005488~binding	0.025104408
c63	GO:0050662~coenzyme binding	0.025928217
c63	GO:0004672~protein kinase activity	0.033012958
c63	GO:0030554~adenyl nucleotide binding	0.041302756
c63	GO:0000049~tRNA binding	0.043429309
c63	GO:0005524~ATP binding	0.044559926
c63	GO:0048037~cofactor binding	0.045445328
c63	GO:0001883~purine nucleoside binding	0.049373147
c63	GO:0032559~adenyl ribonucleotide binding	0.051849376
c63	GO:0004527~exonuclease activity	0.052141772

c63	GO:0001882~nucleoside binding	0.053442341
c63	GO:0016773~phosphotransferase activity, alcohol group as acceptor	0.068562845
c63	GO:0004674~protein serine/threonine kinase activity	0.083169761
c63	GO:0016787~hydrolase activity	0.098830433
c64	GO:0005488~binding	2.29E-04
c64	GO:0005515~protein binding	0.001004761
c64	GO:0004386~helicase activity	0.001297898
c64	GO:0070035~purine NTP-dependent helicase activity	0.010651571
c64	GO:0008026~ATP-dependent helicase activity	0.010651571
c64	GO:0004197~cysteine-type endopeptidase activity	0.023271609
c64	GO:0016615~malate dehydrogenase activity	0.023397187
c64	GO:0008234~cysteine-type peptidase activity	0.030293474
c64	GO:0003678~DNA helicase activity	0.033802704
c64	GO:0000166~nucleotide binding	0.040102319
c64	GO:0004003~ATP-dependent DNA helicase activity	0.040321431
c64	GO:0019205~nucleobase, nucleoside, nucleotide kinase activity	0.042513073
c64	GO:0003676~nucleic acid binding	0.043500049
c64	GO:0004019~adenylosuccinate synthase activity	0.060601563
c64	GO:0017111~nucleoside-triphosphatase activity	0.064069317
c64	GO:0016462~pyrophosphatase activity	0.064718575
c64	GO:0016614~oxidoreductase activity, acting on CH-OH group of donors	0.066972247
c64	GO:0016818~hydrolase activity, acting on acid anhydrides, in phosphorus-containing anhydrides	0.067674686
c64	GO:0016817~hydrolase activity, acting on acid anhydrides	0.071139058
c64	GO:0042301~phosphate binding	0.076195346
c64	GO:0042162~telomeric DNA binding	0.076195346
c64	GO:0016776~phosphotransferase activity, phosphate group as acceptor	0.080062345
c64	GO:0030528~transcription regulator activity	0.082481985
c64	GO:0035257~nuclear hormone receptor binding	0.088424886
c64	GO:0005049~nuclear export signal receptor activity	0.089513767
c64	GO:0016779~nucleotidyltransferase activity	0.091212847
c64	GO:0003682~chromatin binding	0.092131453
c64	GO:0016887~ATPase activity	0.093715136
c64	GO:0016874~ligase activity	0.095769091
c64	GO:0003712~transcription cofactor activity	0.097621657
c64	GO:0008094~DNA-dependent ATPase activity	0.09801182
c64	GO:0004860~protein kinase inhibitor activity	0.098078707
c64	GO:0032553~ribonucleotide binding	0.098625026
c64	GO:0032555~purine ribonucleotide binding	0.098625026
c65	GO:0005515~protein binding	0.033148522
c65	GO:0008080~N-acetyltransferase activity	0.078959996
c65	GO:0016407~acetyltransferase activity	0.093161307
c65	GO:0016410~N-acyltransferase activity	0.094245106
c66	GO:0003677~DNA binding	0.041737083
c66	GO:0005515~protein binding	0.077276458
c67	GO:0003676~nucleic acid binding	0.052368736

c67	GO:0046966~thyroid hormone receptor binding	0.085125531
c67	GO:0030374~ligand-dependent nuclear receptor transcription coactivator activity	0.095255531
c67	GO:0008270~zinc ion binding	0.096669731
c8	GO:0031420~alkali metal ion binding	0.04135071
c8	GO:0005215~transporter activity	0.041383288
c8	GO:0004497~monooxygenase activity	0.042266492
c8	GO:0031402~sodium ion binding	0.059059267
c8	GO:0016705~oxidoreductase activity, acting on paired donors, with incorporation or reduction of molecular oxygen	0.075802199
c8	GO:0016709~oxidoreductase activity, acting on paired donors, with incorporation or reduction of molecular oxygen, NADH or NADPH as one donor, and incorporation of one atom of oxygen	0.09021285
c8	GO:0043167~ion binding	0.096138941
c68	GO:0003824~catalytic activity	0.074258479
c68	GO:0005215~transporter activity	0.088099031
c70	GO:0016866~intramolecular transferase activity	0.061674211
c70	GO:0005125~cytokine activity	0.081941748
c70	GO:0003824~catalytic activity	0.095596307
c73	GO:0008324~cation transmembrane transporter activity	0.027645963
c73	GO:0015075~ion transmembrane transporter activity	0.037243399
c73	GO:0022892~substrate-specific transporter activity	0.067925489
c73	GO:0022891~substrate-specific transmembrane transporter activity	0.070028708
c73	GO:0005488~binding	0.096586907
c74	GO:0005515~protein binding	0.01691779
c74	GO:0008234~cysteine-type peptidase activity	0.023413421
c74	GO:0000287~magnesium ion binding	0.036112975
c74	GO:0005488~binding	0.03852562
c74	GO:0004197~cysteine-type endopeptidase activity	0.049994816
c74	GO:0035257~nuclear hormone receptor binding	0.058866243
c74	GO:0046906~tetrapyrrole binding	0.061553562
c74	GO:0005506~iron ion binding	0.065230336
c74	GO:0050733~RS domain binding	0.067003114
c74	GO:0051427~hormone receptor binding	0.080757369
c74	GO:0008009~chemokine activity	0.097899271
c75	GO:0016564~transcription repressor activity	0.053310822
c76	GO:0003723~RNA binding	0.004434161
c76	GO:0003676~nucleic acid binding	0.037422966
c76	GO:0015276~ligand-gated ion channel activity	0.041167226
c76	GO:0022834~ligand-gated channel activity	0.041167226
c76	GO:0043498~cell surface binding	0.057679546
c76	GO:0005097~Rab GTPase activator activity	0.058446996
c76	GO:0005099~Ras GTPase activator activity	0.060051425
c76	GO:0016772~transferase activity, transferring phosphorus-containing groups	0.063982243
c76	GO:0046966~thyroid hormone receptor binding	0.073286332
c76	GO:0005096~GTPase activator activity	0.075533781
c76	GO:0005261~cation channel activity	0.080192069
c76	GO:0015300~solute:solute antiporter activity	0.082618629

c76	GO:0000268~peroxisome targeting sequence binding	0.0889553
c76	GO:0000702~oxidized base lesion DNA N-glycosylase activity	0.0889553
c76	GO:0005017~platelet-derived growth factor receptor activity	0.0889553
c76	GO:0022836~gated channel activity	0.094929798
c76	GO:0001614~purinergic nucleotide receptor activity	0.096650256
c76	GO:0016502~nucleotide receptor activity	0.096650256
c76	GO:0004725~protein tyrosine phosphatase activity	0.099206899
c77	GO:0003824~catalytic activity	7.38E-04
c77	GO:0016740~transferase activity	7.67E-04
c77	GO:0000166~nucleotide binding	0.001326121
c77	GO:0005524~ATP binding	0.001678451
c77	GO:0032553~ribonucleotide binding	0.001740803
c77	GO:0032555~purine ribonucleotide binding	0.001740803
c77	GO:0017076~purine nucleotide binding	0.001895296
c77	GO:0032559~adenyl ribonucleotide binding	0.002055331
c77	GO:0030554~adenyl nucleotide binding	0.002254418
c77	GO:0001883~purine nucleoside binding	0.002831255
c77	GO:0001882~nucleoside binding	0.00313546
c77	GO:0003723~RNA binding	0.006223029
c77	GO:0004659~prenyltransferase activity	0.010200595
c77	GO:0016772~transferase activity, transferring phosphorus-containing groups	0.010583297
c77	GO:0017111~nucleoside-triphosphatase activity	0.015539561
c77	GO:0016462~pyrophosphatase activity	0.021300342
c77	GO:0005488~binding	0.021665422
c77	GO:0016818~hydrolase activity, acting on acid anhydrides, in phosphorus-containing anhydrides	0.021978206
c77	GO:0004662~CAAX-protein geranylgeranyltransferase activity	0.022066112
c77	GO:0016817~hydrolase activity, acting on acid anhydrides	0.022907349
c77	GO:0032139~dinucleotide insertion or deletion binding	0.032916974
c77	GO:0004672~protein kinase activity	0.037648057
c77	GO:0008168~methyltransferase activity	0.04109086
c77	GO:0016787~hydrolase activity	0.042084192
c77	GO:0016741~transferase activity, transferring one-carbon groups	0.043640014
c77	GO:0016779~nucleotidyltransferase activity	0.050505361
c77	GO:0004674~protein serine/threonine kinase activity	0.050532571
c77	GO:0003847~1-alkyl-2-acetylglycerophosphocholine esterase activity	0.054260942
c77	GO:0008409~5'-3' exonuclease activity	0.054260942
c77	GO:0032135~DNA insertion or deletion binding	0.054260942
c77	GO:0070569~uridylyltransferase activity	0.064756659
c77	GO:0004661~protein geranylgeranyltransferase activity	0.064756659
c77	GO:0032405~MutLalpha complex binding	0.064756659
c77	GO:0000287~magnesium ion binding	0.064994647
c77	GO:0004386~helicase activity	0.070536707
c77	GO:0008022~protein C-terminus binding	0.071998242
c77	GO:0016301~kinase activity	0.080200375
c77	GO:0008318~protein prenyltransferase activity	0.085401978

c77	GO:0032404~mismatch repair complex binding	0.085401978
c77	GO:0016763~transferase activity, transferring pentosyl groups	0.092275861
c77	GO:0030165~PDZ domain binding	0.095700582
c9	GO:0000287~magnesium ion binding	0.045647419
c9	GO:0005515~protein binding	0.04863162
c9	GO:0005488~binding	0.052194044
c80	GO:0016787~hydrolase activity	0.010406353
c80	GO:0016462~pyrophosphatase activity	0.014279691
c80	GO:0016818~hydrolase activity, acting on acid anhydrides, in phosphorus-containing anhydrides	0.015240848
c80	GO:0016817~hydrolase activity, acting on acid anhydrides	0.016115857
c80	GO:0017110~nucleoside-diphosphatase activity	0.024935689
c80	GO:0004222~metalloendopeptidase activity	0.027399663
c80	GO:0017111~nucleoside-triphosphatase activity	0.037818568
c80	GO:0016763~transferase activity, transferring pentosyl groups	0.039842855
c80	GO:0005488~binding	0.043073346
c80	GO:0005515~protein binding	0.045522802
c80	GO:0016887~ATPase activity	0.051214727
c80	GO:0004527~exonuclease activity	0.061497712
c80	GO:0004175~endopeptidase activity	0.06535405
c80	GO:0003676~nucleic acid binding	0.06648316
c80	GO:0003697~single-stranded DNA binding	0.068625416
c80	GO:0003824~catalytic activity	0.070561328
c80	GO:0015077~monovalent inorganic cation transmembrane transporter activity	0.073090909
c80	GO:0016811~hydrolase activity, acting on carbon-nitrogen (but not peptide) bonds, in linear amides	0.080076171
c80	GO:0016407~acetyltransferase activity	0.081155351
c80	GO:0003985~acetyl-CoA C-acetyltransferase activity	0.082048762
c80	GO:0042975~peroxisome proliferator activated receptor binding	0.082048762
c80	GO:0042623~ATPase activity, coupled	0.084541102
c80	GO:0000149~SNARE binding	0.085092797
c80	GO:0004468~lysine N-acetyltransferase activity	0.090548705
c80	GO:0004402~histone acetyltransferase activity	0.090548705
c80	GO:0003677~DNA binding	0.091484482
c80	GO:0070011~peptidase activity, acting on L-amino acid peptides	0.094545612
c80	GO:0004709~MAP kinase kinase kinase activity	0.098612339
c81	GO:0003824~catalytic activity	0.03060929
c81	GO:0070001~aspartic-type peptidase activity	0.066206553
c81	GO:0004190~aspartic-type endopeptidase activity	0.066206553
c81	GO:0051059~NF-kappaB binding	0.082760575
c81	GO:0016301~kinase activity	0.099887066
c10	GO:0015103~inorganic anion transmembrane transporter activity	0.008123682
c10	GO:0003824~catalytic activity	0.010958652
c10	GO:0016779~nucleotidyltransferase activity	0.01148135
c10	GO:0008509~anion transmembrane transporter activity	0.027619282
c10	GO:0004129~cytochrome-c oxidase activity	0.029964614
c10	GO:0016675~oxidoreductase activity, acting on heme group of donors	0.029964614

c10	GO:0016676~oxidoreductase activity, acting on heme group of donors, oxygen as acceptor	0.029964614
c10	GO:0015002~heme-copper terminal oxidase activity	0.029964614
c10	GO:0003684~damaged DNA binding	0.033173789
c10	GO:0004629~phospholipase C activity	0.038988283
c10	GO:0004175~endopeptidase activity	0.041980404
c10	GO:0022892~substrate-specific transporter activity	0.047944293
c10	GO:0005165~neurotrophin receptor binding	0.048143175
c10	GO:0022891~substrate-specific transmembrane transporter activity	0.049574869
c10	GO:0008144~drug binding	0.049935186
c10	GO:0015075~ion transmembrane transporter activity	0.051578978
c10	GO:0004620~phospholipase activity	0.051961655
c10	GO:0030246~carbohydrate binding	0.052216779
c10	GO:0016782~transferase activity, transferring sulfur-containing groups	0.052646953
c10	GO:0008081~phosphoric diester hydrolase activity	0.054198056
c10	GO:0022857~transmembrane transporter activity	0.054665164
c10	GO:0042623~ATPase activity, coupled	0.070116267
c10	GO:0000702~oxidized base lesion DNA N-glycosylase activity	0.071340795
c10	GO:0030983~mismatched DNA binding	0.077121316
c10	GO:0015405~P-P-bond-hydrolysis-driven transmembrane transporter activity	0.07791467
c10	GO:0015399~primary active transmembrane transporter activity	0.07791467
c10	GO:0034062~RNA polymerase activity	0.086751775
c10	GO:0003899~DNA-directed RNA polymerase activity	0.086751775
c10	GO:0070035~purine NTP-dependent helicase activity	0.091232773
c10	GO:0008026~ATP-dependent helicase activity	0.091232773
c10	GO:0003896~DNA primase activity	0.093974563
c10	GO:0016298~lipase activity	0.097374415
