# Supplementary material for: Construction of microRNA functional families by a mixture model of position weight matrices
Source: PeerJ. 2013 Oct 31;1:e199. doi: 10.7717/peerj.199 (PMC3817585; doi:10.7717/peerj.199)
Supplement: Table S3 [file peerj-01-199-s004.pdf]

Supplemental Table S3. GO (Biological Process) enrichment analyses for target genes in each group

cluster ID	GO Term (Biological Process)	p-value
c1	GO:0006694~steroid biosynthetic process	0.047180627
c1	GO:0009894~regulation of catabolic process	0.053141656
c1	GO:0016125~sterol metabolic process	0.055840409
c1	GO:0060255~regulation of macromolecule metabolic process	0.0889807
c1	GO:0080090~regulation of primary metabolic process	0.091737911
c2	GO:0006812~cation transport	0.009180914
c2	GO:0030001~metal ion transport	0.024943181
c2	GO:0050851~antigen receptor-mediated signaling pathway	0.025797294
c2	GO:0002429~immune response-activating cell surface receptor signaling pathway	0.035160585
c2	GO:0051726~regulation of cell cycle	0.040015623
c2	GO:0002768~immune response-regulating cell surface receptor signaling pathway	0.040263106
c2	GO:0051641~cellular localization	0.048132576
c2	GO:0006810~transport	0.054566961
c2	GO:0051649~establishment of localization in cell	0.057861366
c2	GO:0031122~cytoplasmic microtubule organization	0.059070028
c2	GO:0002757~immune response-activating signal transduction	0.05907717
c2	GO:0051234~establishment of localization	0.060682493
c2	GO:0046907~intracellular transport	0.061452711
c2	GO:0006811~ion transport	0.065887809
c2	GO:0009987~cellular process	0.067130249
c2	GO:0002764~immune response-regulating signal transduction	0.067301131
c2	GO:0002682~regulation of immune system process	0.072408269
c2	GO:0006814~sodium ion transport	0.076078511
c2	GO:0051179~localization	0.086447019
c2	GO:0050776~regulation of immune response	0.092835096
c2	GO:0015672~monovalent inorganic cation transport	0.094117366
c11	GO:0009611~response to wounding	0.004186403
c11	GO:0002673~regulation of acute inflammatory response	0.004657051
c11	GO:0051239~regulation of multicellular organismal process	0.00546274
c11	GO:0032101~regulation of response to external stimulus	0.007597396
c11	GO:0060264~regulation of respiratory burst during acute inflammatory response	0.009752592
c11	GO:0080134~regulation of response to stress	0.010872722
c11	GO:0001817~regulation of cytokine production	0.011835224
c11	GO:0002697~regulation of immune effector process	0.013243582
c11	GO:0060267~positive regulation of respiratory burst	0.014593677
c11	GO:0006521~regulation of cellular amino acid metabolic process	0.014593677
c11	GO:0006468~protein amino acid phosphorylation	0.015707252
c11	GO:0006954~inflammatory response	0.021244999
c11	GO:0045793~positive regulation of cell size	0.02298524
c11	GO:0050776~regulation of immune response	0.024618497
c11	GO:0048583~regulation of response to stimulus	0.025862571
c11	GO:0048584~positive regulation of response to stimulus	0.028271781
c11	GO:0045087~innate immune response	0.029990948

c11	GO:0006952~defense response	0.030134383
c11	GO:0006950~response to stress	0.031509223
c11	GO:0031347~regulation of defense response	0.032829898
c11	GO:0009605~response to external stimulus	0.033190265
c11	GO:0048518~positive regulation of biological process	0.033699594
c11	GO:0060263~regulation of respiratory burst	0.033725865
c11	GO:0032103~positive regulation of response to external stimulus	0.039086014
c11	GO:0016310~phosphorylation	0.040634988
c11	GO:0042135~neurotransmitter catabolic process	0.043154207
c11	GO:0051241~negative regulation of multicellular organismal process	0.046224727
c11	GO:0050727~regulation of inflammatory response	0.053241862
c11	GO:0015674~di-, tri-valent inorganic cation transport	0.054919078
c11	GO:0002675~positive regulation of acute inflammatory response	0.057126971
c11	GO:0045927~positive regulation of growth	0.058309257
c11	GO:0033238~regulation of cellular amine metabolic process	0.061739721
c11	GO:0042402~biogenic amine catabolic process	0.070898606
c11	GO:0032760~positive regulation of tumor necrosis factor production	0.070898606
c11	GO:0007218~neuropeptide signaling pathway	0.075846293
c11	GO:0009893~positive regulation of metabolic process	0.079275823
c11	GO:0002526~acute inflammatory response	0.082986763
c11	GO:0040008~regulation of growth	0.085419606
c11	GO:0042127~regulation of cell proliferation	0.088862043
c11	GO:0042219~cellular amino acid derivative catabolic process	0.088952844
c11	GO:0051329~interphase of mitotic cell cycle	0.090325171
c11	GO:0051325~interphase	0.094817615
c11	GO:0010557~positive regulation of macromolecule biosynthetic process	0.099627163
c12	GO:0016568~chromatin modification	0.001521752
c12	GO:0003015~heart process	0.004440924
c12	GO:0060047~heart contraction	0.004440924
c12	GO:0006996~organelle organization	0.00858176
c12	GO:0006325~chromatin organization	0.009823607
c12	GO:0016570~histone modification	0.010947846
c12	GO:0016569~covalent chromatin modification	0.013331044
c12	GO:0016043~cellular component organization	0.016329685
c12	GO:0060048~cardiac muscle contraction	0.018006559
c12	GO:0010467~gene expression	0.022521849
c12	GO:0010556~regulation of macromolecule biosynthetic process	0.026651934
c12	GO:0006942~regulation of striated muscle contraction	0.026795297
c12	GO:0055010~ventricular cardiac muscle morphogenesis	0.026795297
c12	GO:0031323~regulation of cellular metabolic process	0.027260351
c12	GO:0045727~positive regulation of translation	0.030148453
c12	GO:0051276~chromosome organization	0.034850914
c12	GO:0006275~regulation of DNA replication	0.037546913
c12	GO:0048522~positive regulation of cellular process	0.038482745
c12	GO:0019219~regulation of nucleobase, nucleoside, nucleotide and nucleic acid metabolic process	0.039598249

c12	GO:0044238~primary metabolic process	0.041482289
c12	GO:0016311~dephosphorylation	0.041793793
c12	GO:0031326~regulation of cellular biosynthetic process	0.041812398
c12	GO:0045449~regulation of transcription	0.042403924
c12	GO:0008284~positive regulation of cell proliferation	0.042683809
c12	GO:0010468~regulation of gene expression	0.04308786
c12	GO:0060415~muscle tissue morphogenesis	0.04564226
c12	GO:0055008~cardiac muscle tissue morphogenesis	0.04564226
c12	GO:0043170~macromolecule metabolic process	0.045831327
c12	GO:0006470~protein amino acid dephosphorylation	0.046234793
c12	GO:0006941~striated muscle contraction	0.047970671
c12	GO:0051171~regulation of nitrogen compound metabolic process	0.048350802
c12	GO:0009889~regulation of biosynthetic process	0.048495134
c12	GO:0003013~circulatory system process	0.052974541
c12	GO:0008015~blood circulation	0.052974541
c12	GO:0016573~histone acetylation	0.054641594
c12	GO:0045995~regulation of embryonic development	0.055947866
c12	GO:0033206~cytokinesis after meiosis	0.058898643
c12	GO:0030007~cellular potassium ion homeostasis	0.058898643
c12	GO:0040038~polar body extrusion after meiotic divisions	0.058898643
c12	GO:0009152~purine ribonucleotide biosynthetic process	0.061401567
c12	GO:0006937~regulation of muscle contraction	0.064044963
c12	GO:0006664~glycolipid metabolic process	0.064331852
c12	GO:0051270~regulation of cell motion	0.064669769
c12	GO:0048518~positive regulation of biological process	0.067167431
c12	GO:0030334~regulation of cell migration	0.067370888
c12	GO:0019222~regulation of metabolic process	0.068172415
c12	GO:0006473~protein amino acid acetylation	0.069400927
c12	GO:0000245~spliceosome assembly	0.06947572
c12	GO:0008152~metabolic process	0.069565601
c12	GO:0016070~RNA metabolic process	0.073271268
c12	GO:0009260~ribonucleotide biosynthetic process	0.078450338
c12	GO:0080090~regulation of primary metabolic process	0.079706917
c12	GO:0050885~neuromuscular process controlling balance	0.085965924
c12	GO:0006177~GMP biosynthetic process	0.087037
c12	GO:0046037~GMP metabolic process	0.087037
c12	GO:0048738~cardiac muscle tissue development	0.090417174
c12	GO:0006643~membrane lipid metabolic process	0.094935063
c12	GO:0030518~steroid hormone receptor signaling pathway	0.094947999
c12	GO:0044237~cellular metabolic process	0.097090416
c12	GO:0060255~regulation of macromolecule metabolic process	0.098121459
c12	GO:0006807~nitrogen compound metabolic process	0.09938351
c13	GO:0051716~cellular response to stimulus	0.062104687
c13	GO:0006974~response to DNA damage stimulus	0.065479492
c13	GO:0006766~vitamin metabolic process	0.081746696

c13	GO:0019748~secondary metabolic process	0.085925465
c14	GO:0050794~regulation of cellular process	0.02703226
c14	GO:0050789~regulation of biological process	0.048243331
c14	GO:0065007~biological regulation	0.094317683
c16	GO:0034660~ncRNA metabolic process	0.049308908
c16	GO:0006396~RNA processing	0.051572676
c16	GO:0016070~RNA metabolic process	0.054363906
c16	GO:0007588~excretion	0.086660558
c16	GO:0032501~multicellular organismal process	0.09718245
c17	GO:0008380~RNA splicing	0.071565319
c17	GO:0006397~mRNA processing	0.088388199
c18	GO:0007018~microtubule-based movement	0.002229277
c18	GO:0010468~regulation of gene expression	0.004970589
c18	GO:0009889~regulation of biosynthetic process	0.006469276
c18	GO:0031326~regulation of cellular biosynthetic process	0.006726546
c18	GO:0051170~nuclear import	0.009699548
c18	GO:0044249~cellular biosynthetic process	0.012989794
c18	GO:0010608~posttranscriptional regulation of gene expression	0.013048015
c18	GO:0010556~regulation of macromolecule biosynthetic process	0.014912935
c18	GO:0043170~macromolecule metabolic process	0.015166062
c18	GO:0019222~regulation of metabolic process	0.015261899
c18	GO:0051171~regulation of nitrogen compound metabolic process	0.016267591
c18	GO:0008152~metabolic process	0.01653319
c18	GO:0060255~regulation of macromolecule metabolic process	0.017601004
c18	GO:0016071~mRNA metabolic process	0.017603184
c18	GO:0019219~regulation of nucleobase, nucleoside, nucleotide and nucleic acid metabolic process	0.020705566
c18	GO:0044237~cellular metabolic process	0.02392847
c18	GO:0006606~protein import into nucleus	0.024150689
c18	GO:0010467~gene expression	0.02595569
c18	GO:0031323~regulation of cellular metabolic process	0.026175127
c18	GO:0009058~biosynthetic process	0.027730643
c18	GO:0001701~in utero embryonic development	0.028657128
c18	GO:0080090~regulation of primary metabolic process	0.029951435
c18	GO:0045449~regulation of transcription	0.03185048
c18	GO:0046620~regulation of organ growth	0.032600207
c18	GO:0044260~cellular macromolecule metabolic process	0.036939646
c18	GO:0034641~cellular nitrogen compound metabolic process	0.037283257
c18	GO:0034504~protein localization in nucleus	0.038331625
c18	GO:0007623~circadian rhythm	0.038732561
c18	GO:0006397~mRNA processing	0.040612111
c18	GO:0017038~protein import	0.042268084
c18	GO:0042981~regulation of apoptosis	0.042846413
c18	GO:0043067~regulation of programmed cell death	0.04836099
c18	GO:0044238~primary metabolic process	0.048494612
c18	GO:0000060~protein import into nucleus, translocation	0.048606494

c18	GO:0006357~regulation of transcription from RNA polymerase II promoter	0.049253962
c18	GO:0001932~regulation of protein amino acid phosphorylation	0.051715557
c18	GO:0010941~regulation of cell death	0.052241199
c18	GO:0006807~nitrogen compound metabolic process	0.053479255
c18	GO:0051259~protein oligomerization	0.053589155
c18	GO:0006417~regulation of translation	0.05434459
c18	GO:0014706~striated muscle tissue development	0.054906741
c18	GO:0006913~nucleocytoplasmic transport	0.055248244
c18	GO:0016568~chromatin modification	0.058247087
c18	GO:0050730~regulation of peptidyl-tyrosine phosphorylation	0.058912051
c18	GO:0051169~nuclear transport	0.059470504
c18	GO:0007050~cell cycle arrest	0.059943507
c18	GO:0010460~positive regulation of heart rate	0.060385159
c18	GO:0034645~cellular macromolecule biosynthetic process	0.061731941
c18	GO:0006518~peptide metabolic process	0.064514183
c18	GO:0006350~transcription	0.067981232
c18	GO:0006139~nucleobase, nucleoside, nucleotide and nucleic acid metabolic process	0.068559634
c18	GO:0060537~muscle tissue development	0.070398949
c18	GO:0060043~regulation of cardiac muscle cell proliferation	0.071855781
c18	GO:0055024~regulation of cardiac muscle tissue development	0.071855781
c18	GO:0055021~regulation of cardiac muscle growth	0.071855781
c18	GO:0001892~embryonic placenta development	0.072783014
c18	GO:0051241~negative regulation of multicellular organismal process	0.073391012
c18	GO:0065003~macromolecular complex assembly	0.074315284
c18	GO:0009059~macromolecule biosynthetic process	0.074434032
c18	GO:0008380~RNA splicing	0.075137026
c18	GO:0009987~cellular process	0.075167394
c18	GO:0007049~cell cycle	0.075901072
c18	GO:0006333~chromatin assembly or disassembly	0.076111013
c18	GO:0022607~cellular component assembly	0.078043322
c18	GO:0032268~regulation of cellular protein metabolic process	0.078611039
c18	GO:0000122~negative regulation of transcription from RNA polymerase II promoter	0.079115934
c18	GO:0009082~branched chain family amino acid biosynthetic process	0.080083167
c18	GO:0007079~mitotic chromosome movement towards spindle pole	0.080083167
c18	GO:0001878~response to yeast	0.080083167
c18	GO:0044085~cellular component biogenesis	0.080592691
c18	GO:0060420~regulation of heart growth	0.083959097
c18	GO:0050766~positive regulation of phagocytosis	0.083959097
c18	GO:0016481~negative regulation of transcription	0.086958805
c18	GO:0050819~negative regulation of coagulation	0.088140158
c18	GO:0045766~positive regulation of angiogenesis	0.088140158
c18	GO:0043066~negative regulation of apoptosis	0.090202273
c18	GO:0051246~regulation of protein metabolic process	0.090665717
c18	GO:0042594~response to starvation	0.091313737
c18	GO:0010629~negative regulation of gene expression	0.09143723

c18	GO:0006396~RNA processing	0.091736437
c18	GO:0007517~muscle organ development	0.092563269
c18	GO:0040029~regulation of gene expression, epigenetic	0.094635768
c18	GO:0051276~chromosome organization	0.095724988
c18	GO:0006325~chromatin organization	0.095957941
c18	GO:0007093~mitotic cell cycle checkpoint	0.097600308
c18	GO:0007179~transforming growth factor beta receptor signaling pathway	0.097908193
c18	GO:0007612~learning	0.097908193
c18	GO:0001890~placenta development	0.097908193
c18	GO:0006464~protein modification process	0.09937637
c18	GO:0043069~negative regulation of programmed cell death	0.099512271
c19	GO:0019538~protein metabolic process	0.097667753
c20	GO:0044237~cellular metabolic process	4.26E-04
c20	GO:0044248~cellular catabolic process	0.002124524
c20	GO:0044265~cellular macromolecule catabolic process	0.002272588
c20	GO:0044238~primary metabolic process	0.002566543
c20	GO:0008152~metabolic process	0.003370082
c20	GO:0009987~cellular process	0.003920647
c20	GO:0009057~macromolecule catabolic process	0.004294953
c20	GO:0006807~nitrogen compound metabolic process	0.004489928
c20	GO:0043632~modification-dependent macromolecule catabolic process	0.005377021
c20	GO:0019941~modification-dependent protein catabolic process	0.005377021
c20	GO:0044260~cellular macromolecule metabolic process	0.005534394
c20	GO:0046907~intracellular transport	0.005647446
c20	GO:0009056~catabolic process	0.005760806
c20	GO:0006259~DNA metabolic process	0.007199538
c20	GO:0034641~cellular nitrogen compound metabolic process	0.00752771
c20	GO:0051603~proteolysis involved in cellular protein catabolic process	0.009444718
c20	GO:0006260~DNA replication	0.009798291
c20	GO:0016070~RNA metabolic process	0.009804622
c20	GO:0006139~nucleobase, nucleoside, nucleotide and nucleic acid metabolic process	0.009940175
c20	GO:0044257~cellular protein catabolic process	0.010272055
c20	GO:0048193~Golgi vesicle transport	0.014401886
c20	GO:0033554~cellular response to stress	0.014801325
c20	GO:0030163~protein catabolic process	0.015093785
c20	GO:0006986~response to unfolded protein	0.017140745
c20	GO:0043170~macromolecule metabolic process	0.019855943
c20	GO:0033043~regulation of organelle organization	0.024972271
c20	GO:0006261~DNA-dependent DNA replication	0.026497064
c20	GO:0051128~regulation of cellular component organization	0.027219521
c20	GO:0006396~RNA processing	0.030160299
c20	GO:0009636~response to toxin	0.032081462
c20	GO:0006968~cellular defense response	0.032081462
c20	GO:0009607~response to biotic stimulus	0.032583487
c20	GO:0051789~response to protein stimulus	0.036682968

c20	GO:0006694~steroid biosynthetic process	0.037463765
c20	GO:0051649~establishment of localization in cell	0.038626517
c20	GO:0006281~DNA repair	0.038681153
c20	GO:0006950~response to stress	0.041239741
c20	GO:0006754~ATP biosynthetic process	0.045269717
c20	GO:0006974~response to DNA damage stimulus	0.046026389
c20	GO:0032312~regulation of ARF GTPase activity	0.046872814
c20	GO:0034220~ion transmembrane transport	0.053753969
c20	GO:0034654~nucleobase, nucleoside, nucleotide and nucleic acid biosynthetic process	0.056151248
c20	GO:0034404~nucleobase, nucleoside and nucleotide biosynthetic process	0.056151248
c20	GO:0015031~protein transport	0.057616291
c20	GO:0042509~regulation of tyrosine phosphorylation of STAT protein	0.060378839
c20	GO:0045184~establishment of protein localization	0.061957049
c20	GO:0051216~cartilage development	0.064327396
c20	GO:0009206~purine ribonucleoside triphosphate biosynthetic process	0.066301874
c20	GO:0051641~cellular localization	0.068608062
c20	GO:0009201~ribonucleoside triphosphate biosynthetic process	0.068938755
c20	GO:0009145~purine nucleoside triphosphate biosynthetic process	0.068938755
c20	GO:0031047~gene silencing by RNA	0.070279086
c20	GO:0044419~interspecies interaction between organisms	0.071089079
c20	GO:0016064~immunoglobulin mediated immune response	0.071813118
c20	GO:0006091~generation of precursor metabolites and energy	0.074458752
c20	GO:0009142~nucleoside triphosphate biosynthetic process	0.077209045
c20	GO:0016192~vesicle-mediated transport	0.078266578
c20	GO:0019724~B cell mediated immunity	0.079800144
c20	GO:0006886~intracellular protein transport	0.080835177
c20	GO:0006984~ER-nuclear signaling pathway	0.080857664
c20	GO:0016126~sterol biosynthetic process	0.080857664
c20	GO:0008104~protein localization	0.082612201
c20	GO:0051712~positive regulation of killing of cells of another organism	0.084835
c20	GO:0043902~positive regulation of multi-organism process	0.084835
c20	GO:0046034~ATP metabolic process	0.086015437
c20	GO:0046425~regulation of JAK-STAT cascade	0.086390005
c20	GO:0006892~post-Golgi vesicle-mediated transport	0.088208235
c20	GO:0019752~carboxylic acid metabolic process	0.089064711
c20	GO:0043436~oxoacid metabolic process	0.089064711
c20	GO:0051169~nuclear transport	0.090813581
c20	GO:0044267~cellular protein metabolic process	0.091806876
c20	GO:0034613~cellular protein localization	0.093008914
c20	GO:0044271~nitrogen compound biosynthetic process	0.093999931
c20	GO:0009165~nucleotide biosynthetic process	0.094092464
c20	GO:0006082~organic acid metabolic process	0.094353242
c20	GO:0006352~transcription initiation	0.094382301
c20	GO:0070727~cellular macromolecule localization	0.099683773
c3	GO:0044264~cellular polysaccharide metabolic process	0.029652327

c3	GO:0043523~regulation of neuron apoptosis	0.049894244
c3	GO:0005976~polysaccharide metabolic process	0.061217732
c3	GO:0050804~regulation of synaptic transmission	0.074543968
c3	GO:0051969~regulation of transmission of nerve impulse	0.080354876
c3	GO:0031644~regulation of neurological system process	0.083510985
c21	GO:0006066~alcohol metabolic process	0.002718215
c21	GO:0044419~interspecies interaction between organisms	0.009161243
c21	GO:0006220~pyrimidine nucleotide metabolic process	0.015032505
c21	GO:0016125~sterol metabolic process	0.018485548
c21	GO:0006221~pyrimidine nucleotide biosynthetic process	0.019970522
c21	GO:0009987~cellular process	0.025833502
c21	GO:0044241~lipid digestion	0.027780691
c21	GO:0006213~pyrimidine nucleoside metabolic process	0.028129187
c21	GO:0051726~regulation of cell cycle	0.032463065
c21	GO:0000075~cell cycle checkpoint	0.034878886
c21	GO:0008203~cholesterol metabolic process	0.036508697
c21	GO:0016032~viral reproduction	0.041057448
c21	GO:0019752~carboxylic acid metabolic process	0.042594508
c21	GO:0043436~oxoacid metabolic process	0.042594508
c21	GO:0009798~axis specification	0.045061847
c21	GO:0006082~organic acid metabolic process	0.046019574
c21	GO:0006917~induction of apoptosis	0.048136251
c21	GO:0055065~metal ion homeostasis	0.048456132
c21	GO:0019319~hexose biosynthetic process	0.048935243
c21	GO:0012502~induction of programmed cell death	0.049465837
c21	GO:0007346~regulation of mitotic cell cycle	0.051316878
c21	GO:0042180~cellular ketone metabolic process	0.051454717
c21	GO:0055085~transmembrane transport	0.052951006
c21	GO:0009142~nucleoside triphosphate biosynthetic process	0.055499311
c21	GO:0044262~cellular carbohydrate metabolic process	0.05686717
c21	GO:0006874~cellular calcium ion homeostasis	0.057218388
c21	GO:0000279~M phase	0.057903393
c21	GO:0006511~ubiquitin-dependent protein catabolic process	0.0600246
c21	GO:0009165~nucleotide biosynthetic process	0.062104123
c21	GO:0007049~cell cycle	0.064916377
c21	GO:0055074~calcium ion homeostasis	0.065504525
c21	GO:0051336~regulation of hydrolase activity	0.068212939
c21	GO:0034654~nucleobase, nucleoside, nucleotide and nucleic acid biosynthetic process	0.073919533
c21	GO:0034404~nucleobase, nucleoside and nucleotide biosynthetic process	0.073919533
c21	GO:0046131~pyrimidine ribonucleoside metabolic process	0.074317597
c21	GO:0006000~fructose metabolic process	0.074317597
c21	GO:0046364~monosaccharide biosynthetic process	0.075377367
c21	GO:0001558~regulation of cell growth	0.075884144
c21	GO:0005975~carbohydrate metabolic process	0.076268927
c21	GO:0010742~foam cell differentiation	0.078006438

c21	GO:0006875~cellular metal ion homeostasis	0.079709849
c21	GO:0022415~viral reproductive process	0.083619647
c21	GO:0048878~chemical homeostasis	0.093135504
c21	GO:0006753~nucleoside phosphate metabolic process	0.093864748
c21	GO:0009117~nucleotide metabolic process	0.093864748
c22	GO:0009219~pyrimidine deoxyribonucleotide metabolic process	0.002949223
c22	GO:0044085~cellular component biogenesis	0.003321812
c22	GO:0009394~2'-deoxyribonucleotide metabolic process	0.009014286
c22	GO:0043933~macromolecular complex subunit organization	0.00937087
c22	GO:0009212~pyrimidine deoxyribonucleoside triphosphate biosynthetic process	0.013696534
c22	GO:0046075~dTTP metabolic process	0.013696534
c22	GO:0006235~dTTP biosynthetic process	0.013696534
c22	GO:0009262~deoxyribonucleotide metabolic process	0.014670017
c22	GO:0065003~macromolecular complex assembly	0.015856154
c22	GO:0009202~deoxyribonucleoside triphosphate biosynthetic process	0.020475012
c22	GO:0009221~pyrimidine deoxyribonucleotide biosynthetic process	0.020475012
c22	GO:0006461~protein complex assembly	0.02281534
c22	GO:0070271~protein complex biogenesis	0.02281534
c22	GO:0006220~pyrimidine nucleotide metabolic process	0.025301763
c22	GO:0009211~pyrimidine deoxyribonucleoside triphosphate metabolic process	0.027207381
c22	GO:0009265~2'-deoxyribonucleotide biosynthetic process	0.033893952
c22	GO:0006396~RNA processing	0.034422754
c22	GO:0022613~ribonucleoprotein complex biogenesis	0.035473052
c22	GO:0046125~pyrimidine deoxyribonucleoside metabolic process	0.040535032
c22	GO:0022607~cellular component assembly	0.040647017
c22	GO:0042254~ribosome biogenesis	0.051715454
c22	GO:0009120~deoxyribonucleoside metabolic process	0.053681946
c22	GO:0009263~deoxyribonucleotide biosynthetic process	0.053681946
c22	GO:0051168~nuclear export	0.063977744
c22	GO:0009200~deoxyribonucleoside triphosphate metabolic process	0.066650548
c22	GO:0009148~pyrimidine nucleoside triphosphate biosynthetic process	0.079443232
c22	GO:0009147~pyrimidine nucleoside triphosphate metabolic process	0.09206236
c24	GO:0055085~transmembrane transport	0.039776848
c24	GO:0009987~cellular process	0.072750553
c24	GO:0009308~amine metabolic process	0.07335037
c24	GO:0008544~epidermis development	0.084122328
c24	GO:0007283~spermatogenesis	0.094060152
c24	GO:0048232~male gamete generation	0.094060152
c25	GO:0044267~cellular protein metabolic process	0.069874038
c25	GO:0006464~protein modification process	0.075263024
c25	GO:0043412~biopolymer modification	0.084758092
c26	GO:0034404~nucleobase, nucleoside and nucleotide biosynthetic process	0.004237757
c26	GO:0034654~nucleobase, nucleoside, nucleotide and nucleic acid biosynthetic process	0.004237757
c26	GO:0009165~nucleotide biosynthetic process	0.00741059
c26	GO:0032535~regulation of cellular component size	0.009336933

c26	GO:0009152~purine ribonucleotide biosynthetic process	0.009559878
c26	GO:0051262~protein tetramerization	0.012416427
c26	GO:0009260~ribonucleotide biosynthetic process	0.014026761
c26	GO:0009615~response to virus	0.016739627
c26	GO:0006164~purine nucleotide biosynthetic process	0.018015136
c26	GO:0046483~heterocycle metabolic process	0.018317329
c26	GO:0055086~nucleobase, nucleoside and nucleotide metabolic process	0.02029548
c26	GO:0009206~purine ribonucleoside triphosphate biosynthetic process	0.024854122
c26	GO:0009145~purine nucleoside triphosphate biosynthetic process	0.026222657
c26	GO:0009201~ribonucleoside triphosphate biosynthetic process	0.026222657
c26	GO:0044271~nitrogen compound biosynthetic process	0.026294984
c26	GO:0009150~purine ribonucleotide metabolic process	0.027392453
c26	GO:0009142~nucleoside triphosphate biosynthetic process	0.030641097
c26	GO:0051707~response to other organism	0.033506119
c26	GO:0008361~regulation of cell size	0.035584777
c26	GO:0009117~nucleotide metabolic process	0.036502688
c26	GO:0006753~nucleoside phosphate metabolic process	0.036502688
c26	GO:0006163~purine nucleotide metabolic process	0.036749352
c26	GO:0009607~response to biotic stimulus	0.038054895
c26	GO:0007223~Wnt receptor signaling pathway, calcium modulating pathway	0.038067164
c26	GO:0006733~oxidoreduction coenzyme metabolic process	0.038436411
c26	GO:0034622~cellular macromolecular complex assembly	0.039332246
c26	GO:0009259~ribonucleotide metabolic process	0.039734728
c26	GO:0006754~ATP biosynthetic process	0.041171019
c26	GO:0051179~localization	0.043513007
c26	GO:0006814~sodium ion transport	0.04511946
c26	GO:0009112~nucleobase metabolic process	0.048097566
c26	GO:0065003~macromolecular complex assembly	0.057324803
c26	GO:0021544~subpallium development	0.057339385
c26	GO:0034621~cellular macromolecular complex subunit organization	0.058278905
c26	GO:0030317~sperm motility	0.059283505
c26	GO:0009205~purine ribonucleoside triphosphate metabolic process	0.060358106
c26	GO:0009199~ribonucleoside triphosphate metabolic process	0.06281742
c26	GO:0006888~ER to Golgi vesicle-mediated transport	0.063324746
c26	GO:0007338~single fertilization	0.064218738
c26	GO:0009566~fertilization	0.064817076
c26	GO:0007093~mitotic cell cycle checkpoint	0.067924884
c26	GO:0043623~cellular protein complex assembly	0.067929508
c26	GO:0016049~cell growth	0.067986438
c26	GO:0009987~cellular process	0.069323898
c26	GO:0032660~regulation of interleukin-17 production	0.070682419
c26	GO:0032740~positive regulation of interleukin-17 production	0.070682419
c26	GO:0009144~purine nucleoside triphosphate metabolic process	0.073273282
c26	GO:0006413~translational initiation	0.077626592
c26	GO:0043933~macromolecular complex subunit organization	0.079730952

c26	GO:0046034~ATP metabolic process	0.084372736
c26	GO:0046112~nucleobase biosynthetic process	0.088429
c26	GO:0001539~ciliary or flagellar motility	0.088429
c26	GO:0016051~carbohydrate biosynthetic process	0.091104296
c26	GO:0032649~regulation of interferon-gamma production	0.091920454
c26	GO:0045185~maintenance of protein location	0.093394601
c26	GO:0045793~positive regulation of cell size	0.093394601
c26	GO:0016192~vesicle-mediated transport	0.096477094
c26	GO:0006084~acetyl-CoA metabolic process	0.099180433
c28	GO:0032886~regulation of microtubule-based process	3.31E-04
c28	GO:0070507~regulation of microtubule cytoskeleton organization	6.85E-04
c28	GO:0006996~organelle organization	0.003142833
c28	GO:0006261~DNA-dependent DNA replication	0.004657509
c28	GO:0031110~regulation of microtubule polymerization or depolymerization	0.004672477
c28	GO:0033043~regulation of organelle organization	0.00543112
c28	GO:0042110~T cell activation	0.00584623
c28	GO:0006260~DNA replication	0.009012049
c28	GO:0006259~DNA metabolic process	0.009438366
c28	GO:0051276~chromosome organization	0.009770825
c28	GO:0031113~regulation of microtubule polymerization	0.010174496
c28	GO:0051493~regulation of cytoskeleton organization	0.010183954
c28	GO:0042692~muscle cell differentiation	0.012106723
c28	GO:0055001~muscle cell development	0.015079286
c28	GO:0031032~actomyosin structure organization	0.017214589
c28	GO:0008361~regulation of cell size	0.017333675
c28	GO:0051495~positive regulation of cytoskeleton organization	0.022290132
c28	GO:0045792~negative regulation of cell size	0.026491278
c28	GO:0007059~chromosome segregation	0.026688177
c28	GO:0006281~DNA repair	0.028673306
c28	GO:0006974~response to DNA damage stimulus	0.029466961
c28	GO:0031334~positive regulation of protein complex assembly	0.036179311
c28	GO:0032535~regulation of cellular component size	0.037032525
c28	GO:0006928~cell motion	0.037719607
c28	GO:0046928~regulation of neurotransmitter secretion	0.038256486
c28	GO:0030239~myofibril assembly	0.038256486
c28	GO:0051301~cell division	0.038670462
c28	GO:0055002~striated muscle cell development	0.03871197
c28	GO:0051146~striated muscle cell differentiation	0.039440373
c28	GO:0010927~cellular component assembly involved in morphogenesis	0.039583255
c28	GO:0016477~cell migration	0.042367383
c28	GO:0009987~cellular process	0.042738305
c28	GO:0016054~organic acid catabolic process	0.04733114
c28	GO:0046395~carboxylic acid catabolic process	0.04733114
c28	GO:0032401~establishment of melanosome localization	0.048205203
c28	GO:0042273~ribosomal large subunit biogenesis	0.048205203

c28	GO:0030308~negative regulation of cell growth	0.048259043
c28	GO:0001558~regulation of cell growth	0.048641327
c28	GO:0007498~mesoderm development	0.050411208
c28	GO:0006325~chromatin organization	0.056384823
c28	GO:0001775~cell activation	0.056578632
c28	GO:0051905~establishment of pigment granule localization	0.057541144
c28	GO:0032273~positive regulation of protein polymerization	0.059566358
c28	GO:0040017~positive regulation of locomotion	0.063676165
c28	GO:0051588~regulation of neurotransmitter transport	0.065601232
c28	GO:0014706~striated muscle tissue development	0.065932363
c28	GO:0031116~positive regulation of microtubule polymerization	0.067441544
c28	GO:0040008~regulation of growth	0.069025096
c28	GO:0046670~positive regulation of retinal cell programmed cell death	0.070819003
c28	GO:0048285~organelle fission	0.071607879
c28	GO:0050817~coagulation	0.075441434
c28	GO:0007596~blood coagulation	0.075441434
c28	GO:0006082~organic acid metabolic process	0.075718697
c28	GO:0031112~positive regulation of microtubule polymerization or depolymerization	0.077853735
c28	GO:0042375~quinone cofactor metabolic process	0.077853735
c28	GO:0032400~melanosome localization	0.077853735
c28	GO:0035051~cardiac cell differentiation	0.078466984
c28	GO:0010638~positive regulation of organelle organization	0.078893503
c28	GO:0060537~muscle tissue development	0.082483131
c28	GO:0055114~oxidation reduction	0.083167092
c28	GO:0042180~cellular ketone metabolic process	0.084459988
c28	GO:0043393~regulation of protein binding	0.085280721
c28	GO:0040012~regulation of locomotion	0.08783885
c28	GO:0051875~pigment granule localization	0.088728099
c28	GO:0051674~localization of cell	0.090039991
c28	GO:0048870~cell motility	0.090039991
c28	GO:0031497~chromatin assembly	0.093945309
c28	GO:0007599~hemostasis	0.095322297
c28	GO:0009063~cellular amino acid catabolic process	0.098289321
c28	GO:0006664~glycolipid metabolic process	0.099624299
c28	GO:0007049~cell cycle	0.099950562
c29	GO:0006839~mitochondrial transport	0.02298524
c29	GO:0016071~mRNA metabolic process	0.036578989
c29	GO:0006396~RNA processing	0.037365028
c29	GO:0009987~cellular process	0.05505438
c29	GO:0035137~hindlimb morphogenesis	0.084824677
c29	GO:0033365~protein localization in organelle	0.08806906
c29	GO:0000375~RNA splicing, via transesterification reactions	0.095334668
c29	GO:0000377~RNA splicing, via transesterification reactions with bulged adenosine as nucleophile	0.095334668
c29	GO:0000398~nuclear mRNA splicing, via spliceosome	0.095334668
c29	GO:0006397~mRNA processing	0.095456051

c29	GO:0016070~RNA metabolic process	0.096730525
c29	GO:0044265~cellular macromolecule catabolic process	0.098527966
c31	GO:0000165~MAPKKK cascade	0.005728136
c31	GO:0033674~positive regulation of kinase activity	0.008898542
c31	GO:0051347~positive regulation of transferase activity	0.009578386
c31	GO:0006793~phosphorus metabolic process	0.020026713
c31	GO:0006796~phosphate metabolic process	0.020026713
c31	GO:0043549~regulation of kinase activity	0.020414787
c31	GO:0007243~protein kinase cascade	0.02183638
c31	GO:0051338~regulation of transferase activity	0.022058759
c31	GO:0043170~macromolecule metabolic process	0.026715164
c31	GO:0043687~post-translational protein modification	0.033522442
c31	GO:0042325~regulation of phosphorylation	0.033569547
c31	GO:0019220~regulation of phosphate metabolic process	0.036136997
c31	GO:0051174~regulation of phosphorus metabolic process	0.036136997
c31	GO:0043085~positive regulation of catalytic activity	0.041066166
c31	GO:0044093~positive regulation of molecular function	0.051031413
c31	GO:0046777~protein amino acid autophosphorylation	0.052921316
c31	GO:0006464~protein modification process	0.056918505
c31	GO:0043412~biopolymer modification	0.064348435
c31	GO:0006468~protein amino acid phosphorylation	0.064369925
c31	GO:0044260~cellular macromolecule metabolic process	0.068962507
c31	GO:0044238~primary metabolic process	0.080718063
c31	GO:0016310~phosphorylation	0.088612267
c31	GO:0050790~regulation of catalytic activity	0.097595853
c32	GO:0006325~chromatin organization	2.91E-04
c32	GO:0051276~chromosome organization	0.002831571
c32	GO:0006396~RNA processing	0.002986598
c32	GO:0016568~chromatin modification	0.003423201
c32	GO:0044260~cellular macromolecule metabolic process	0.007082032
c32	GO:0022618~ribonucleoprotein complex assembly	0.007111888
c32	GO:0016570~histone modification	0.011388606
c32	GO:0006807~nitrogen compound metabolic process	0.01168306
c32	GO:0016569~covalent chromatin modification	0.0129583
c32	GO:0022613~ribonucleoprotein complex biogenesis	0.014924904
c32	GO:0016573~histone acetylation	0.015926225
c32	GO:0034641~cellular nitrogen compound metabolic process	0.015958923
c32	GO:0006473~protein amino acid acetylation	0.019711459
c32	GO:0043967~histone H4 acetylation	0.020238106
c32	GO:0006996~organelle organization	0.020753584
c32	GO:0043170~macromolecule metabolic process	0.021627978
c32	GO:0000056~ribosomal small subunit export from nucleus	0.021981222
c32	GO:0000054~ribosome export from nucleus	0.021981222
c32	GO:0033753~establishment of ribosome localization	0.021981222
c32	GO:0033750~ribosome localization	0.021981222

c32	GO:0006139~nucleobase, nucleoside, nucleotide and nucleic acid metabolic process	0.023089139
c32	GO:0010467~gene expression	0.023357887
c32	GO:0044237~cellular metabolic process	0.024965989
c32	GO:0008152~metabolic process	0.025794825
c32	GO:0006397~mRNA processing	0.026277584
c32	GO:0043543~protein amino acid acylation	0.028643002
c32	GO:0006915~apoptosis	0.034943963
c32	GO:0012501~programmed cell death	0.038456527
c32	GO:0000041~transition metal ion transport	0.043859729
c32	GO:0016032~viral reproduction	0.043859729
c32	GO:0006811~ion transport	0.045096667
c32	GO:0007049~cell cycle	0.048426705
c32	GO:0016071~mRNA metabolic process	0.05314981
c32	GO:0006259~DNA metabolic process	0.054273245
c32	GO:0051099~positive regulation of binding	0.055255844
c32	GO:0031016~pancreas development	0.056941098
c32	GO:0034097~response to cytokine stimulus	0.056988638
c32	GO:0048545~response to steroid hormone stimulus	0.062010473
c32	GO:0034622~cellular macromolecular complex assembly	0.063126029
c32	GO:0018346~protein amino acid prenylation	0.064513654
c32	GO:0006810~transport	0.064896993
c32	GO:0003002~regionalization	0.067575775
c32	GO:0009952~anterior/posterior pattern formation	0.069686744
c32	GO:0051234~establishment of localization	0.07334599
c32	GO:0018342~protein prenylation	0.07485638
c32	GO:0007389~pattern specification process	0.076009238
c32	GO:0007093~mitotic cell cycle checkpoint	0.081669207
c32	GO:0000375~RNA splicing, via transesterification reactions	0.089639011
c32	GO:0000377~RNA splicing, via transesterification reactions with bulged adenosine as nucleophile	0.089639011
c32	GO:0000398~nuclear mRNA splicing, via spliceosome	0.089639011
c32	GO:0044419~interspecies interaction between organisms	0.094094877
c32	GO:0018195~peptidyl-arginine modification	0.095202201
c32	GO:0016070~RNA metabolic process	0.095256807
c32	GO:0008380~RNA splicing	0.09529641
c32	GO:0022402~cell cycle process	0.096201595
c32	GO:0030324~lung development	0.096733333
c32	GO:0044238~primary metabolic process	0.099011325
c33	GO:0016310~phosphorylation	0.014740228
c33	GO:0006793~phosphorus metabolic process	0.034781752
c33	GO:0006796~phosphate metabolic process	0.034781752
c33	GO:0044265~cellular macromolecule catabolic process	0.035536246
c33	GO:0044248~cellular catabolic process	0.04304338
c33	GO:0009057~macromolecule catabolic process	0.046585219
c33	GO:0051603~proteolysis involved in cellular protein catabolic process	0.065084152
c33	GO:0044267~cellular protein metabolic process	0.065325152

c33	GO:0044257~cellular protein catabolic process	0.066045097
c33	GO:0030163~protein catabolic process	0.072306326
c33	GO:0019538~protein metabolic process	0.086628917
c33	GO:0006468~protein amino acid phosphorylation	0.088322071
c33	GO:0009056~catabolic process	0.094940919
c33	GO:0032507~maintenance of protein location in cell	0.099535528
c34	GO:0051656~establishment of organelle localization	0.001322379
c34	GO:0051640~organelle localization	0.00225797
c34	GO:0009987~cellular process	0.011982412
c34	GO:0016070~RNA metabolic process	0.017628116
c34	GO:0006396~RNA processing	0.022117483
c34	GO:0009056~catabolic process	0.029470694
c34	GO:0006914~autophagy	0.029810203
c34	GO:0006903~vesicle targeting	0.02983775
c34	GO:0043623~cellular protein complex assembly	0.030509712
c34	GO:0044237~cellular metabolic process	0.035235467
c34	GO:0007379~segment specification	0.044262692
c34	GO:0031398~positive regulation of protein ubiquitination	0.047062796
c34	GO:0008152~metabolic process	0.047558939
c34	GO:0034621~cellular macromolecular complex subunit organization	0.055538018
c34	GO:0044248~cellular catabolic process	0.057154666
c34	GO:0051437~positive regulation of ubiquitin-protein ligase activity during mitotic cell cycle	0.060618906
c34	GO:0006813~potassium ion transport	0.06308495
c34	GO:0051443~positive regulation of ubiquitin-protein ligase activity	0.066999373
c34	GO:0051439~regulation of ubiquitin-protein ligase activity during mitotic cell cycle	0.070324496
c34	GO:0006461~protein complex assembly	0.070385642
c34	GO:0070271~protein complex biogenesis	0.070385642
c34	GO:0043112~receptor metabolic process	0.071056917
c34	GO:0034622~cellular macromolecular complex assembly	0.073409279
c34	GO:0051297~centrosome organization	0.076670687
c34	GO:0006511~ubiquitin-dependent protein catabolic process	0.076805221
c34	GO:0051351~positive regulation of ligase activity	0.077242374
c34	GO:0051188~cofactor biosynthetic process	0.082393448
c34	GO:0051650~establishment of vesicle localization	0.082472912
c34	GO:0051649~establishment of localization in cell	0.082877419
c34	GO:0051186~cofactor metabolic process	0.084654545
c34	GO:0006400~tRNA modification	0.087255881
c34	GO:0000226~microtubule cytoskeleton organization	0.08910274
c34	GO:0046502~uroporphyrinogen III metabolic process	0.09063265
c34	GO:0031396~regulation of protein ubiquitination	0.092189915
c34	GO:0031023~microtubule organizing center organization	0.094619435
c34	GO:0051438~regulation of ubiquitin-protein ligase activity	0.096069041
c34	GO:0007017~microtubule-based process	0.098517996
c35	GO:0009308~amine metabolic process	0.026267068
c35	GO:0006950~response to stress	0.048412461

c35	GO:0002376~immune system process	0.075064563
c35	GO:0006955~immune response	0.099748906
c36	GO:0034984~cellular response to DNA damage stimulus	0.017725832
c36	GO:0048232~male gamete generation	0.017905798
c36	GO:0007283~spermatogenesis	0.017905798
c36	GO:0055114~oxidation reduction	0.034599919
c36	GO:0007276~gamete generation	0.034603734
c36	GO:0019953~sexual reproduction	0.036824895
c36	GO:0016070~RNA metabolic process	0.040794488
c36	GO:0022414~reproductive process	0.051882712
c36	GO:0048706~embryonic skeletal system development	0.053874655
c36	GO:0007286~spermatid development	0.054869802
c36	GO:0000003~reproduction	0.054986398
c36	GO:0060271~cilium morphogenesis	0.056419425
c36	GO:0031047~gene silencing by RNA	0.056419425
c36	GO:0010876~lipid localization	0.05835444
c36	GO:0048562~embryonic organ morphogenesis	0.064347038
c36	GO:0048704~embryonic skeletal system morphogenesis	0.06450165
c36	GO:0048515~spermatid differentiation	0.06450165
c36	GO:0006760~folic acid and derivative metabolic process	0.066098134
c36	GO:0044255~cellular lipid metabolic process	0.068694555
c36	GO:0043473~pigmentation	0.071381442
c36	GO:0010043~response to zinc ion	0.073632834
c36	GO:0009887~organ morphogenesis	0.074719579
c36	GO:0008594~photoreceptor cell morphogenesis	0.077603703
c36	GO:0046655~folic acid metabolic process	0.077603703
c36	GO:0048705~skeletal system morphogenesis	0.077944586
c36	GO:0055092~sterol homeostasis	0.079300406
c36	GO:0042632~cholesterol homeostasis	0.079300406
c36	GO:0019438~aromatic compound biosynthetic process	0.081426657
c36	GO:0030005~cellular di-, tri-valent inorganic cation homeostasis	0.081551967
c36	GO:0009987~cellular process	0.082267549
c36	GO:0048568~embryonic organ development	0.088324248
c36	GO:0006351~transcription, DNA-dependent	0.090103052
c36	GO:0006869~lipid transport	0.091965041
c36	GO:0006629~lipid metabolic process	0.096684811
c36	GO:0046942~carboxylic acid transport	0.097126382
c36	GO:0032774~RNA biosynthetic process	0.097136384
c36	GO:0030216~keratinocyte differentiation	0.098233544
c36	GO:0048609~reproductive process in a multicellular organism	0.099362173
c36	GO:0032504~multicellular organism reproduction	0.099362173
c36	GO:0015849~organic acid transport	0.099765989
c37	GO:0007399~nervous system development	0.058545175
c37	GO:0060255~regulation of macromolecule metabolic process	0.06573493
c37	GO:0006996~organelle organization	0.070687948

c37	GO:0007417~central nervous system development	0.083333355
c37	GO:0019222~regulation of metabolic process	0.089848271
c37	GO:0016568~chromatin modification	0.09097445
c37	GO:0016579~protein deubiquitination	0.096601503
c38	GO:0008152~metabolic process	3.31E-04
c38	GO:0044238~primary metabolic process	0.003663124
c38	GO:0008624~induction of apoptosis by extracellular signals	0.00495627
c38	GO:0006259~DNA metabolic process	0.01410406
c38	GO:0006917~induction of apoptosis	0.016994364
c38	GO:0034641~cellular nitrogen compound metabolic process	0.017374885
c38	GO:0012502~induction of programmed cell death	0.017410812
c38	GO:0043170~macromolecule metabolic process	0.020995019
c38	GO:0006139~nucleobase, nucleoside, nucleotide and nucleic acid metabolic process	0.022725884
c38	GO:0006807~nitrogen compound metabolic process	0.025202212
c38	GO:0009987~cellular process	0.028172932
c38	GO:0043065~positive regulation of apoptosis	0.030919679
c38	GO:0044237~cellular metabolic process	0.032011968
c38	GO:0043068~positive regulation of programmed cell death	0.032509135
c38	GO:0010942~positive regulation of cell death	0.033473743
c38	GO:0051225~spindle assembly	0.03912565
c38	GO:0006886~intracellular protein transport	0.047421371
c38	GO:0007051~spindle organization	0.051152783
c38	GO:0055114~oxidation reduction	0.053357079
c38	GO:0009119~ribonucleoside metabolic process	0.053995415
c38	GO:0009301~snRNA transcription	0.054862922
c38	GO:0044260~cellular macromolecule metabolic process	0.056346205
c38	GO:0006915~apoptosis	0.058968545
c38	GO:0012501~programmed cell death	0.065603837
c38	GO:0005975~carbohydrate metabolic process	0.071415249
c38	GO:0008219~cell death	0.078681626
c38	GO:0016265~death	0.083121291
c38	GO:0034613~cellular protein localization	0.084624672
c38	GO:0000279~M phase	0.088422396
c38	GO:0070727~cellular macromolecule localization	0.088543349
c38	GO:0022417~protein maturation by protein folding	0.089761752
c39	GO:0006936~muscle contraction	0.028440536
c39	GO:0006913~nucleocytoplasmic transport	0.02988689
c39	GO:0051169~nuclear transport	0.030872479
c39	GO:0003012~muscle system process	0.036055446
c39	GO:0051028~mRNA transport	0.054371745
c39	GO:0046907~intracellular transport	0.063524635
c39	GO:0050657~nucleic acid transport	0.065858759
c39	GO:0051236~establishment of RNA localization	0.065858759
c39	GO:0050658~RNA transport	0.065858759
c39	GO:0009987~cellular process	0.067539011

c39	GO:0006403~RNA localization	0.069450899
c39	GO:0007155~cell adhesion	0.08083734
c39	GO:0022610~biological adhesion	0.081269092
c39	GO:0015931~nucleobase, nucleoside, nucleotide and nucleic acid transport	0.08572202
c39	GO:0033036~macromolecule localization	0.090609842
c39	GO:0006942~regulation of striated muscle contraction	0.090942134
c39	GO:0019752~carboxylic acid metabolic process	0.091698428
c39	GO:0043436~oxoacid metabolic process	0.091698428
c39	GO:0006082~organic acid metabolic process	0.09384829
c39	GO:0042180~cellular ketone metabolic process	0.09767352
c5	GO:0000904~cell morphogenesis involved in differentiation	4.67E-04
c5	GO:0048468~cell development	0.001460091
c5	GO:0000902~cell morphogenesis	0.002511812
c5	GO:0032989~cellular component morphogenesis	0.004010928
c5	GO:0030154~cell differentiation	0.009570533
c5	GO:0048869~cellular developmental process	0.012658853
c5	GO:0009653~anatomical structure morphogenesis	0.012856652
c5	GO:0007409~axonogenesis	0.014942403
c5	GO:0048699~generation of neurons	0.016312987
c5	GO:0048667~cell morphogenesis involved in neuron differentiation	0.018448684
c5	GO:0048812~neuron projection morphogenesis	0.019391004
c5	GO:0022008~neurogenesis	0.021623489
c5	GO:0007399~nervous system development	0.024282732
c5	GO:0007417~central nervous system development	0.026686355
c5	GO:0048858~cell projection morphogenesis	0.027883044
c5	GO:0032990~cell part morphogenesis	0.031193845
c5	GO:0031175~neuron projection development	0.031193845
c5	GO:0006928~cell motion	0.037922148
c5	GO:0045685~regulation of glial cell differentiation	0.052519173
c5	GO:0014013~regulation of gliogenesis	0.055073392
c5	GO:0048666~neuron development	0.0624697
c5	GO:0032502~developmental process	0.06291221
c5	GO:0045941~positive regulation of transcription	0.06381343
c5	GO:0048856~anatomical structure development	0.064537948
c5	GO:0010628~positive regulation of gene expression	0.069613814
c5	GO:0030030~cell projection organization	0.075883179
c5	GO:0048731~system development	0.084245966
c5	GO:0045935~positive regulation of nucleobase, nucleoside, nucleotide and nucleic acid metabolic process	0.085475026
c5	GO:0042060~wound healing	0.093294222
c5	GO:0051173~positive regulation of nitrogen compound metabolic process	0.093418536
c5	GO:0006355~regulation of transcription, DNA-dependent	0.096080371
c5	GO:0010557~positive regulation of macromolecule biosynthetic process	0.097521299
c40	GO:0022604~regulation of cell morphogenesis	0.007574933
c40	GO:0050770~regulation of axonogenesis	0.007625169
c40	GO:0046622~positive regulation of organ growth	0.009095131

c40	GO:0051262~protein tetramerization	0.009696595
c40	GO:0010975~regulation of neuron projection development	0.017593507
c40	GO:0009396~folic acid and derivative biosynthetic process	0.018673346
c40	GO:0009987~cellular process	0.022953202
c40	GO:0010769~regulation of cell morphogenesis involved in differentiation	0.025457694
c40	GO:0030705~cytoskeleton-dependent intracellular transport	0.025689069
c40	GO:0046620~regulation of organ growth	0.026365332
c40	GO:0048519~negative regulation of biological process	0.027873653
c40	GO:0006508~proteolysis	0.032128923
c40	GO:0043170~macromolecule metabolic process	0.037754343
c40	GO:0009076~histidine family amino acid biosynthetic process	0.042748024
c40	GO:0000105~histidine biosynthetic process	0.042748024
c40	GO:0031344~regulation of cell projection organization	0.04354786
c40	GO:0006760~folic acid and derivative metabolic process	0.045744446
c40	GO:0000278~mitotic cell cycle	0.059213313
c40	GO:0009108~coenzyme biosynthetic process	0.061984627
c40	GO:0032536~regulation of cell projection size	0.063433999
c40	GO:0031133~regulation of axon diameter	0.063433999
c40	GO:0006915~apoptosis	0.063951379
c40	GO:0033554~cellular response to stress	0.065922948
c40	GO:0032570~response to progesterone stimulus	0.068481465
c40	GO:0045664~regulation of neuron differentiation	0.068653728
c40	GO:0050767~regulation of neurogenesis	0.068859958
c40	GO:0002252~immune effector process	0.070637355
c40	GO:0012501~programmed cell death	0.072009297
c40	GO:0051329~interphase of mitotic cell cycle	0.072419285
c40	GO:0043632~modification-dependent macromolecule catabolic process	0.072980618
c40	GO:0019941~modification-dependent protein catabolic process	0.072980618
c40	GO:0050804~regulation of synaptic transmission	0.074703025
c40	GO:0016043~cellular component organization	0.076387658
c40	GO:0007049~cell cycle	0.077248508
c40	GO:0044260~cellular macromolecule metabolic process	0.078406322
c40	GO:0051325~interphase	0.079697716
c40	GO:0051716~cellular response to stimulus	0.080512636
c40	GO:0019538~protein metabolic process	0.080826532
c40	GO:0006259~DNA metabolic process	0.0834546
c40	GO:0045110~intermediate filament bundle assembly	0.083674389
c40	GO:0030048~actin filament-based movement	0.087459958
c40	GO:0048523~negative regulation of cellular process	0.087725267
c40	GO:0006281~DNA repair	0.0892212
c40	GO:0006730~one-carbon metabolic process	0.0953781
c40	GO:0006996~organelle organization	0.095616985
c40	GO:0042493~response to drug	0.0980726
c40	GO:0051969~regulation of transmission of nerve impulse	0.099382915
c41	GO:0008219~cell death	0.021135237

c41	GO:0016265~death	0.022055546
c41	GO:0032502~developmental process	0.033871301
c41	GO:0008380~RNA splicing	0.041091575
c41	GO:0006915~apoptosis	0.047199288
c41	GO:0012501~programmed cell death	0.050697347
c41	GO:0007275~multicellular organismal development	0.053032267
c41	GO:0035162~embryonic hemopoiesis	0.05837628
c41	GO:0035295~tube development	0.059244353
c41	GO:0006397~mRNA processing	0.063081287
c41	GO:0043009~chordate embryonic development	0.069979488
c41	GO:0009792~embryonic development ending in birth or egg hatching	0.072127752
c41	GO:0043412~biopolymer modification	0.07934514
c41	GO:0045086~positive regulation of interleukin-2 biosynthetic process	0.083226665
c41	GO:0009651~response to salt stress	0.089337187
c42	GO:0051179~localization	0.005147009
c42	GO:0006091~generation of precursor metabolites and energy	0.012805722
c42	GO:0044262~cellular carbohydrate metabolic process	0.015011432
c42	GO:0051234~establishment of localization	0.016334523
c42	GO:0006810~transport	0.022971334
c42	GO:0005975~carbohydrate metabolic process	0.038203115
c42	GO:0042060~wound healing	0.041626849
c42	GO:0019318~hexose metabolic process	0.042412633
c42	GO:0019752~carboxylic acid metabolic process	0.050001232
c42	GO:0043436~oxoacid metabolic process	0.050001232
c42	GO:0007584~response to nutrient	0.050485603
c42	GO:0046364~monosaccharide biosynthetic process	0.051897115
c42	GO:0006082~organic acid metabolic process	0.052011886
c42	GO:0044275~cellular carbohydrate catabolic process	0.052386241
c42	GO:0042180~cellular ketone metabolic process	0.055659529
c42	GO:0006754~ATP biosynthetic process	0.058565245
c42	GO:0006164~purine nucleotide biosynthetic process	0.05973553
c42	GO:0006090~pyruvate metabolic process	0.064996138
c42	GO:0006006~glucose metabolic process	0.065882358
c42	GO:0005996~monosaccharide metabolic process	0.070170078
c42	GO:0009056~catabolic process	0.070283053
c42	GO:0046165~alcohol biosynthetic process	0.070514419
c42	GO:0009206~purine ribonucleoside triphosphate biosynthetic process	0.073651013
c42	GO:0009145~purine nucleoside triphosphate biosynthetic process	0.075424656
c42	GO:0009201~ribonucleoside triphosphate biosynthetic process	0.075424656
c42	GO:0007596~blood coagulation	0.08085815
c42	GO:0009142~nucleoside triphosphate biosynthetic process	0.08085815
c42	GO:0050817~coagulation	0.08085815
c42	GO:0034220~ion transmembrane transport	0.084935821
c42	GO:0015991~ATP hydrolysis coupled proton transport	0.085823441
c42	GO:0015988~energy coupled proton transport, against electrochemical gradient	0.085823441

c42	GO:0046034~ATP metabolic process	0.086456781
c42	GO:0016051~carbohydrate biosynthetic process	0.090278531
c42	GO:0007599~hemostasis	0.092215651
c42	GO:0006511~ubiquitin-dependent protein catabolic process	0.093135589
c42	GO:0016052~carbohydrate catabolic process	0.094170017
c42	GO:0006812~cation transport	0.099026298
c43	GO:0009051~pentose-phosphate shunt, oxidative branch	0.007918801
c43	GO:0031400~negative regulation of protein modification process	0.011705813
c43	GO:0019322~pentose biosynthetic process	0.011855073
c43	GO:0032269~negative regulation of cellular protein metabolic process	0.034529037
c43	GO:0006098~pentose-phosphate shunt	0.035152611
c43	GO:0051248~negative regulation of protein metabolic process	0.038002964
c43	GO:0019321~pentose metabolic process	0.042797911
c43	GO:0010638~positive regulation of organelle organization	0.042870683
c43	GO:0006897~endocytosis	0.05667531
c43	GO:0010324~membrane invagination	0.05667531
c43	GO:0006739~NADP metabolic process	0.06165181
c43	GO:0016192~vesicle-mediated transport	0.077463781
c43	GO:0034613~cellular protein localization	0.079800144
c43	GO:0070727~cellular macromolecule localization	0.081466003
c43	GO:0007049~cell cycle	0.085825985
c43	GO:0045840~positive regulation of mitosis	0.094674047
c43	GO:0051785~positive regulation of nuclear division	0.094674047
c44	GO:0009987~cellular process	0.009638323
c44	GO:0010035~response to inorganic substance	0.009985144
c44	GO:0006970~response to osmotic stress	0.01043209
c44	GO:0030258~lipid modification	0.010798969
c44	GO:0044237~cellular metabolic process	0.01142384
c44	GO:0022414~reproductive process	0.015521073
c44	GO:0000003~reproduction	0.016902733
c44	GO:0006875~cellular metal ion homeostasis	0.017856257
c44	GO:0065008~regulation of biological quality	0.017965005
c44	GO:0007283~spermatogenesis	0.020479278
c44	GO:0048232~male gamete generation	0.020479278
c44	GO:0007276~gamete generation	0.022253869
c44	GO:0055065~metal ion homeostasis	0.023773359
c44	GO:0010038~response to metal ion	0.023988399
c44	GO:0019953~sexual reproduction	0.024512285
c44	GO:0008152~metabolic process	0.026362499
c44	GO:0006874~cellular calcium ion homeostasis	0.027333518
c44	GO:0044093~positive regulation of molecular function	0.028705239
c44	GO:0007204~elevation of cytosolic calcium ion concentration	0.029766474
c44	GO:0007292~female gamete generation	0.03107243
c44	GO:0006950~response to stress	0.03125538
c44	GO:0044260~cellular macromolecule metabolic process	0.031417481

c44	GO:0055074~calcium ion homeostasis	0.032071275
c44	GO:0048640~negative regulation of developmental growth	0.034182833
c44	GO:0050790~regulation of catalytic activity	0.034888671
c44	GO:0051480~cytosolic calcium ion homeostasis	0.041201913
c44	GO:0042221~response to chemical stimulus	0.041548072
c44	GO:0032504~multicellular organism reproduction	0.04244421
c44	GO:0048609~reproductive process in a multicellular organism	0.04244421
c44	GO:0006631~fatty acid metabolic process	0.043426326
c44	GO:0030003~cellular cation homeostasis	0.04376012
c44	GO:0043085~positive regulation of catalytic activity	0.044849547
c44	GO:0044238~primary metabolic process	0.046854825
c44	GO:0050864~regulation of B cell activation	0.048736665
c44	GO:0043162~ubiquitin-dependent protein catabolic process via the multivesicular body sorting pathway	0.053392522
c44	GO:0001933~negative regulation of protein amino acid phosphorylation	0.05469287
c44	GO:0016125~sterol metabolic process	0.056206456
c44	GO:0006936~muscle contraction	0.056348967
c44	GO:0031399~regulation of protein modification process	0.058136136
c44	GO:0007032~endosome organization	0.060803468
c44	GO:0001893~maternal placenta development	0.060803468
c44	GO:0051592~response to calcium ion	0.06124414
c44	GO:0044248~cellular catabolic process	0.062782942
c44	GO:0032787~monocarboxylic acid metabolic process	0.066170569
c44	GO:0009060~aerobic respiration	0.068074014
c44	GO:0048738~cardiac muscle tissue development	0.068074683
c44	GO:0045446~endothelial cell differentiation	0.068279421
c44	GO:0043170~macromolecule metabolic process	0.070234046
c44	GO:0010648~negative regulation of cell communication	0.074382453
c44	GO:0051241~negative regulation of multicellular organismal process	0.077593019
c44	GO:0006979~response to oxidative stress	0.077593019
c44	GO:0031214~biomineral formation	0.07773956
c44	GO:0006925~inflammatory cell apoptosis	0.079012761
c44	GO:0043543~protein amino acid acylation	0.07902368
c44	GO:0044255~cellular lipid metabolic process	0.079523772
c44	GO:0006694~steroid biosynthetic process	0.080437237
c44	GO:0007188~G-protein signaling, coupled to cAMP nucleotide second messenger	0.080437237
c44	GO:0009605~response to external stimulus	0.081239444
c44	GO:0042325~regulation of phosphorylation	0.082633789
c44	GO:0006873~cellular ion homeostasis	0.083675825
c44	GO:0010033~response to organic substance	0.083927641
c44	GO:0050869~negative regulation of B cell activation	0.084061057
c44	GO:0003012~muscle system process	0.086363325
c44	GO:0042129~regulation of T cell proliferation	0.08677984
c44	GO:0055080~cation homeostasis	0.088650389
c44	GO:0044267~cellular protein metabolic process	0.088803993
c44	GO:0032844~regulation of homeostatic process	0.089092508

c44	GO:0030005~cellular di-, tri-valent inorganic cation homeostasis	0.089163992
c44	GO:0042180~cellular ketone metabolic process	0.091742796
c44	GO:0055082~cellular chemical homeostasis	0.092685941
c44	GO:0007033~vacuole organization	0.093283431
c44	GO:0032268~regulation of cellular protein metabolic process	0.093780177
c44	GO:0030154~cell differentiation	0.094007218
c44	GO:0001932~regulation of protein amino acid phosphorylation	0.09810818
c46	GO:0044248~cellular catabolic process	0.004857685
c46	GO:0009310~amine catabolic process	0.005972145
c46	GO:0002260~lymphocyte homeostasis	0.00797942
c46	GO:0051128~regulation of cellular component organization	0.008341762
c46	GO:0001776~leukocyte homeostasis	0.015953583
c46	GO:0009056~catabolic process	0.018228106
c46	GO:0051656~establishment of organelle localization	0.019101241
c46	GO:0051303~establishment of chromosome localization	0.020180455
c46	GO:0042402~biogenic amine catabolic process	0.020180455
c46	GO:0050000~chromosome localization	0.020180455
c46	GO:0048285~organelle fission	0.020770626
c46	GO:0006917~induction of apoptosis	0.020971918
c46	GO:0012502~induction of programmed cell death	0.021411263
c46	GO:0030901~midbrain development	0.022841385
c46	GO:0042219~cellular amino acid derivative catabolic process	0.031620389
c46	GO:0032989~cellular component morphogenesis	0.034481534
c46	GO:0009308~amine metabolic process	0.036073861
c46	GO:0006519~cellular amino acid and derivative metabolic process	0.037121134
c46	GO:0043029~T cell homeostasis	0.038092244
c46	GO:0042180~cellular ketone metabolic process	0.042662102
c46	GO:0022603~regulation of anatomical structure morphogenesis	0.044534221
c46	GO:0021575~hindbrain morphogenesis	0.045019951
c46	GO:0051640~organelle localization	0.047655463
c46	GO:0001101~response to acid	0.048644655
c46	GO:0043065~positive regulation of apoptosis	0.055490534
c46	GO:0043068~positive regulation of programmed cell death	0.057655411
c46	GO:0010942~positive regulation of cell death	0.059665039
c46	GO:0051129~negative regulation of cellular component organization	0.060009416
c46	GO:0009987~cellular process	0.06302463
c46	GO:0001775~cell activation	0.063781544
c46	GO:0045321~leukocyte activation	0.068957489
c46	GO:0032879~regulation of localization	0.069511218
c46	GO:0019752~carboxylic acid metabolic process	0.070075564
c46	GO:0043436~oxoacid metabolic process	0.070075564
c46	GO:0043627~response to estrogen stimulus	0.07053785
c46	GO:0006401~RNA catabolic process	0.071710565
c46	GO:0032801~receptor catabolic process	0.071879426
c46	GO:0019987~negative regulation of anti-apoptosis	0.071879426

c46	GO:0051301~cell division	0.072486896
c46	GO:0006082~organic acid metabolic process	0.073207237
c46	GO:0032787~monocarboxylic acid metabolic process	0.078385438
c46	GO:0051704~multi-organism process	0.081092481
c46	GO:0016054~organic acid catabolic process	0.082693186
c46	GO:0046395~carboxylic acid catabolic process	0.082693186
c46	GO:0044106~cellular amine metabolic process	0.084132749
c46	GO:0008624~induction of apoptosis by extracellular signals	0.084813446
c46	GO:0050871~positive regulation of B cell activation	0.085422782
c46	GO:0050678~regulation of epithelial cell proliferation	0.088116603
c46	GO:0042981~regulation of apoptosis	0.088269585
c46	GO:0051223~regulation of protein transport	0.089133157
c46	GO:0042221~response to chemical stimulus	0.093179479
c46	GO:0043067~regulation of programmed cell death	0.09445663
c46	GO:0045216~cell-cell junction organization	0.094460932
c46	GO:0016126~sterol biosynthetic process	0.094460932
c46	GO:0000956~nuclear-transcribed mRNA catabolic process	0.094460932
c46	GO:0007517~muscle organ development	0.094878908
c46	GO:0010941~regulation of cell death	0.096846649
c46	GO:0000070~mitotic sister chromatid segregation	0.099079012
c6	GO:0050905~neuromuscular process	0.006996791
c6	GO:0007154~cell communication	0.008778861
c6	GO:0050885~neuromuscular process controlling balance	0.008880237
c6	GO:0007265~Ras protein signal transduction	0.00941394
c6	GO:0048701~embryonic cranial skeleton morphogenesis	0.020087847
c6	GO:0007267~cell-cell signaling	0.033312813
c6	GO:0010817~regulation of hormone levels	0.038286864
c6	GO:0009888~tissue development	0.065302806
c6	GO:0006032~chitin catabolic process	0.082715564
c6	GO:0006030~chitin metabolic process	0.082715564
c6	GO:0007398~ectoderm development	0.098699788
c48	GO:0051240~positive regulation of multicellular organismal process	0.00289951
c48	GO:0051239~regulation of multicellular organismal process	0.004028153
c48	GO:0044057~regulation of system process	0.005625005
c48	GO:0051049~regulation of transport	0.014326991
c48	GO:0051241~negative regulation of multicellular organismal process	0.016272746
c48	GO:0051046~regulation of secretion	0.024063594
c48	GO:0032879~regulation of localization	0.034745241
c48	GO:0046903~secretion	0.049628807
c48	GO:0051179~localization	0.050032242
c48	GO:0048518~positive regulation of biological process	0.085767337
c48	GO:0065008~regulation of biological quality	0.092667881
c49	GO:0032312~regulation of ARF GTPase activity	0.005469013
c49	GO:0032012~regulation of ARF protein signal transduction	0.013143858
c49	GO:0043687~post-translational protein modification	0.016747945

c49	GO:0006464~protein modification process	0.025696953
c49	GO:0051656~establishment of organelle localization	0.030608367
c49	GO:0007080~mitotic metaphase plate congression	0.035152611
c49	GO:0043412~biopolymer modification	0.035254987
c49	GO:0065009~regulation of molecular function	0.036278598
c49	GO:0051310~metaphase plate congression	0.042797911
c49	GO:0051336~regulation of hydrolase activity	0.044423651
c49	GO:0006468~protein amino acid phosphorylation	0.04779856
c49	GO:0050790~regulation of catalytic activity	0.048802948
c49	GO:0051640~organelle localization	0.051564428
c49	GO:0050000~chromosome localization	0.057910443
c49	GO:0051303~establishment of chromosome localization	0.057910443
c49	GO:0032318~regulation of Ras GTPase activity	0.064037615
c49	GO:0031324~negative regulation of cellular metabolic process	0.064629838
c49	GO:0010605~negative regulation of macromolecule metabolic process	0.06959953
c49	GO:0006996~organelle organization	0.077518052
c49	GO:0043087~regulation of GTPase activity	0.085587489
c49	GO:0009892~negative regulation of metabolic process	0.087473962
c49	GO:0000122~negative regulation of transcription from RNA polymerase II promoter	0.088615254
c49	GO:0006796~phosphate metabolic process	0.088736907
c49	GO:0006793~phosphorus metabolic process	0.088736907
c49	GO:0016310~phosphorylation	0.09597891
c49	GO:0042981~regulation of apoptosis	0.0977326
c50	GO:0006396~RNA processing	0.022237816
c50	GO:0060341~regulation of cellular localization	0.031371825
c50	GO:0007155~cell adhesion	0.04196181
c50	GO:0022610~biological adhesion	0.042112938
c50	GO:0006397~mRNA processing	0.050150065
c50	GO:0016071~mRNA metabolic process	0.064555389
c50	GO:0032386~regulation of intracellular transport	0.077550023
c50	GO:0006937~regulation of muscle contraction	0.078600874
c50	GO:0051049~regulation of transport	0.085560005
c50	GO:0016070~RNA metabolic process	0.085694734
c51	GO:0006950~response to stress	0.002874949
c51	GO:0051704~multi-organism process	0.003059601
c51	GO:0009607~response to biotic stimulus	0.004456274
c51	GO:0043623~cellular protein complex assembly	0.009455976
c51	GO:0051641~cellular localization	0.010202422
c51	GO:0051258~protein polymerization	0.011817717
c51	GO:0051649~establishment of localization in cell	0.012054526
c51	GO:0055114~oxidation reduction	0.012432668
c51	GO:0033554~cellular response to stress	0.015108797
c51	GO:0051707~response to other organism	0.016954858
c51	GO:0043523~regulation of neuron apoptosis	0.023830422
c51	GO:0006984~ER-nuclear signaling pathway	0.025173921

c51	GO:0006979~response to oxidative stress	0.030028584
c51	GO:0017144~drug metabolic process	0.033257674
c51	GO:0051234~establishment of localization	0.036301921
c51	GO:0000910~cytokinesis	0.037899324
c51	GO:0006351~transcription, DNA-dependent	0.04074088
c51	GO:0007162~negative regulation of cell adhesion	0.042744575
c51	GO:0032774~RNA biosynthetic process	0.043861695
c51	GO:0006383~transcription from RNA polymerase III promoter	0.045744092
c51	GO:0009301~snRNA transcription	0.053429345
c51	GO:0006810~transport	0.058313926
c51	GO:0007163~establishment or maintenance of cell polarity	0.059023084
c51	GO:0009617~response to bacterium	0.062215842
c51	GO:0006366~transcription from RNA polymerase II promoter	0.063524035
c51	GO:0043524~negative regulation of neuron apoptosis	0.065008767
c51	GO:0051716~cellular response to stimulus	0.069788361
c51	GO:0007097~nuclear migration	0.070599478
c51	GO:0006518~peptide metabolic process	0.071261915
c51	GO:0070647~protein modification by small protein conjugation or removal	0.071397632
c51	GO:0009628~response to abiotic stimulus	0.071984923
c51	GO:0007049~cell cycle	0.072053138
c51	GO:0070727~cellular macromolecule localization	0.075990675
c51	GO:0051179~localization	0.07930574
c51	GO:0008104~protein localization	0.08000247
c51	GO:0033036~macromolecule localization	0.081434377
c51	GO:0006575~cellular amino acid derivative metabolic process	0.081927081
c51	GO:0016070~RNA metabolic process	0.086388658
c51	GO:0046907~intracellular transport	0.089454883
c51	GO:0032446~protein modification by small protein conjugation	0.092312045
c51	GO:0006605~protein targeting	0.096823317
c51	GO:0042391~regulation of membrane potential	0.096877937
c51	GO:0006576~biogenic amine metabolic process	0.099773398
c52	GO:0016070~RNA metabolic process	5.84E-05
c52	GO:0016071~mRNA metabolic process	8.88E-05
c52	GO:0006396~RNA processing	9.86E-05
c52	GO:0006397~mRNA processing	1.11E-04
c52	GO:0000087~M phase of mitotic cell cycle	1.56E-04
c52	GO:0048285~organelle fission	1.98E-04
c52	GO:0044260~cellular macromolecule metabolic process	2.20E-04
c52	GO:0000278~mitotic cell cycle	2.50E-04
c52	GO:0022403~cell cycle phase	4.16E-04
c52	GO:0007067~mitosis	4.30E-04
c52	GO:0000280~nuclear division	4.30E-04
c52	GO:0000279~M phase	4.36E-04
c52	GO:0043170~macromolecule metabolic process	9.03E-04
c52	GO:0022402~cell cycle process	0.001966948

c52	GO:0007049~cell cycle	0.002401607
c52	GO:0051301~cell division	0.002628297
c52	GO:0006139~nucleobase, nucleoside, nucleotide and nucleic acid metabolic process	0.002675672
c52	GO:0010467~gene expression	0.003955122
c52	GO:0008380~RNA splicing	0.004752253
c52	GO:0006807~nitrogen compound metabolic process	0.007600238
c52	GO:0051247~positive regulation of protein metabolic process	0.008528706
c52	GO:0034641~cellular nitrogen compound metabolic process	0.012236383
c52	GO:0032270~positive regulation of cellular protein metabolic process	0.015688462
c52	GO:0044265~cellular macromolecule catabolic process	0.020140947
c52	GO:0006996~organelle organization	0.021523718
c52	GO:0044237~cellular metabolic process	0.023473454
c52	GO:0042517~positive regulation of tyrosine phosphorylation of Stat3 protein	0.023901377
c52	GO:0008593~regulation of Notch signaling pathway	0.023901377
c52	GO:0043392~negative regulation of DNA binding	0.026740405
c52	GO:0006915~apoptosis	0.029073266
c52	GO:0012501~programmed cell death	0.033558749
c52	GO:0044238~primary metabolic process	0.033963949
c52	GO:0043687~post-translational protein modification	0.034743649
c52	GO:0051100~negative regulation of binding	0.042476723
c52	GO:0002702~positive regulation of production of molecular mediator of immune response	0.043040904
c52	GO:0009057~macromolecule catabolic process	0.044339237
c52	GO:0006511~ubiquitin-dependent protein catabolic process	0.04535549
c52	GO:0051603~proteolysis involved in cellular protein catabolic process	0.04841249
c52	GO:0044257~cellular protein catabolic process	0.05028787
c52	GO:0009987~cellular process	0.051900962
c52	GO:0007249~I-kappaB kinase/NF-kappaB cascade	0.0519387
c52	GO:0042516~regulation of tyrosine phosphorylation of Stat3 protein	0.054152781
c52	GO:0000375~RNA splicing, via transesterification reactions	0.055760272
c52	GO:0000377~RNA splicing, via transesterification reactions with bulged adenosine as nucleophile	0.055760272
c52	GO:0000398~nuclear mRNA splicing, via spliceosome	0.055760272
c52	GO:0043632~modification-dependent macromolecule catabolic process	0.056382144
c52	GO:0019941~modification-dependent protein catabolic process	0.056382144
c52	GO:0007017~microtubule-based process	0.058106353
c52	GO:0031401~positive regulation of protein modification process	0.058707211
c52	GO:0042981~regulation of apoptosis	0.058927748
c52	GO:0034504~protein localization in nucleus	0.059137165
c52	GO:0000080~G1 phase of mitotic cell cycle	0.060046371
c52	GO:0043067~regulation of programmed cell death	0.064092988
c52	GO:0006333~chromatin assembly or disassembly	0.065055798
c52	GO:0030163~protein catabolic process	0.066323988
c52	GO:0010941~regulation of cell death	0.066933553
c52	GO:0070647~protein modification by small protein conjugation or removal	0.067519456
c52	GO:0008152~metabolic process	0.070437481
c52	GO:0006029~proteoglycan metabolic process	0.070642623

c52	GO:0034660~ncRNA metabolic process	0.07317002
c52	GO:0051276~chromosome organization	0.077069789
c52	GO:0007051~spindle organization	0.078679594
c52	GO:0043433~negative regulation of transcription factor activity	0.078679594
c52	GO:0000271~polysaccharide biosynthetic process	0.078679594
c52	GO:0006024~glycosaminoglycan biosynthetic process	0.078927767
c52	GO:0051318~G1 phase	0.078927767
c52	GO:0051090~regulation of transcription factor activity	0.080590801
c52	GO:0008219~cell death	0.082277531
c52	GO:0048522~positive regulation of cellular process	0.083328084
c52	GO:0045746~negative regulation of Notch signaling pathway	0.086325697
c52	GO:0050731~positive regulation of peptidyl-tyrosine phosphorylation	0.087095664
c52	GO:0044267~cellular protein metabolic process	0.087705748
c52	GO:0016265~death	0.087844
c52	GO:0043412~biopolymer modification	0.088592564
c52	GO:0008033~tRNA processing	0.089845893
c52	GO:0006023~aminoglycan biosynthetic process	0.092399686
c52	GO:0042531~positive regulation of tyrosine phosphorylation of STAT protein	0.092399686
c54	GO:0032270~positive regulation of cellular protein metabolic process	0.001329532
c54	GO:0051246~regulation of protein metabolic process	0.001649218
c54	GO:0051247~positive regulation of protein metabolic process	0.001691319
c54	GO:0032268~regulation of cellular protein metabolic process	0.001925659
c54	GO:0048518~positive regulation of biological process	0.003459132
c54	GO:0048522~positive regulation of cellular process	0.004453538
c54	GO:0008219~cell death	0.00478451
c54	GO:0016265~death	0.005052881
c54	GO:0030162~regulation of proteolysis	0.006253305
c54	GO:0002694~regulation of leukocyte activation	0.006498252
c54	GO:0051789~response to protein stimulus	0.00701161
c54	GO:0051240~positive regulation of multicellular organismal process	0.007745015
c54	GO:0007588~excretion	0.008029803
c54	GO:0050865~regulation of cell activation	0.008077248
c54	GO:0060349~bone morphogenesis	0.008687414
c54	GO:0042755~eating behavior	0.011409693
c54	GO:0006952~defense response	0.011722217
c54	GO:0008283~cell proliferation	0.012175925
c54	GO:0042110~T cell activation	0.012286674
c54	GO:0045862~positive regulation of proteolysis	0.013405751
c54	GO:0050678~regulation of epithelial cell proliferation	0.013924605
c54	GO:0051094~positive regulation of developmental process	0.014107121
c54	GO:0002358~B cell homeostatic proliferation	0.014118633
c54	GO:0051239~regulation of multicellular organismal process	0.014803326
c54	GO:0009628~response to abiotic stimulus	0.015611872
c54	GO:0007584~response to nutrient	0.017466511
c54	GO:0043281~regulation of caspase activity	0.018502396

c54	GO:0002682~regulation of immune system process	0.019498644
c54	GO:0046903~secretion	0.019779303
c54	GO:0052548~regulation of endopeptidase activity	0.020409426
c54	GO:0050793~regulation of developmental process	0.02094367
c54	GO:0051249~regulation of lymphocyte activation	0.020956336
c54	GO:0006970~response to osmotic stress	0.02146564
c54	GO:0050871~positive regulation of B cell activation	0.022746453
c54	GO:0052547~regulation of peptidase activity	0.023114478
c54	GO:0045595~regulation of cell differentiation	0.023404331
c54	GO:0032501~multicellular organismal process	0.024465799
c54	GO:0006917~induction of apoptosis	0.026140054
c54	GO:0012502~induction of programmed cell death	0.026489973
c54	GO:0042981~regulation of apoptosis	0.026734434
c54	GO:0032502~developmental process	0.02718428
c54	GO:0048856~anatomical structure development	0.027275373
c54	GO:0070472~regulation of uterine smooth muscle contraction	0.028039895
c54	GO:0070474~positive regulation of uterine smooth muscle contraction	0.028039895
c54	GO:0003079~positive regulation of natriuresis	0.028039895
c54	GO:0002118~aggressive behavior	0.028039895
c54	GO:0043067~regulation of programmed cell death	0.02847533
c54	GO:0010941~regulation of cell death	0.029148928
c54	GO:0060627~regulation of vesicle-mediated transport	0.030688518
c54	GO:0051251~positive regulation of lymphocyte activation	0.031509426
c54	GO:0032846~positive regulation of homeostatic process	0.032548323
c54	GO:0043065~positive regulation of apoptosis	0.032968271
c54	GO:0035108~limb morphogenesis	0.03318567
c54	GO:0035107~appendage morphogenesis	0.03318567
c54	GO:0043068~positive regulation of programmed cell death	0.034043382
c54	GO:0051049~regulation of transport	0.034773032
c54	GO:0010942~positive regulation of cell death	0.034773032
c54	GO:0002246~healing during inflammatory response	0.034927367
c54	GO:0042713~sperm ejaculation	0.034927367
c54	GO:0048736~appendage development	0.036675223
c54	GO:0060173~limb development	0.036675223
c54	GO:0030098~lymphocyte differentiation	0.036675223
c54	GO:0070661~leukocyte proliferation	0.038764824
c54	GO:0032943~mononuclear cell proliferation	0.038764824
c54	GO:0002696~positive regulation of leukocyte activation	0.039411484
c54	GO:0010604~positive regulation of macromolecule metabolic process	0.039827336
c54	GO:0007275~multicellular organismal development	0.039859587
c54	GO:0009653~anatomical structure morphogenesis	0.042395362
c54	GO:0050867~positive regulation of cell activation	0.044196263
c54	GO:0008624~induction of apoptosis by extracellular signals	0.045186551
c54	GO:0048705~skeletal system morphogenesis	0.045186551
c54	GO:0031325~positive regulation of cellular metabolic process	0.046717698

c54	GO:0048583~regulation of response to stimulus	0.046990392
c54	GO:0032844~regulation of homeostatic process	0.047200151
c54	GO:0006950~response to stress	0.048305999
c54	GO:0050863~regulation of T cell activation	0.050302481
c54	GO:0050864~regulation of B cell activation	0.050617103
c54	GO:0045580~regulation of T cell differentiation	0.050617103
c54	GO:0031667~response to nutrient levels	0.051324373
c54	GO:0046649~lymphocyte activation	0.052899557
c54	GO:0019538~protein metabolic process	0.052914103
c54	GO:0014070~response to organic cyclic substance	0.054589991
c54	GO:0009607~response to biotic stimulus	0.05515709
c54	GO:0003078~regulation of natriuresis	0.055301198
c54	GO:0044093~positive regulation of molecular function	0.056009027
c54	GO:0008629~induction of apoptosis by intracellular signals	0.056046269
c54	GO:0043279~response to alkaloid	0.056046269
c54	GO:0006919~activation of caspase activity	0.056046269
c54	GO:0009893~positive regulation of metabolic process	0.060962047
c54	GO:0048513~organ development	0.061922528
c54	GO:0044062~regulation of excretion	0.06199739
c54	GO:0006915~apoptosis	0.063533787
c54	GO:0043280~positive regulation of caspase activity	0.065520884
c54	GO:0010952~positive regulation of peptidase activity	0.065520884
c54	GO:0002521~leukocyte differentiation	0.066044695
c54	GO:0048598~embryonic morphogenesis	0.066987876
c54	GO:0012501~programmed cell death	0.068033677
c54	GO:0006508~proteolysis	0.0687517
c54	GO:0030100~regulation of endocytosis	0.069450899
c54	GO:0042391~regulation of membrane potential	0.069680232
c54	GO:0051051~negative regulation of transport	0.070911971
c54	GO:0009991~response to extracellular stimulus	0.071010174
c54	GO:0048878~chemical homeostasis	0.07113813
c54	GO:0045619~regulation of lymphocyte differentiation	0.07345657
c54	GO:0007320~insemination	0.075249124
c54	GO:0043085~positive regulation of catalytic activity	0.075873958
c54	GO:0050776~regulation of immune response	0.076702123
c54	GO:0030217~T cell differentiation	0.077535026
c54	GO:0045597~positive regulation of cell differentiation	0.079632279
c54	GO:0009886~post-embryonic morphogenesis	0.081805316
c54	GO:0008354~germ cell migration	0.081805316
c54	GO:0048024~regulation of nuclear mRNA splicing, via spliceosome	0.081805316
c54	GO:0001508~regulation of action potential	0.083783065
c54	GO:0010647~positive regulation of cell communication	0.084303914
c54	GO:0007631~feeding behavior	0.085899138
c54	GO:0030097~hemopoiesis	0.086684068
c54	GO:0042100~B cell proliferation	0.088315488

c54	GO:0002684~positive regulation of immune system process	0.088753361
c54	GO:0055066~di-, tri-valent inorganic cation homeostasis	0.089796984
c54	GO:0048731~system development	0.091747676
c54	GO:0006937~regulation of muscle contraction	0.092342878
c54	GO:0045321~leukocyte activation	0.092963493
c54	GO:0051098~regulation of binding	0.094706768
c54	GO:0009651~response to salt stress	0.09477996
c54	GO:0033135~regulation of peptidyl-serine phosphorylation	0.09477996
c54	GO:0051216~cartilage development	0.096715057
c54	GO:0009888~tissue development	0.099117777
c55	GO:0046489~phosphoinositide biosynthetic process	9.26E-05
c55	GO:0006506~GPI anchor biosynthetic process	2.83E-04
c55	GO:0006505~GPI anchor metabolic process	3.33E-04
c55	GO:0006497~protein amino acid lipidation	5.64E-04
c55	GO:0030384~phosphoinositide metabolic process	8.28E-04
c55	GO:0042158~lipoprotein biosynthetic process	9.79E-04
c55	GO:0042157~lipoprotein metabolic process	0.001513744
c55	GO:0045017~glycerolipid biosynthetic process	0.001513744
c55	GO:0042246~tissue regeneration	0.001632076
c55	GO:0046474~glycerophospholipid biosynthetic process	0.002501555
c55	GO:0044262~cellular carbohydrate metabolic process	0.004961327
c55	GO:0006650~glycerophospholipid metabolic process	0.005072381
c55	GO:0005975~carbohydrate metabolic process	0.005820849
c55	GO:0040008~regulation of growth	0.006068553
c55	GO:0016254~preassembly of GPI anchor in ER membrane	0.010373601
c55	GO:0046486~glycerolipid metabolic process	0.013657288
c55	GO:0022008~neurogenesis	0.014290563
c55	GO:0016044~membrane organization	0.017684513
c55	GO:0008654~phospholipid biosynthetic process	0.021762208
c55	GO:0045927~positive regulation of growth	0.022162674
c55	GO:0019538~protein metabolic process	0.023698868
c55	GO:0043412~biopolymer modification	0.028928774
c55	GO:0048589~developmental growth	0.031839946
c55	GO:0007051~spindle organization	0.034005225
c55	GO:0006464~protein modification process	0.035930638
c55	GO:0006644~phospholipid metabolic process	0.03623324
c55	GO:0044267~cellular protein metabolic process	0.0372244
c55	GO:0002260~lymphocyte homeostasis	0.040100518
c55	GO:0031099~regeneration	0.040273376
c55	GO:0001558~regulation of cell growth	0.040815969
c55	GO:0005996~monosaccharide metabolic process	0.041944148
c55	GO:0019637~organophosphate metabolic process	0.048421153
c55	GO:0009100~glycoprotein metabolic process	0.051088236
c55	GO:0048699~generation of neurons	0.055655836
c55	GO:0006944~membrane fusion	0.05652345

c55	GO:0008610~lipid biosynthetic process	0.060762969
c55	GO:0070085~glycosylation	0.061760902
c55	GO:0043413~biopolymer glycosylation	0.061760902
c55	GO:0006486~protein amino acid glycosylation	0.061760902
c55	GO:0050954~sensory perception of mechanical stimulus	0.063184576
c55	GO:0048856~anatomical structure development	0.064522941
c55	GO:0009566~fertilization	0.065054477
c55	GO:0009101~glycoprotein biosynthetic process	0.068680798
c55	GO:0048731~system development	0.069040158
c55	GO:0032535~regulation of cellular component size	0.069469496
c55	GO:0016051~carbohydrate biosynthetic process	0.073118685
c55	GO:0001776~leukocyte homeostasis	0.07464193
c55	GO:0032879~regulation of localization	0.075002051
c55	GO:0046364~monosaccharide biosynthetic process	0.079653058
c55	GO:0006923~cleavage of cytoskeletal proteins during apoptosis	0.079817293
c55	GO:0006922~cleavage of lamin	0.079817293
c55	GO:0006066~alcohol metabolic process	0.079920662
c55	GO:0019318~hexose metabolic process	0.081352298
c55	GO:0009308~amine metabolic process	0.083164681
c55	GO:0050867~positive regulation of cell activation	0.083895475
c55	GO:0042551~neuron maturation	0.085578582
c55	GO:0019725~cellular homeostasis	0.087393496
c55	GO:0006082~organic acid metabolic process	0.089257446
c55	GO:0009987~cellular process	0.089487466
c55	GO:0006520~cellular amino acid metabolic process	0.092224151
c55	GO:0032844~regulation of homeostatic process	0.09252466
c55	GO:0045454~cell redox homeostasis	0.093461501
c55	GO:0007033~vacuole organization	0.095524058
c55	GO:0032846~positive regulation of homeostatic process	0.095524058
c55	GO:0046649~lymphocyte activation	0.096313716
c55	GO:0032956~regulation of actin cytoskeleton organization	0.09669928
c56	GO:0006281~DNA repair	9.29E-05
c56	GO:0006974~response to DNA damage stimulus	1.98E-04
c56	GO:0032663~regulation of interleukin-2 production	0.001732451
c56	GO:0006259~DNA metabolic process	0.002423713
c56	GO:0006325~chromatin organization	0.003322759
c56	GO:0007250~activation of NF-kappaB-inducing kinase activity	0.004676943
c56	GO:0051276~chromosome organization	0.005509501
c56	GO:0033554~cellular response to stress	0.005641685
c56	GO:0000075~cell cycle checkpoint	0.006098797
c56	GO:0006835~dicarboxylic acid transport	0.0062305
c56	GO:0016568~chromatin modification	0.006473671
c56	GO:0000077~DNA damage checkpoint	0.006649885
c56	GO:0031570~DNA integrity checkpoint	0.008303133
c56	GO:0009967~positive regulation of signal transduction	0.009527939

c56	GO:0010647~positive regulation of cell communication	0.016538461
c56	GO:0042770~DNA damage response, signal transduction	0.026290714
c56	GO:0051716~cellular response to stimulus	0.031010215
c56	GO:0070536~protein K63-linked deubiquitination	0.031641282
c56	GO:0032703~negative regulation of interleukin-2 production	0.039395289
c56	GO:0002726~positive regulation of T cell cytokine production	0.039395289
c56	GO:0048584~positive regulation of response to stimulus	0.041112916
c56	GO:0043123~positive regulation of I-kappaB kinase/NF-kappaB cascade	0.042835746
c56	GO:0010740~positive regulation of protein kinase cascade	0.04513122
c56	GO:0006950~response to stress	0.045439715
c56	GO:0002724~regulation of T cell cytokine production	0.047087751
c56	GO:0080135~regulation of cellular response to stress	0.049666236
c56	GO:0010627~regulation of protein kinase cascade	0.049732372
c56	GO:0051726~regulation of cell cycle	0.049918121
c56	GO:0043122~regulation of I-kappaB kinase/NF-kappaB cascade	0.054497531
c56	GO:0032743~positive regulation of interleukin-2 production	0.054719153
c56	GO:0001817~regulation of cytokine production	0.057462224
c56	GO:0050863~regulation of T cell activation	0.067514923
c56	GO:0006338~chromatin remodeling	0.073990196
c56	GO:0050789~regulation of biological process	0.074933144
c56	GO:0065007~biological regulation	0.075006256
c56	GO:0002720~positive regulation of cytokine production during immune response	0.077251785
c56	GO:0010604~positive regulation of macromolecule metabolic process	0.081515657
c56	GO:0019222~regulation of metabolic process	0.081636337
c56	GO:0010212~response to ionizing radiation	0.083332425
c56	GO:0048518~positive regulation of biological process	0.083687911
c56	GO:0006302~double-strand break repair	0.088132255
c56	GO:0007249~I-kappaB kinase/NF-kappaB cascade	0.090562653
c56	GO:0030832~regulation of actin filament length	0.090562653
c56	GO:0009966~regulation of signal transduction	0.092825938
c56	GO:0031325~positive regulation of cellular metabolic process	0.093951018
c56	GO:0050794~regulation of cellular process	0.095676072
c56	GO:0048522~positive regulation of cellular process	0.096743686
c56	GO:0031572~G2/M transition DNA damage checkpoint	0.099251948
c57	GO:0007242~intracellular signaling cascade	0.021078539
c57	GO:0009987~cellular process	0.028637459
c57	GO:0042770~DNA damage response, signal transduction	0.029085408
c57	GO:0009100~glycoprotein metabolic process	0.029531684
c57	GO:0030154~cell differentiation	0.0402987
c57	GO:0044093~positive regulation of molecular function	0.044345734
c57	GO:0048662~negative regulation of smooth muscle cell proliferation	0.045638831
c57	GO:0048869~cellular developmental process	0.051206543
c57	GO:0044106~cellular amine metabolic process	0.080839817
c57	GO:0044271~nitrogen compound biosynthetic process	0.093553431
c57	GO:0043085~positive regulation of catalytic activity	0.09392101

c7	GO:0008152~metabolic process	6.03E-04
c7	GO:0032318~regulation of Ras GTPase activity	0.001663003
c7	GO:0044237~cellular metabolic process	0.004100021
c7	GO:0042180~cellular ketone metabolic process	0.004151392
c7	GO:0044238~primary metabolic process	0.004900763
c7	GO:0046394~carboxylic acid biosynthetic process	0.005218681
c7	GO:0016053~organic acid biosynthetic process	0.005218681
c7	GO:0044267~cellular protein metabolic process	0.005338312
c7	GO:0043436~oxoacid metabolic process	0.005458566
c7	GO:0019752~carboxylic acid metabolic process	0.005458566
c7	GO:0006082~organic acid metabolic process	0.00605759
c7	GO:0043087~regulation of GTPase activity	0.006126409
c7	GO:0009119~ribonucleoside metabolic process	0.006810867
c7	GO:0016049~cell growth	0.007263172
c7	GO:0040029~regulation of gene expression, epigenetic	0.007694125
c7	GO:0046578~regulation of Ras protein signal transduction	0.012000901
c7	GO:0045652~regulation of megakaryocyte differentiation	0.01281182
c7	GO:0019538~protein metabolic process	0.014482445
c7	GO:0008610~lipid biosynthetic process	0.018207015
c7	GO:0006464~protein modification process	0.019127395
c7	GO:0006629~lipid metabolic process	0.019365665
c7	GO:0043412~biopolymer modification	0.019699792
c7	GO:0007254~JNK cascade	0.021908538
c7	GO:0046131~pyrimidine ribonucleoside metabolic process	0.023580173
c7	GO:0006004~fucose metabolic process	0.027532432
c7	GO:0009058~biosynthetic process	0.028374672
c7	GO:0031098~stress-activated protein kinase signaling pathway	0.029217658
c7	GO:0044249~cellular biosynthetic process	0.029254823
c7	GO:0009116~nucleoside metabolic process	0.031267151
c7	GO:0006259~DNA metabolic process	0.038295474
c7	GO:0006468~protein amino acid phosphorylation	0.039261708
c7	GO:0040007~growth	0.040846277
c7	GO:0043029~T cell homeostasis	0.041352877
c7	GO:0006513~protein monoubiquitination	0.041790954
c7	GO:0060249~anatomical structure homeostasis	0.043860837
c7	GO:0046483~heterocycle metabolic process	0.044170731
c7	GO:0043525~positive regulation of neuron apoptosis	0.046600662
c7	GO:0042476~odontogenesis	0.049637144
c7	GO:0051056~regulation of small GTPase mediated signal transduction	0.049814052
c7	GO:0019317~fucose catabolic process	0.050976771
c7	GO:0042355~L-fucose catabolic process	0.050976771
c7	GO:0055114~oxidation reduction	0.053875991
c7	GO:0042475~odontogenesis of dentine-containing tooth	0.055809003
c7	GO:0032787~monocarboxylic acid metabolic process	0.057785078
c7	GO:0001101~response to acid	0.058022093

c7	GO:0006213~pyrimidine nucleoside metabolic process	0.06418082
c7	GO:0006493~protein amino acid O-linked glycosylation	0.06418082
c7	GO:0000279~M phase	0.066128329
c7	GO:0009101~glycoprotein biosynthetic process	0.070735167
c7	GO:0043968~histone H2A acetylation	0.071214808
c7	GO:0042354~L-fucose metabolic process	0.071214808
c7	GO:0045654~positive regulation of megakaryocyte differentiation	0.073002982
c7	GO:0046670~positive regulation of retinal cell programmed cell death	0.073002982
c7	GO:0006633~fatty acid biosynthetic process	0.073468157
c7	GO:0032319~regulation of Rho GTPase activity	0.084345494
c7	GO:0002260~lymphocyte homeostasis	0.084345494
c7	GO:0032312~regulation of ARF GTPase activity	0.084345494
c7	GO:0000187~activation of MAPK activity	0.084554537
c7	GO:0001894~tissue homeostasis	0.084605206
c7	GO:0048871~multicellular organismal homeostasis	0.096512654
c58	GO:0044419~interspecies interaction between organisms	0.002712655
c58	GO:0048193~Golgi vesicle transport	0.004171147
c58	GO:0006892~post-Golgi vesicle-mediated transport	0.006152038
c58	GO:0050795~regulation of behavior	0.007513807
c58	GO:0006972~hyperosmotic response	0.011713545
c58	GO:0009651~response to salt stress	0.014494091
c58	GO:0051649~establishment of localization in cell	0.015384851
c58	GO:0016568~chromatin modification	0.017010967
c58	GO:0046907~intracellular transport	0.017084417
c58	GO:0042538~hyperosmotic salinity response	0.019500842
c58	GO:0034504~protein localization in nucleus	0.026360864
c58	GO:0007623~circadian rhythm	0.029514684
c58	GO:0006970~response to osmotic stress	0.031862525
c58	GO:0048583~regulation of response to stimulus	0.032679792
c58	GO:0051641~cellular localization	0.037664868
c58	GO:0000060~protein import into nucleus, translocation	0.038743992
c58	GO:0048520~positive regulation of behavior	0.038743992
c58	GO:0006606~protein import into nucleus	0.044812198
c58	GO:0051170~nuclear import	0.049695504
c58	GO:0033365~protein localization in organelle	0.051604134
c58	GO:0000075~cell cycle checkpoint	0.057630192
c58	GO:0017038~protein import	0.061780606
c58	GO:0051704~multi-organism process	0.062193407
c58	GO:0042423~catecholamine biosynthetic process	0.063086769
c58	GO:0019933~cAMP-mediated signaling	0.069363347
c58	GO:0002376~immune system process	0.072593212
c58	GO:0030330~DNA damage response, signal transduction by p53 class mediator	0.074183363
c58	GO:0034623~cellular macromolecular complex disassembly	0.074183363
c58	GO:0032984~macromolecular complex disassembly	0.074183363
c58	GO:0033077~T cell differentiation in the thymus	0.081202716

c58	GO:0006325~chromatin organization	0.085136599
c58	GO:0043687~post-translational protein modification	0.088951509
c58	GO:0001764~neuron migration	0.095306334
c58	GO:0032103~positive regulation of response to external stimulus	0.095306334
c58	GO:0050921~positive regulation of chemotaxis	0.096054829
c58	GO:0010596~negative regulation of endothelial cell migration	0.096923732
c59	GO:0008543~fibroblast growth factor receptor signaling pathway	0.036362106
c59	GO:0050796~regulation of insulin secretion	0.049825076
c59	GO:0002791~regulation of peptide secretion	0.05709344
c59	GO:0046883~regulation of hormone secretion	0.080943922
c60	GO:0051604~protein maturation	0.012715181
c60	GO:0048511~rhythmic process	0.013931238
c60	GO:0022414~reproductive process	0.020430254
c60	GO:0000003~reproduction	0.020876761
c60	GO:0006916~anti-apoptosis	0.033888458
c60	GO:0051246~regulation of protein metabolic process	0.040212754
c60	GO:0065008~regulation of biological quality	0.049849207
c60	GO:0042552~myelination	0.052519173
c60	GO:0007272~ensheathment of neurons	0.05789246
c60	GO:0008366~axon ensheathment	0.05789246
c60	GO:0044106~cellular amine metabolic process	0.068481465
c60	GO:0042176~regulation of protein catabolic process	0.071199314
c60	GO:0019228~regulation of action potential in neuron	0.073839169
c60	GO:0019724~B cell mediated immunity	0.076471896
c60	GO:0009408~response to heat	0.076471896
c60	GO:0008219~cell death	0.078727465
c60	GO:0010033~response to organic substance	0.079245095
c60	GO:0016265~death	0.08002447
c60	GO:0006950~response to stress	0.081090599
c60	GO:0022602~ovulation cycle process	0.084327485
c60	GO:0008585~female gonad development	0.086931878
c60	GO:0042592~homeostatic process	0.08719517
c60	GO:0006519~cellular amino acid and derivative metabolic process	0.087745664
c60	GO:0043066~negative regulation of apoptosis	0.088599373
c60	GO:0043069~negative regulation of programmed cell death	0.090744972
c60	GO:0042698~ovulation cycle	0.090825277
c60	GO:0060548~negative regulation of cell death	0.091176014
c60	GO:0001508~regulation of action potential	0.092119568
c60	GO:0046660~female sex differentiation	0.093412109
c60	GO:0046545~development of primary female sexual characteristics	0.093412109
c60	GO:0009725~response to hormone stimulus	0.094210956
c60	GO:0002449~lymphocyte mediated immunity	0.094702901
c62	GO:0060052~neurofilament cytoskeleton organization	3.38E-04
c62	GO:0006139~nucleobase, nucleoside, nucleotide and nucleic acid metabolic process	4.22E-04
c62	GO:0034641~cellular nitrogen compound metabolic process	8.12E-04

c62	GO:0010467~gene expression	8.45E-04
c62	GO:0006807~nitrogen compound metabolic process	8.92E-04
c62	GO:0009059~macromolecule biosynthetic process	0.002688989
c62	GO:0043170~macromolecule metabolic process	0.003178416
c62	GO:0044260~cellular macromolecule metabolic process	0.003750786
c62	GO:0006984~ER-nuclear signaling pathway	0.003969133
c62	GO:0031323~regulation of cellular metabolic process	0.004168756
c62	GO:0034645~cellular macromolecule biosynthetic process	0.005325296
c62	GO:0006350~transcription	0.005325403
c62	GO:0060255~regulation of macromolecule metabolic process	0.005789393
c62	GO:0045104~intermediate filament cytoskeleton organization	0.005825697
c62	GO:0019222~regulation of metabolic process	0.006619127
c62	GO:0048729~tissue morphogenesis	0.006813806
c62	GO:0080090~regulation of primary metabolic process	0.007470237
c62	GO:0048519~negative regulation of biological process	0.007595415
c62	GO:0045103~intermediate filament-based process	0.007656423
c62	GO:0003015~heart process	0.008684872
c62	GO:0060047~heart contraction	0.008684872
c62	GO:0045449~regulation of transcription	0.010305685
c62	GO:0048523~negative regulation of cellular process	0.010521615
c62	GO:0045862~positive regulation of proteolysis	0.010974111
c62	GO:0000122~negative regulation of transcription from RNA polymerase II promoter	0.011626859
c62	GO:0019219~regulation of nucleobase, nucleoside, nucleotide and nucleic acid metabolic process	0.012037484
c62	GO:0051252~regulation of RNA metabolic process	0.012081442
c62	GO:0006355~regulation of transcription, DNA-dependent	0.012857255
c62	GO:0055008~cardiac muscle tissue morphogenesis	0.013579605
c62	GO:0060415~muscle tissue morphogenesis	0.013579605
c62	GO:0045732~positive regulation of protein catabolic process	0.013579605
c62	GO:0007417~central nervous system development	0.014100499
c62	GO:0051171~regulation of nitrogen compound metabolic process	0.014597139
c62	GO:0044249~cellular biosynthetic process	0.015817225
c62	GO:0009058~biosynthetic process	0.015848389
c62	GO:0042176~regulation of protein catabolic process	0.016040245
c62	GO:0009889~regulation of biosynthetic process	0.018090198
c62	GO:0010648~negative regulation of cell communication	0.018461394
c62	GO:0065007~biological regulation	0.019503747
c62	GO:0010556~regulation of macromolecule biosynthetic process	0.01964179
c62	GO:0031326~regulation of cellular biosynthetic process	0.02243497
c62	GO:0009968~negative regulation of signal transduction	0.023377852
c62	GO:0050794~regulation of cellular process	0.023554498
c62	GO:0010468~regulation of gene expression	0.02365484
c62	GO:0021516~dorsal spinal cord development	0.023726261
c62	GO:0048534~hemopoietic or lymphoid organ development	0.024678108
c62	GO:0009888~tissue development	0.024950391
c62	GO:0034976~response to endoplasmic reticulum stress	0.025255542

c62	GO:0048731~system development	0.025668948
c62	GO:0006308~DNA catabolic process	0.025754295
c62	GO:0050789~regulation of biological process	0.02614258
c62	GO:0044237~cellular metabolic process	0.027782556
c62	GO:0031331~positive regulation of cellular catabolic process	0.029328457
c62	GO:0006357~regulation of transcription from RNA polymerase II promoter	0.031762146
c62	GO:0030217~T cell differentiation	0.033289662
c62	GO:0002520~immune system development	0.035163203
c62	GO:0045892~negative regulation of transcription, DNA-dependent	0.035878912
c62	GO:0021510~spinal cord development	0.036047372
c62	GO:0007156~homophilic cell adhesion	0.03650207
c62	GO:0051253~negative regulation of RNA metabolic process	0.039970515
c62	GO:0008152~metabolic process	0.045950196
c62	GO:0003007~heart morphogenesis	0.047757092
c62	GO:0007275~multicellular organismal development	0.048224009
c62	GO:0050879~multicellular organismal movement	0.048346043
c62	GO:0050881~musculoskeletal movement	0.048346043
c62	GO:0060048~cardiac muscle contraction	0.048346043
c62	GO:0007507~heart development	0.049270709
c62	GO:0006816~calcium ion transport	0.050624707
c62	GO:0048856~anatomical structure development	0.051185597
c62	GO:0044238~primary metabolic process	0.05273212
c62	GO:0006259~DNA metabolic process	0.053642093
c62	GO:0001655~urogenital system development	0.055495506
c62	GO:0034620~cellular response to unfolded protein	0.057951231
c62	GO:0048169~regulation of long-term neuronal synaptic plasticity	0.057951231
c62	GO:0030968~endoplasmic reticulum unfolded protein response	0.057951231
c62	GO:0032501~multicellular organismal process	0.062080045
c62	GO:0009896~positive regulation of catabolic process	0.06353828
c62	GO:0009887~organ morphogenesis	0.064906017
c62	GO:0031325~positive regulation of cellular metabolic process	0.065237351
c62	GO:0044057~regulation of system process	0.06541702
c62	GO:0009987~cellular process	0.06744191
c62	GO:0052548~regulation of endopeptidase activity	0.067571082
c62	GO:0006921~cell structure disassembly during apoptosis	0.068151744
c62	GO:0014706~striated muscle tissue development	0.072606735
c62	GO:0045110~intermediate filament bundle assembly	0.072743655
c62	GO:0016198~axon choice point recognition	0.072743655
c62	GO:0030705~cytoskeleton-dependent intracellular transport	0.073226423
c62	GO:0030097~hemopoiesis	0.076031706
c62	GO:0043086~negative regulation of catalytic activity	0.07651124
c62	GO:0030162~regulation of proteolysis	0.076593918
c62	GO:0022411~cellular component disassembly	0.076593918
c62	GO:0008285~negative regulation of cell proliferation	0.077165933
c62	GO:0052547~regulation of peptidase activity	0.077548786

c62	GO:0045595~regulation of cell differentiation	0.078943127
c62	GO:0007399~nervous system development	0.079948764
c62	GO:0010604~positive regulation of macromolecule metabolic process	0.080969076
c62	GO:0031324~negative regulation of cellular metabolic process	0.082926783
c62	GO:0002822~regulation of adaptive immune response based on somatic recombination of immune receptors built from immunoglobulin superfamily domains	0.083529026
c62	GO:0010033~response to organic substance	0.08354327
c62	GO:0030262~apoptotic nuclear changes	0.08444512
c62	GO:0006511~ubiquitin-dependent protein catabolic process	0.085209999
c62	GO:0060537~muscle tissue development	0.085444473
c62	GO:0002819~regulation of adaptive immune response	0.087094197
c62	GO:0055001~muscle cell development	0.087094197
c62	GO:0002864~regulation of acute inflammatory response to antigenic stimulus	0.090090287
c62	GO:0060158~activation of phospholipase C activity by dopamine receptor signaling pathway	0.090090287
c62	GO:0002883~regulation of hypersensitivity	0.090090287
c62	GO:0033077~T cell differentiation in the thymus	0.090114361
c62	GO:0048738~cardiac muscle tissue development	0.090722778
c62	GO:0016481~negative regulation of transcription	0.090810872
c62	GO:0050767~regulation of neurogenesis	0.091678025
c62	GO:0006873~cellular ion homeostasis	0.093163383
c62	GO:0010605~negative regulation of macromolecule metabolic process	0.094499448
c62	GO:0009892~negative regulation of metabolic process	0.094755282
c62	GO:0009893~positive regulation of metabolic process	0.095304508
c62	GO:0048513~organ development	0.095427142
c62	GO:0044092~negative regulation of molecular function	0.097532708
c63	GO:0032268~regulation of cellular protein metabolic process	5.10E-04
c63	GO:0051246~regulation of protein metabolic process	0.001026554
c63	GO:0048519~negative regulation of biological process	0.001133867
c63	GO:0048523~negative regulation of cellular process	0.001189192
c63	GO:0016070~RNA metabolic process	0.003446841
c63	GO:0032270~positive regulation of cellular protein metabolic process	0.003879005
c63	GO:0051247~positive regulation of protein metabolic process	0.00517963
c63	GO:0006396~RNA processing	0.005884403
c63	GO:0050732~negative regulation of peptidyl-tyrosine phosphorylation	0.008241761
c63	GO:0010605~negative regulation of macromolecule metabolic process	0.01189535
c63	GO:0031333~negative regulation of protein complex assembly	0.016043372
c63	GO:0010604~positive regulation of macromolecule metabolic process	0.016113535
c63	GO:0065003~macromolecular complex assembly	0.017681463
c63	GO:0031325~positive regulation of cellular metabolic process	0.02112502
c63	GO:0009892~negative regulation of metabolic process	0.021557433
c63	GO:0032269~negative regulation of cellular protein metabolic process	0.02380854
c63	GO:0051129~negative regulation of cellular component organization	0.024767478
c63	GO:0051248~negative regulation of protein metabolic process	0.028536663
c63	GO:0043200~response to amino acid stimulus	0.028671593
c63	GO:0070271~protein complex biogenesis	0.028679764
c63	GO:0006461~protein complex assembly	0.028679764

c63	GO:0043933~macromolecular complex subunit organization	0.031377418
c63	GO:0009893~positive regulation of metabolic process	0.032984435
c63	GO:0002250~adaptive immune response	0.033054964
c63	GO:0002460~adaptive immune response based on somatic recombination of immune receptors built from immunoglobulin superfamily domains	0.033054964
c63	GO:0031324~negative regulation of cellular metabolic process	0.035281395
c63	GO:0050688~regulation of defense response to virus	0.035319695
c63	GO:0044255~cellular lipid metabolic process	0.038529124
c63	GO:0031400~negative regulation of protein modification process	0.039779064
c63	GO:0046470~phosphatidylcholine metabolic process	0.042498333
c63	GO:0031399~regulation of protein modification process	0.043884633
c63	GO:0002443~leukocyte mediated immunity	0.046588756
c63	GO:0010608~posttranscriptional regulation of gene expression	0.0495512
c63	GO:0016064~immunoglobulin mediated immune response	0.053009226
c63	GO:0001101~response to acid	0.054175665
c63	GO:0019724~B cell mediated immunity	0.057925354
c63	GO:0019538~protein metabolic process	0.060597556
c63	GO:0001561~fatty acid alpha-oxidation	0.061445212
c63	GO:0055086~nucleobase, nucleoside and nucleotide metabolic process	0.062372668
c63	GO:0044267~cellular protein metabolic process	0.065210291
c63	GO:0006417~regulation of translation	0.065215591
c63	GO:0051236~establishment of RNA localization	0.066720837
c63	GO:0050658~RNA transport	0.066720837
c63	GO:0050657~nucleic acid transport	0.066720837
c63	GO:0006403~RNA localization	0.072882384
c63	GO:0002697~regulation of immune effector process	0.074998591
c63	GO:0031401~positive regulation of protein modification process	0.075188088
c63	GO:0006958~complement activation, classical pathway	0.075761075
c63	GO:0002831~regulation of response to biotic stimulus	0.075761075
c63	GO:0010243~response to organic nitrogen	0.076733188
c63	GO:0000184~nuclear-transcribed mRNA catabolic process, nonsense-mediated decay	0.080358635
c63	GO:0001775~cell activation	0.083910409
c63	GO:0008283~cell proliferation	0.084174269
c63	GO:0002455~humoral immune response mediated by circulating immunoglobulin	0.085040449
c63	GO:0043900~regulation of multi-organism process	0.085040449
c63	GO:0001933~negative regulation of protein amino acid phosphorylation	0.089802759
c63	GO:0030168~platelet activation	0.089802759
c63	GO:0006629~lipid metabolic process	0.090049515
c63	GO:0009117~nucleotide metabolic process	0.09092336
c63	GO:0006753~nucleoside phosphate metabolic process	0.09092336
c63	GO:0046474~glycerophospholipid biosynthetic process	0.091599455
c63	GO:0050730~regulation of peptidyl-tyrosine phosphorylation	0.091599455
c63	GO:0030258~lipid modification	0.09470659
c63	GO:0019637~organophosphate metabolic process	0.096159033
c63	GO:0002449~lymphocyte mediated immunity	0.097856588
c63	GO:0042439~ethanolamine and derivative metabolic process	0.099554309

c64	GO:0009987~cellular process	4.75E-06
c64	GO:0006139~nucleobase, nucleoside, nucleotide and nucleic acid metabolic process	6.09E-05
c64	GO:0034641~cellular nitrogen compound metabolic process	1.36E-04
c64	GO:0032774~RNA biosynthetic process	1.59E-04
c64	GO:0044265~cellular macromolecule catabolic process	1.68E-04
c64	GO:0006807~nitrogen compound metabolic process	2.61E-04
c64	GO:0016070~RNA metabolic process	2.67E-04
c64	GO:0044260~cellular macromolecule metabolic process	3.00E-04
c64	GO:0006351~transcription, DNA-dependent	3.47E-04
c64	GO:0044248~cellular catabolic process	6.03E-04
c64	GO:0006259~DNA metabolic process	6.16E-04
c64	GO:0044085~cellular component biogenesis	6.47E-04
c64	GO:0009057~macromolecule catabolic process	7.79E-04
c64	GO:0044237~cellular metabolic process	9.15E-04
c64	GO:0006366~transcription from RNA polymerase II promoter	0.001112536
c64	GO:0006403~RNA localization	0.001125982
c64	GO:0051603~proteolysis involved in cellular protein catabolic process	0.001162945
c64	GO:0044238~primary metabolic process	0.001250473
c64	GO:0044257~cellular protein catabolic process	0.00128075
c64	GO:0043170~macromolecule metabolic process	0.001589376
c64	GO:0016043~cellular component organization	0.001953505
c64	GO:0030163~protein catabolic process	0.002136134
c64	GO:0019941~modification-dependent protein catabolic process	0.002184178
c64	GO:0043632~modification-dependent macromolecule catabolic process	0.002184178
c64	GO:0009056~catabolic process	0.002490995
c64	GO:0019953~sexual reproduction	0.002506548
c64	GO:0022607~cellular component assembly	0.002732322
c64	GO:0015931~nucleobase, nucleoside, nucleotide and nucleic acid transport	0.002823318
c64	GO:0000278~mitotic cell cycle	0.003030124
c64	GO:0051236~establishment of RNA localization	0.003291174
c64	GO:0050658~RNA transport	0.003291174
c64	GO:0050657~nucleic acid transport	0.003291174
c64	GO:0022402~cell cycle process	0.003306754
c64	GO:0006913~nucleocytoplasmic transport	0.00362603
c64	GO:0051169~nuclear transport	0.004019147
c64	GO:0006352~transcription initiation	0.00433675
c64	GO:0006461~protein complex assembly	0.004663761
c64	GO:0070271~protein complex biogenesis	0.004663761
c64	GO:0006367~transcription initiation from RNA polymerase II promoter	0.005199512
c64	GO:0045591~positive regulation of regulatory T cell differentiation	0.005603682
c64	GO:0022403~cell cycle phase	0.005644537
c64	GO:0051028~mRNA transport	0.005769529
c64	GO:0007276~gamete generation	0.006571438
c64	GO:0006974~response to DNA damage stimulus	0.006896272
c64	GO:0006357~regulation of transcription from RNA polymerase II promoter	0.007408524

c64	GO:0016579~protein deubiquitination	0.008142988
c64	GO:0006405~RNA export from nucleus	0.00863606
c64	GO:0043065~positive regulation of apoptosis	0.008756222
c64	GO:0043933~macromolecular complex subunit organization	0.008857842
c64	GO:0045589~regulation of regulatory T cell differentiation	0.009146815
c64	GO:0007067~mitosis	0.0094276
c64	GO:0000280~nuclear division	0.0094276
c64	GO:0043068~positive regulation of programmed cell death	0.009495112
c64	GO:0010942~positive regulation of cell death	0.009943422
c64	GO:0051168~nuclear export	0.0108366
c64	GO:0043543~protein amino acid acylation	0.0108366
c64	GO:0000087~M phase of mitotic cell cycle	0.010894848
c64	GO:0044267~cellular protein metabolic process	0.011594861
c64	GO:0070646~protein modification by small protein removal	0.012021365
c64	GO:0048285~organelle fission	0.013031068
c64	GO:0008152~metabolic process	0.013876672
c64	GO:0000279~M phase	0.015544703
c64	GO:0006281~DNA repair	0.016932677
c64	GO:0007283~spermatogenesis	0.017087547
c64	GO:0048232~male gamete generation	0.017087547
c64	GO:0065003~macromolecular complex assembly	0.017120473
c64	GO:0002718~regulation of cytokine production during immune response	0.017429493
c64	GO:0034645~cellular macromolecule biosynthetic process	0.017680249
c64	GO:0007049~cell cycle	0.018339007
c64	GO:0006406~mRNA export from nucleus	0.018788451
c64	GO:0010467~gene expression	0.019078598
c64	GO:0006508~proteolysis	0.020065377
c64	GO:0009059~macromolecule biosynthetic process	0.02150181
c64	GO:0051301~cell division	0.023365835
c64	GO:0006917~induction of apoptosis	0.023650627
c64	GO:0012502~induction of programmed cell death	0.024328124
c64	GO:0032502~developmental process	0.024628828
c64	GO:0032844~regulation of homeostatic process	0.026433923
c64	GO:0006996~organelle organization	0.028104773
c64	GO:0019538~protein metabolic process	0.030593733
c64	GO:0051276~chromosome organization	0.031462002
c64	GO:0033554~cellular response to stress	0.032135857
c64	GO:0048609~reproductive process in a multicellular organism	0.03254659
c64	GO:0032504~multicellular organism reproduction	0.03254659
c64	GO:0010556~regulation of macromolecule biosynthetic process	0.036005454
c64	GO:0070727~cellular macromolecule localization	0.036264102
c64	GO:0006260~DNA replication	0.036453321
c64	GO:0002720~positive regulation of cytokine production during immune response	0.037117926
c64	GO:0006605~protein targeting	0.037963511
c64	GO:0002700~regulation of production of molecular mediator of immune response	0.038320371

c64	GO:0033365~protein localization in organelle	0.039692925
c64	GO:0045449~regulation of transcription	0.040217752
c64	GO:0055086~nucleobase, nucleoside and nucleotide metabolic process	0.040595727
c64	GO:0007059~chromosome segregation	0.040930666
c64	GO:0006511~ubiquitin-dependent protein catabolic process	0.041045354
c64	GO:0007050~cell cycle arrest	0.042389573
c64	GO:0009117~nucleotide metabolic process	0.042439008
c64	GO:0006753~nucleoside phosphate metabolic process	0.042439008
c64	GO:0051259~protein oligomerization	0.046656916
c64	GO:0009892~negative regulation of metabolic process	0.047168474
c64	GO:0060255~regulation of macromolecule metabolic process	0.047492555
c64	GO:0007275~multicellular organismal development	0.047997235
c64	GO:0009314~response to radiation	0.049820635
c64	GO:0007292~female gamete generation	0.052139607
c64	GO:0051641~cellular localization	0.052905584
c64	GO:0022414~reproductive process	0.054802201
c64	GO:0000387~spliceosomal snRNP biogenesis	0.055707552
c64	GO:0006350~transcription	0.055998989
c64	GO:0022613~ribonucleoprotein complex biogenesis	0.056255065
c64	GO:0031326~regulation of cellular biosynthetic process	0.056655946
c64	GO:0010468~regulation of gene expression	0.05673761
c64	GO:0019222~regulation of metabolic process	0.056977997
c64	GO:0034613~cellular protein localization	0.058166265
c64	GO:0000003~reproduction	0.058307929
c64	GO:0044249~cellular biosynthetic process	0.0592021
c64	GO:0044092~negative regulation of molecular function	0.060488148
c64	GO:0006915~apoptosis	0.061499658
c64	GO:0016573~histone acetylation	0.062265946
c64	GO:0006368~RNA elongation from RNA polymerase II promoter	0.062265946
c64	GO:0070647~protein modification by small protein conjugation or removal	0.063800918
c64	GO:0031099~regeneration	0.064259474
c64	GO:0009889~regulation of biosynthetic process	0.065317725
c64	GO:0006364~rRNA processing	0.06801349
c64	GO:0031323~regulation of cellular metabolic process	0.069040217
c64	GO:0012501~programmed cell death	0.069136408
c64	GO:0043392~negative regulation of DNA binding	0.074461065
c64	GO:0006354~RNA elongation	0.074461065
c64	GO:0009058~biosynthetic process	0.077103441
c64	GO:0002702~positive regulation of production of molecular mediator of immune response	0.078212486
c64	GO:0051171~regulation of nitrogen compound metabolic process	0.078627141
c64	GO:0006473~protein amino acid acetylation	0.078775281
c64	GO:0016072~rRNA metabolic process	0.079934644
c64	GO:0046907~intracellular transport	0.087381081
c64	GO:0006919~activation of caspase activity	0.087767511
c64	GO:0016568~chromatin modification	0.089725937

c64	GO:0032831~positive regulation of CD4-positive, CD25-positive, alpha-beta regulatory T cell differentiation	0.090832131
c64	GO:0032829~regulation of CD4-positive, CD25-positive, alpha-beta regulatory T cell differentiation	0.090832131
c64	GO:0006396~RNA processing	0.091362465
c64	GO:0080090~regulation of primary metabolic process	0.091737767
c64	GO:0008219~cell death	0.093193486
c64	GO:0002520~immune system development	0.093462923
c64	GO:0042981~regulation of apoptosis	0.094982961
c64	GO:0051726~regulation of cell cycle	0.095932142
c64	GO:0031324~negative regulation of cellular metabolic process	0.096475903
c64	GO:0048771~tissue remodeling	0.097231982
c65	GO:0006469~negative regulation of protein kinase activity	0.004808834
c65	GO:0033673~negative regulation of kinase activity	0.00513729
c65	GO:0051348~negative regulation of transferase activity	0.005824461
c65	GO:0007154~cell communication	0.013189176
c65	GO:0007275~multicellular organismal development	0.040410012
c65	GO:0043086~negative regulation of catalytic activity	0.042986242
c65	GO:0044419~interspecies interaction between organisms	0.044683748
c65	GO:0048731~system development	0.048249146
c65	GO:0044092~negative regulation of molecular function	0.060089513
c65	GO:0001889~liver development	0.06198069
c65	GO:0045859~regulation of protein kinase activity	0.063627638
c65	GO:0032502~developmental process	0.063651495
c65	GO:0019226~transmission of nerve impulse	0.065259575
c65	GO:0043549~regulation of kinase activity	0.067568627
c65	GO:0048856~anatomical structure development	0.067822024
c65	GO:0051338~regulation of transferase activity	0.072609739
c65	GO:0022607~cellular component assembly	0.087051361
c66	GO:0007178~transmembrane receptor protein serine/threonine kinase signaling pathway	0.047865066
c67	GO:0034645~cellular macromolecule biosynthetic process	0.002776592
c67	GO:0009059~macromolecule biosynthetic process	0.002994041
c67	GO:0044260~cellular macromolecule metabolic process	0.006418848
c67	GO:0044249~cellular biosynthetic process	0.008860943
c67	GO:0043170~macromolecule metabolic process	0.009700499
c67	GO:0009058~biosynthetic process	0.011894292
c67	GO:0008152~metabolic process	0.013688125
c67	GO:0010467~gene expression	0.013710121
c67	GO:0044237~cellular metabolic process	0.016640847
c67	GO:0006350~transcription	0.028231798
c67	GO:0030099~myeloid cell differentiation	0.028479632
c67	GO:0044238~primary metabolic process	0.030717189
c67	GO:0021522~spinal cord motor neuron differentiation	0.036232922
c67	GO:0006139~nucleobase, nucleoside, nucleotide and nucleic acid metabolic process	0.041174488
c67	GO:0021517~ventral spinal cord development	0.041692381
c67	GO:0045124~regulation of bone resorption	0.041692381
c67	GO:0046850~regulation of bone remodeling	0.041692381

c67	GO:0010556~regulation of macromolecule biosynthetic process	0.043760211
c67	GO:0010468~regulation of gene expression	0.04671274
c67	GO:0002763~positive regulation of myeloid leukocyte differentiation	0.049825076
c67	GO:0045449~regulation of transcription	0.051834399
c67	GO:0034103~regulation of tissue remodeling	0.05252099
c67	GO:0031326~regulation of cellular biosynthetic process	0.058187248
c67	GO:0045670~regulation of osteoclast differentiation	0.060564062
c67	GO:0009889~regulation of biosynthetic process	0.060815066
c67	GO:0005975~carbohydrate metabolic process	0.061979242
c67	GO:0021515~cell differentiation in spinal cord	0.068540551
c67	GO:0034641~cellular nitrogen compound metabolic process	0.073024859
c67	GO:0007566~embryo implantation	0.08168819
c67	GO:0019219~regulation of nucleobase, nucleoside, nucleotide and nucleic acid metabolic process	0.085485788
c67	GO:0009987~cellular process	0.088344996
c67	GO:0045639~positive regulation of myeloid cell differentiation	0.089489735
c67	GO:0051171~regulation of nitrogen compound metabolic process	0.090207261
c67	GO:0006807~nitrogen compound metabolic process	0.090275112
c67	GO:0002573~myeloid leukocyte differentiation	0.092075861
c8	GO:0006814~sodium ion transport	0.0134515
c8	GO:0030001~metal ion transport	0.032003485
c8	GO:0015672~monovalent inorganic cation transport	0.033116953
c8	GO:0042953~lipoprotein transport	0.037614391
c8	GO:0051179~localization	0.050651895
c8	GO:0034660~ncRNA metabolic process	0.057749864
c8	GO:0006812~cation transport	0.059514801
c8	GO:0006810~transport	0.06480853
c8	GO:0055085~transmembrane transport	0.065635243
c8	GO:0051234~establishment of localization	0.069771661
c8	GO:0006811~ion transport	0.071816219
c8	GO:0045184~establishment of protein localization	0.072173236
c8	GO:0007049~cell cycle	0.074701589
c8	GO:0000086~G2/M transition of mitotic cell cycle	0.077387175
c8	GO:0040011~locomotion	0.082268623
c8	GO:0007031~peroxisome organization	0.084443211
c8	GO:0006281~DNA repair	0.094644808
c68	GO:0005996~monosaccharide metabolic process	0.062513
c68	GO:0005975~carbohydrate metabolic process	0.072233481
c69	GO:0032869~cellular response to insulin stimulus	0.047156043
c69	GO:0032868~response to insulin stimulus	0.06864659
c69	GO:0032870~cellular response to hormone stimulus	0.090350877
c70	GO:0008202~steroid metabolic process	0.014571976
c70	GO:0008206~bile acid metabolic process	0.047388908
c70	GO:0016042~lipid catabolic process	0.071734057
c70	GO:0009987~cellular process	0.076776889
c72	GO:0016055~Wnt receptor signaling pathway	0.055226165

c72	GO:0019538~protein metabolic process	0.097863964
c73	GO:0007242~intracellular signaling cascade	0.026771396
c73	GO:0007275~multicellular organismal development	0.029654231
c73	GO:0006350~transcription	0.068396513
c73	GO:0032502~developmental process	0.073950971
c73	GO:0048731~system development	0.083843327
c74	GO:0044085~cellular component biogenesis	2.34E-04
c74	GO:0051301~cell division	0.001087599
c74	GO:0022607~cellular component assembly	0.003021885
c74	GO:0040015~negative regulation of multicellular organism growth	0.004028619
c74	GO:0043933~macromolecular complex subunit organization	0.0067258
c74	GO:0022613~ribonucleoprotein complex biogenesis	0.00696414
c74	GO:0030168~platelet activation	0.007022733
c74	GO:0007049~cell cycle	0.007331759
c74	GO:0065003~macromolecular complex assembly	0.008116003
c74	GO:0016070~RNA metabolic process	0.011618783
c74	GO:0000278~mitotic cell cycle	0.016483673
c74	GO:0042254~ribosome biogenesis	0.017529556
c74	GO:0000280~nuclear division	0.019381467
c74	GO:0007067~mitosis	0.019381467
c74	GO:0007626~locomotory behavior	0.020374412
c74	GO:0000279~M phase	0.020881918
c74	GO:0000087~M phase of mitotic cell cycle	0.021234447
c74	GO:0006461~protein complex assembly	0.022601392
c74	GO:0070271~protein complex biogenesis	0.022601392
c74	GO:0048285~organelle fission	0.023445353
c74	GO:0007423~sensory organ development	0.023445353
c74	GO:0002673~regulation of acute inflammatory response	0.027208639
c74	GO:0022403~cell cycle phase	0.032556375
c74	GO:0016043~cellular component organization	0.044197543
c74	GO:0022402~cell cycle process	0.047076045
c74	GO:0045926~negative regulation of growth	0.04710124
c74	GO:0042330~taxis	0.047978664
c74	GO:0006935~chemotaxis	0.047978664
c74	GO:0002674~negative regulation of acute inflammatory response	0.048406623
c74	GO:0033119~negative regulation of RNA splicing	0.060139705
c74	GO:0007610~behavior	0.065146514
c74	GO:0006351~transcription, DNA-dependent	0.070120366
c74	GO:0032774~RNA biosynthetic process	0.07398651
c74	GO:0034621~cellular macromolecular complex subunit organization	0.074782768
c74	GO:0001654~eye development	0.080472213
c74	GO:0046777~protein amino acid autophosphorylation	0.087587809
c74	GO:0006974~response to DNA damage stimulus	0.09076348
c74	GO:0006950~response to stress	0.092023806
c74	GO:0044267~cellular protein metabolic process	0.095134429

c74	GO:0034622~cellular macromolecular complex assembly	0.098814223
c75	GO:0000122~negative regulation of transcription from RNA polymerase II promoter	0.03567721
c75	GO:0045892~negative regulation of transcription, DNA-dependent	0.06030522
c75	GO:0051253~negative regulation of RNA metabolic process	0.06211403
c75	GO:0006968~cellular defense response	0.066979537
c75	GO:0016481~negative regulation of transcription	0.09379594
c76	GO:0000280~nuclear division	4.67E-04
c76	GO:0007067~mitosis	4.67E-04
c76	GO:0000087~M phase of mitotic cell cycle	5.76E-04
c76	GO:0048285~organelle fission	7.34E-04
c76	GO:0022403~cell cycle phase	0.00105635
c76	GO:0000279~M phase	0.001323883
c76	GO:0007517~muscle organ development	0.002269814
c76	GO:0000278~mitotic cell cycle	0.002476339
c76	GO:0051301~cell division	0.004595711
c76	GO:0000070~mitotic sister chromatid segregation	0.004611059
c76	GO:0000819~sister chromatid segregation	0.005199586
c76	GO:0009264~deoxyribonucleotide catabolic process	0.008159909
c76	GO:0007386~compartment specification	0.008867348
c76	GO:0032318~regulation of Ras GTPase activity	0.014789524
c76	GO:0060537~muscle tissue development	0.015011224
c76	GO:0001709~cell fate determination	0.017822212
c76	GO:0055002~striated muscle cell development	0.021343483
c76	GO:0006285~base-excision repair, AP site formation	0.023354466
c76	GO:0022402~cell cycle process	0.026833333
c76	GO:0055001~muscle cell development	0.028421648
c76	GO:0014706~striated muscle tissue development	0.030428767
c76	GO:0006816~calcium ion transport	0.031295591
c76	GO:0043269~regulation of ion transport	0.032829193
c76	GO:0043087~regulation of GTPase activity	0.036012698
c76	GO:0010959~regulation of metal ion transport	0.038338792
c76	GO:0016049~cell growth	0.039148801
c76	GO:0050819~negative regulation of coagulation	0.044460699
c76	GO:0007188~G-protein signaling, coupled to cAMP nucleotide second messenger	0.046733717
c76	GO:0006284~base-excision repair	0.048914801
c76	GO:0009262~deoxyribonucleotide metabolic process	0.048914801
c76	GO:0030534~adult behavior	0.048996806
c76	GO:0006941~striated muscle contraction	0.052207899
c76	GO:0018108~peptidyl-tyrosine phosphorylation	0.052207899
c76	GO:0006793~phosphorus metabolic process	0.052784478
c76	GO:0006796~phosphate metabolic process	0.052784478
c76	GO:0051146~striated muscle cell differentiation	0.053722417
c76	GO:0032313~regulation of Rab GTPase activity	0.055738081
c76	GO:0032483~regulation of Rab protein signal transduction	0.055738081
c76	GO:0007076~mitotic chromosome condensation	0.058813381

c76	GO:0018212~peptidyl-tyrosine modification	0.059393705
c76	GO:0046578~regulation of Ras protein signal transduction	0.060703857
c76	GO:0051924~regulation of calcium ion transport	0.060837713
c76	GO:0006357~regulation of transcription from RNA polymerase II promoter	0.06220816
c76	GO:0048468~cell development	0.063649511
c76	GO:0007187~G-protein signaling, coupled to cyclic nucleotide second messenger	0.064246773
c76	GO:0006259~DNA metabolic process	0.065269633
c76	GO:0002028~regulation of sodium ion transport	0.067255626
c76	GO:0051954~positive regulation of amine transport	0.067255626
c76	GO:0002253~activation of immune response	0.069506128
c76	GO:0006397~mRNA processing	0.071914945
c76	GO:0019933~cAMP-mediated signaling	0.072371491
c76	GO:0006509~membrane protein ectodomain proteolysis	0.076068762
c76	GO:0007268~synaptic transmission	0.077348895
c76	GO:0006304~DNA modification	0.079877408
c76	GO:0051604~protein maturation	0.082281381
c76	GO:0046700~heterocycle catabolic process	0.08812603
c76	GO:0035188~hatching	0.089435151
c76	GO:0001835~blastocyst hatching	0.089435151
c76	GO:0008284~positive regulation of cell proliferation	0.0896124
c76	GO:0051056~regulation of small GTPase mediated signal transduction	0.09018244
c76	GO:0007281~germ cell development	0.090959812
c76	GO:0048747~muscle fiber development	0.091695047
c76	GO:0015674~di-, tri-valent inorganic cation transport	0.093634604
c76	GO:0050684~regulation of mRNA processing	0.094686564
c76	GO:0048738~cardiac muscle tissue development	0.097738517
c76	GO:0000377~RNA splicing, via transesterification reactions with bulged adenosine as nucleophile	0.099890384
c76	GO:0000375~RNA splicing, via transesterification reactions	0.099890384
c76	GO:0000398~nuclear mRNA splicing, via spliceosome	0.099890384
c77	GO:0051258~protein polymerization	2.11E-04
c77	GO:0032504~multicellular organism reproduction	3.75E-04
c77	GO:0048609~reproductive process in a multicellular organism	3.75E-04
c77	GO:0007276~gamete generation	5.01E-04
c77	GO:0007017~microtubule-based process	5.62E-04
c77	GO:0019953~sexual reproduction	6.35E-04
c77	GO:0007283~spermatogenesis	6.99E-04
c77	GO:0048232~male gamete generation	6.99E-04
c77	GO:0022414~reproductive process	8.73E-04
c77	GO:0000278~mitotic cell cycle	9.36E-04
c77	GO:0000003~reproduction	9.43E-04
c77	GO:0051726~regulation of cell cycle	0.001254658
c77	GO:0022402~cell cycle process	0.001693286
c77	GO:0051052~regulation of DNA metabolic process	0.001765058
c77	GO:0000075~cell cycle checkpoint	0.003539142
c77	GO:0006259~DNA metabolic process	0.004518996

c77	GO:0031396~regulation of protein ubiquitination	0.005288994
c77	GO:0022403~cell cycle phase	0.006907596
c77	GO:0051439~regulation of ubiquitin-protein ligase activity during mitotic cell cycle	0.008212301
c77	GO:0006730~one-carbon metabolic process	0.008473113
c77	GO:0007018~microtubule-based movement	0.008787262
c77	GO:0031397~negative regulation of protein ubiquitination	0.009478363
c77	GO:0043623~cellular protein complex assembly	0.009779891
c77	GO:0009225~nucleotide-sugar metabolic process	0.010371681
c77	GO:0031400~negative regulation of protein modification process	0.010842843
c77	GO:0044237~cellular metabolic process	0.011353053
c77	GO:0051438~regulation of ubiquitin-protein ligase activity	0.011354924
c77	GO:0000279~M phase	0.011797411
c77	GO:0007067~mitosis	0.011945339
c77	GO:0000280~nuclear division	0.011945339
c77	GO:0051340~regulation of ligase activity	0.012908854
c77	GO:0000087~M phase of mitotic cell cycle	0.013086135
c77	GO:0016569~covalent chromatin modification	0.013630293
c77	GO:0048285~organelle fission	0.014620195
c77	GO:0009987~cellular process	0.014621669
c77	GO:0010605~negative regulation of macromolecule metabolic process	0.017683681
c77	GO:0031399~regulation of protein modification process	0.018012023
c77	GO:0044238~primary metabolic process	0.01857103
c77	GO:0008152~metabolic process	0.018603647
c77	GO:0031401~positive regulation of protein modification process	0.018712319
c77	GO:0034621~cellular macromolecular complex subunit organization	0.019110888
c77	GO:0043170~macromolecule metabolic process	0.020705552
c77	GO:0006044~N-acetylglucosamine metabolic process	0.020727071
c77	GO:0006041~glucosamine metabolic process	0.020727071
c77	GO:0051774~negative regulation of nitric-oxide synthase 2 biosynthetic process	0.022261447
c77	GO:0006048~UDP-N-acetylglucosamine biosynthetic process	0.022261447
c77	GO:0006042~glucosamine biosynthetic process	0.022261447
c77	GO:0006045~N-acetylglucosamine biosynthetic process	0.022261447
c77	GO:0044260~cellular macromolecule metabolic process	0.023040657
c77	GO:0006974~response to DNA damage stimulus	0.024537788
c77	GO:0051054~positive regulation of DNA metabolic process	0.024752492
c77	GO:0034622~cellular macromolecular complex assembly	0.026746537
c77	GO:0007049~cell cycle	0.027468842
c77	GO:0009892~negative regulation of metabolic process	0.028577407
c77	GO:0010604~positive regulation of macromolecule metabolic process	0.030598047
c77	GO:0006298~mismatch repair	0.031559681
c77	GO:0006040~amino sugar metabolic process	0.031559681
c77	GO:0043412~biopolymer modification	0.032864033
c77	GO:0051772~regulation of nitric-oxide synthase 2 biosynthetic process	0.033206798
c77	GO:0046349~amino sugar biosynthetic process	0.033206798
c77	GO:0016568~chromatin modification	0.034587269

c77	GO:0051436~negative regulation of ubiquitin-protein ligase activity during mitotic cell cycle	0.036287751
c77	GO:0031145~anaphase-promoting complex-dependent proteasomal ubiquitin-dependent protein catabolic process	0.036287751
c77	GO:0031325~positive regulation of cellular metabolic process	0.037638903
c77	GO:0051444~negative regulation of ubiquitin-protein ligase activity	0.039166471
c77	GO:0051352~negative regulation of ligase activity	0.039166471
c77	GO:0032268~regulation of cellular protein metabolic process	0.040541337
c77	GO:0051437~positive regulation of ubiquitin-protein ligase activity during mitotic cell cycle	0.040648187
c77	GO:0006281~DNA repair	0.040710599
c77	GO:0006396~RNA processing	0.043557496
c77	GO:0051443~positive regulation of ubiquitin-protein ligase activity	0.043695642
c77	GO:0018344~protein geranylgeranylation	0.044030388
c77	GO:0018348~protein amino acid geranylgeranylation	0.044030388
c77	GO:0051276~chromosome organization	0.046122598
c77	GO:0048519~negative regulation of biological process	0.046620497
c77	GO:0032270~positive regulation of cellular protein metabolic process	0.047167472
c77	GO:0016570~histone modification	0.048153994
c77	GO:0051301~cell division	0.048220102
c77	GO:0051351~positive regulation of ligase activity	0.048474594
c77	GO:0032269~negative regulation of cellular protein metabolic process	0.051803883
c77	GO:0009893~positive regulation of metabolic process	0.053011001
c77	GO:0033554~cellular response to stress	0.053244277
c77	GO:0032259~methylation	0.053498822
c77	GO:0006464~protein modification process	0.055397205
c77	GO:0051247~positive regulation of protein metabolic process	0.055687011
c77	GO:0044085~cellular component biogenesis	0.055751949
c77	GO:0016071~mRNA metabolic process	0.056461202
c77	GO:0051248~negative regulation of protein metabolic process	0.059129656
c77	GO:0031324~negative regulation of cellular metabolic process	0.060533012
c77	GO:0006325~chromatin organization	0.062382565
c77	GO:0016310~phosphorylation	0.064699147
c77	GO:0006047~UDP-N-acetylglucosamine metabolic process	0.065317653
c77	GO:0018346~protein amino acid prenylation	0.065317653
c77	GO:0006996~organelle organization	0.068015048
c77	GO:0031398~positive regulation of protein ubiquitination	0.068056141
c77	GO:0006397~mRNA processing	0.069307778
c77	GO:0043687~post-translational protein modification	0.069328032
c77	GO:0006468~protein amino acid phosphorylation	0.071215787
c77	GO:0048523~negative regulation of cellular process	0.074873224
c77	GO:0015879~carnitine transport	0.075783975
c77	GO:0015838~betaine transport	0.075783975
c77	GO:0018342~protein prenylation	0.075783975
c77	GO:0006865~amino acid transport	0.08002848
c77	GO:0044092~negative regulation of molecular function	0.081643863
c77	GO:0007093~mitotic cell cycle checkpoint	0.083469405
c77	GO:0046942~carboxylic acid transport	0.083490117

c77	GO:0000226~microtubule cytoskeleton organization	0.083490117
c77	GO:0015849~organic acid transport	0.084536526
c77	GO:0046785~microtubule polymerization	0.086133832
c77	GO:0051246~regulation of protein metabolic process	0.086482394
c77	GO:0016043~cellular component organization	0.087804723
c77	GO:0034641~cellular nitrogen compound metabolic process	0.089588135
c77	GO:0007051~spindle organization	0.090218016
c77	GO:0007346~regulation of mitotic cell cycle	0.09107129
c77	GO:0002566~somatic diversification of immune receptors via somatic mutation	0.096368511
c77	GO:0016446~somatic hypermutation of immunoglobulin genes	0.096368511
c77	GO:0006807~nitrogen compound metabolic process	0.097650382
c9	GO:0060249~anatomical structure homeostasis	0.003208396
c9	GO:0051240~positive regulation of multicellular organismal process	0.013091226
c9	GO:0043174~nucleoside salvage	0.022758271
c9	GO:0006166~purine ribonucleoside salvage	0.022758271
c9	GO:0043101~purine salvage	0.028367439
c9	GO:0006950~response to stress	0.028431343
c9	GO:0007154~cell communication	0.037972386
c9	GO:0016255~attachment of GPI anchor to protein	0.039490552
c9	GO:0010647~positive regulation of cell communication	0.040633084
c9	GO:0032946~positive regulation of mononuclear cell proliferation	0.041021644
c9	GO:0070665~positive regulation of leukocyte proliferation	0.041021644
c9	GO:0033554~cellular response to stress	0.04355753
c9	GO:0009163~nucleoside biosynthetic process	0.045004853
c9	GO:0042455~ribonucleoside biosynthetic process	0.045004853
c9	GO:0007217~tachykinin receptor signaling pathway	0.045004853
c9	GO:0046129~purine ribonucleoside biosynthetic process	0.045004853
c9	GO:0042451~purine nucleoside biosynthetic process	0.045004853
c9	GO:0043094~cellular metabolic compound salvage	0.045004853
c9	GO:0006302~double-strand break repair	0.04928084
c9	GO:0001894~tissue homeostasis	0.050711568
c9	GO:0009987~cellular process	0.057509743
c9	GO:0006974~response to DNA damage stimulus	0.063088532
c9	GO:0009966~regulation of signal transduction	0.063729879
c9	GO:0006809~nitric oxide biosynthetic process	0.06675111
c9	GO:0051241~negative regulation of multicellular organismal process	0.068182652
c9	GO:0006259~DNA metabolic process	0.070323601
c9	GO:0046209~nitric oxide metabolic process	0.072110807
c9	GO:0030318~melanocyte differentiation	0.0774401
c9	GO:0006281~DNA repair	0.080412072
c9	GO:0050931~pigment cell differentiation	0.082739161
c9	GO:0001775~cell activation	0.082836995
c9	GO:0032944~regulation of mononuclear cell proliferation	0.083884538
c9	GO:0070663~regulation of leukocyte proliferation	0.083884538
c9	GO:0048871~multicellular organismal homeostasis	0.085596686

c9	GO:0001817~regulation of cytokine production	0.085813923
c9	GO:0051239~regulation of multicellular organismal process	0.087626843
c9	GO:0009967~positive regulation of signal transduction	0.089479101
c78	GO:0007588~excretion	0.020378796
c78	GO:0003008~system process	0.091069401
c80	GO:0040008~regulation of growth	0.001989197
c80	GO:0009166~nucleotide catabolic process	0.003211556
c80	GO:0034656~nucleobase, nucleoside and nucleotide catabolic process	0.006107652
c80	GO:0034655~nucleobase, nucleoside, nucleotide and nucleic acid catabolic process	0.006107652
c80	GO:0040029~regulation of gene expression, epigenetic	0.00642706
c80	GO:0051276~chromosome organization	0.006682674
c80	GO:0009134~nucleoside diphosphate catabolic process	0.011355381
c80	GO:0043170~macromolecule metabolic process	0.011906275
c80	GO:0044238~primary metabolic process	0.011948312
c80	GO:0044270~nitrogen compound catabolic process	0.012162022
c80	GO:0016568~chromatin modification	0.012605492
c80	GO:0048015~phosphoinositide-mediated signaling	0.013009296
c80	GO:0006342~chromatin silencing	0.013792824
c80	GO:0006139~nucleobase, nucleoside, nucleotide and nucleic acid metabolic process	0.013946529
c80	GO:0000279~M phase	0.01401773
c80	GO:0046483~heterocycle metabolic process	0.016043143
c80	GO:0030162~regulation of proteolysis	0.017447049
c80	GO:0009987~cellular process	0.017825202
c80	GO:0006163~purine nucleotide metabolic process	0.018214965
c80	GO:0010605~negative regulation of macromolecule metabolic process	0.019472052
c80	GO:0006259~DNA metabolic process	0.019859868
c80	GO:0034984~cellular response to DNA damage stimulus	0.020407849
c80	GO:0022402~cell cycle process	0.020914401
c80	GO:0045814~negative regulation of gene expression, epigenetic	0.021106112
c80	GO:0016458~gene silencing	0.02167554
c80	GO:0045934~negative regulation of nucleobase, nucleoside, nucleotide and nucleic acid metabolic process	0.022358094
c80	GO:0034641~cellular nitrogen compound metabolic process	0.023520711
c80	GO:0006325~chromatin organization	0.024424101
c80	GO:0006807~nitrogen compound metabolic process	0.025069795
c80	GO:0031324~negative regulation of cellular metabolic process	0.025350573
c80	GO:0051172~negative regulation of nitrogen compound metabolic process	0.025977414
c80	GO:0043085~positive regulation of catalytic activity	0.026359685
c80	GO:0050801~ion homeostasis	0.026520505
c80	GO:0045927~positive regulation of growth	0.026957518
c80	GO:0000187~activation of MAPK activity	0.029973631
c80	GO:0022403~cell cycle phase	0.030042286
c80	GO:0009143~nucleoside triphosphate catabolic process	0.030164074
c80	GO:0044093~positive regulation of molecular function	0.030365976
c80	GO:0045862~positive regulation of proteolysis	0.033569433
c80	GO:0006281~DNA repair	0.034446168

c80	GO:0009141~nucleoside triphosphate metabolic process	0.034596158
c80	GO:0044260~cellular macromolecule metabolic process	0.036078728
c80	GO:0007049~cell cycle	0.038292165
c80	GO:0048878~chemical homeostasis	0.038389634
c80	GO:0055086~nucleobase, nucleoside and nucleotide metabolic process	0.038838855
c80	GO:0051338~regulation of transferase activity	0.039648245
c80	GO:0009892~negative regulation of metabolic process	0.040392278
c80	GO:0042592~homeostatic process	0.04045448
c80	GO:0006284~base-excision repair	0.04095052
c80	GO:0006873~cellular ion homeostasis	0.041305132
c80	GO:0006950~response to stress	0.042339084
c80	GO:0010558~negative regulation of macromolecule biosynthetic process	0.042452626
c80	GO:0006753~nucleoside phosphate metabolic process	0.042914752
c80	GO:0009117~nucleotide metabolic process	0.042914752
c80	GO:0051347~positive regulation of transferase activity	0.043825712
c80	GO:0008156~negative regulation of DNA replication	0.044921917
c80	GO:0000723~telomere maintenance	0.044921917
c80	GO:0055082~cellular chemical homeostasis	0.046728702
c80	GO:0007275~multicellular organismal development	0.046986457
c80	GO:0030258~lipid modification	0.0473936
c80	GO:0016573~histone acetylation	0.047922811
c80	GO:0032200~telomere organization	0.049077068
c80	GO:0001558~regulation of cell growth	0.05273333
c80	GO:0046620~regulation of organ growth	0.053412966
c80	GO:0032502~developmental process	0.053740834
c80	GO:0031327~negative regulation of cellular biosynthetic process	0.053923059
c80	GO:0006937~regulation of muscle contraction	0.055124355
c80	GO:0045860~positive regulation of protein kinase activity	0.05641569
c80	GO:0032214~negative regulation of telomere maintenance via semi-conservative replication	0.056422876
c80	GO:0001304~progressive alteration of chromatin during replicative cell aging	0.056422876
c80	GO:0032213~regulation of telomere maintenance via semi-conservative replication	0.056422876
c80	GO:0001309~age-dependent telomere shortening	0.056422876
c80	GO:0001301~progressive alteration of chromatin during cell aging	0.056422876
c80	GO:0051253~negative regulation of RNA metabolic process	0.057296358
c80	GO:0044237~cellular metabolic process	0.057677877
c80	GO:0000087~M phase of mitotic cell cycle	0.057917677
c80	GO:0046112~nucleobase biosynthetic process	0.059259294
c80	GO:0031323~regulation of cellular metabolic process	0.060011198
c80	GO:0016570~histone modification	0.061032911
c80	GO:0006473~protein amino acid acetylation	0.061095074
c80	GO:0009890~negative regulation of biosynthetic process	0.064060633
c80	GO:0006304~DNA modification	0.067470978
c80	GO:0046324~regulation of glucose import	0.067470978
c80	GO:0055080~cation homeostasis	0.068367205
c80	GO:0008152~metabolic process	0.06871151

c80	GO:0051716~cellular response to stimulus	0.068752624
c80	GO:0033674~positive regulation of kinase activity	0.068884007
c80	GO:0016569~covalent chromatin modification	0.070011875
c80	GO:0043406~positive regulation of MAP kinase activity	0.072392813
c80	GO:0010827~regulation of glucose transport	0.072494519
c80	GO:0016043~cellular component organization	0.074613053
c80	GO:0019932~second-messenger-mediated signaling	0.07547396
c80	GO:0018394~peptidyl-lysine acetylation	0.083431886
c80	GO:0030730~sequestering of triglyceride	0.083431886
c80	GO:0009181~purine ribonucleoside diphosphate catabolic process	0.083431886
c80	GO:0009137~purine nucleoside diphosphate catabolic process	0.083431886
c80	GO:0046100~hypoxanthine metabolic process	0.083431886
c80	GO:0010834~telomere maintenance via telomere shortening	0.083431886
c80	GO:0018076~N-terminal peptidyl-lysine acetylation	0.083431886
c80	GO:0060249~anatomical structure homeostasis	0.083638058
c80	GO:0009132~nucleoside diphosphate metabolic process	0.083766633
c80	GO:0019222~regulation of metabolic process	0.08620248
c80	GO:0045892~negative regulation of transcription, DNA-dependent	0.088122099
c80	GO:0051349~positive regulation of lyase activity	0.08823077
c80	GO:0033554~cellular response to stress	0.089408115
c80	GO:0065009~regulation of molecular function	0.091723098
c80	GO:0043543~protein amino acid acylation	0.092513271
c80	GO:0042493~response to drug	0.092549012
c80	GO:0050790~regulation of catalytic activity	0.093454989
c80	GO:0006940~regulation of smooth muscle contraction	0.094164498
c80	GO:0031325~positive regulation of cellular metabolic process	0.094927655
c80	GO:0040014~regulation of multicellular organism growth	0.096892883
c80	GO:0051053~negative regulation of DNA metabolic process	0.099953316
c81	GO:0008361~regulation of cell size	0.023167296
c81	GO:0030308~negative regulation of cell growth	0.030558986
c81	GO:0045792~negative regulation of cell size	0.03495614
c81	GO:0006525~arginine metabolic process	0.038012353
c81	GO:0045926~negative regulation of growth	0.042327517
c81	GO:0032535~regulation of cellular component size	0.04639067
c81	GO:0048519~negative regulation of biological process	0.081789921
c81	GO:0043436~oxoacid metabolic process	0.082237828
c81	GO:0019752~carboxylic acid metabolic process	0.082237828
c81	GO:0006082~organic acid metabolic process	0.083929713
c81	GO:0042180~cellular ketone metabolic process	0.086932434
c10	GO:0070228~regulation of lymphocyte apoptosis	0.002860776
c10	GO:0006308~DNA catabolic process	0.003713623
c10	GO:0006259~DNA metabolic process	0.004188576
c10	GO:0016070~RNA metabolic process	0.006509816
c10	GO:0051301~cell division	0.007316519
c10	GO:0007049~cell cycle	0.011491679

c10	GO:0006281~DNA repair	0.012053071
c10	GO:0048285~organelle fission	0.012520402
c10	GO:0002902~regulation of B cell apoptosis	0.015718169
c10	GO:0022403~cell cycle phase	0.016347722
c10	GO:0000279~M phase	0.018371448
c10	GO:0043270~positive regulation of ion transport	0.020196689
c10	GO:0006629~lipid metabolic process	0.020702813
c10	GO:0000280~nuclear division	0.022399613
c10	GO:0007067~mitosis	0.022399613
c10	GO:0006298~mismatch repair	0.023555195
c10	GO:0006487~protein amino acid N-linked glycosylation	0.023569187
c10	GO:0007017~microtubule-based process	0.024776373
c10	GO:0000087~M phase of mitotic cell cycle	0.025171497
c10	GO:0019725~cellular homeostasis	0.025281478
c10	GO:0006974~response to DNA damage stimulus	0.025392688
c10	GO:0006399~tRNA metabolic process	0.028285032
c10	GO:0051924~regulation of calcium ion transport	0.028720362
c10	GO:0034660~ncRNA metabolic process	0.030074346
c10	GO:0000226~microtubule cytoskeleton organization	0.03101925
c10	GO:0051604~protein maturation	0.033079833
c10	GO:0006916~anti-apoptosis	0.034143786
c10	GO:0010522~regulation of calcium ion transport into cytosol	0.034800283
c10	GO:0051327~M phase of meiotic cell cycle	0.035797597
c10	GO:0007126~meiosis	0.035797597
c10	GO:0051321~meiotic cell cycle	0.038927962
c10	GO:0006665~sphingolipid metabolic process	0.039159988
c10	GO:0045822~negative regulation of heart contraction	0.040340891
c10	GO:0030148~sphingolipid biosynthetic process	0.041289302
c10	GO:0000278~mitotic cell cycle	0.044936993
c10	GO:0022402~cell cycle process	0.044992713
c10	GO:0033554~cellular response to stress	0.045521113
c10	GO:0006304~DNA modification	0.048341515
c10	GO:0006873~cellular ion homeostasis	0.048474358
c10	GO:0046666~retinal cell programmed cell death	0.049252392
c10	GO:0006643~membrane lipid metabolic process	0.051641919
c10	GO:0010959~regulation of metal ion transport	0.051641919
c10	GO:0007059~chromosome segregation	0.051641919
c10	GO:0046467~membrane lipid biosynthetic process	0.052074281
c10	GO:0046513~ceramide biosynthetic process	0.05256673
c10	GO:0019363~pyridine nucleotide biosynthetic process	0.05256673
c10	GO:0051279~regulation of release of sequestered calcium ion into cytosol	0.05256673
c10	GO:0008272~sulfate transport	0.05256673
c10	GO:0055082~cellular chemical homeostasis	0.054114254
c10	GO:0044238~primary metabolic process	0.054219835
c10	GO:0006352~transcription initiation	0.056263119

c10	GO:0034641~cellular nitrogen compound metabolic process	0.056537843
c10	GO:0006396~RNA processing	0.056573417
c10	GO:0046520~sphingoid biosynthetic process	0.059110092
c10	GO:0042308~negative regulation of protein import into nucleus	0.059110092
c10	GO:0031124~mRNA 3'-end processing	0.059110092
c10	GO:0009081~branched chain family amino acid metabolic process	0.059110092
c10	GO:0031348~negative regulation of defense response	0.05994256
c10	GO:0016485~protein processing	0.061316325
c10	GO:0010043~response to zinc ion	0.065916426
c10	GO:0008152~metabolic process	0.066993501
c10	GO:0044255~cellular lipid metabolic process	0.068100624
c10	GO:0070296~sarcoplasmic reticulum calcium ion transport	0.072963824
c10	GO:0034501~protein localization to kinetochore	0.072963824
c10	GO:0046823~negative regulation of nucleocytoplasmic transport	0.072968633
c10	GO:0044106~cellular amine metabolic process	0.081123511
c10	GO:0032507~maintenance of protein location in cell	0.081847772
c10	GO:0010608~posttranscriptional regulation of gene expression	0.082638276
c10	GO:0006807~nitrogen compound metabolic process	0.08546592
c10	GO:0051716~cellular response to stimulus	0.085922007
c10	GO:0007346~regulation of mitotic cell cycle	0.086273574
c10	GO:0006672~ceramide metabolic process	0.086590163
c10	GO:0044085~cellular component biogenesis	0.087690466
c10	GO:0010524~positive regulation of calcium ion transport into cytosol	0.087745664
c10	GO:0050801~ion homeostasis	0.088310253
c10	GO:0046474~glycerophospholipid biosynthetic process	0.089594643
c10	GO:0009987~cellular process	0.091850688
c10	GO:0031099~regeneration	0.093340752
c10	GO:0046470~phosphatidylcholine metabolic process	0.095439622
c10	GO:0001836~release of cytochrome c from mitochondria	0.095439622
c10	GO:0006269~DNA replication, synthesis of RNA primer	0.096085537
c10	GO:0032455~nerve growth factor processing	0.096085537
c10	GO:0006551~leucine metabolic process	0.096085537
c10	GO:0002904~positive regulation of B cell apoptosis	0.096085537
c10	GO:0034470~ncRNA processing	0.096961359
